# Collaborative Synthesis for Neglected Diseases through the Open Synthesis Network: Structure–Activity Relationships of Arylaminopyrazoles as Chagas Disease Treatments

**DOI:** 10.1021/acsinfecdis.5c00481

**Published:** 2025-08-11

**Authors:** Zigli Abdulai, Natasha Agbo, Jonathan I Anderson, Faye Astley, Brian Chan, Owen Atkinson-Evans, João L Avelar, Cristiane Aparecida-Silva, KD Bhardwaj, William J Bowley, Nicholas Breitkreuz, Rosie Canby, Emerald Cartwright, Charles Clifford, Shannon A Cordell, William J Donker, Joseph Driscoll, Matthew Grady, Lauren Higginbotham, Devon Hsu, Jamie Hutchinson, Lukas Imberg, Harry F Jackson, Finan Johns, Emma Jones, Dmitrii V. Kalinin, Ceren Kardeşler, Aaron Keal, Libby Keel, Sumin Kim, Phoebe Knight, Justus F. Ködel, Luke Kumeta, Hyejin Lee, Sam Le Roy, Rachelle Maccarone, Maria Mahmud, Matthew Martin, Ignatius Nguyen, Conor J Nolan, Lucy Noyes, Angela N. W Ntuwa, Wiktoria Obarska, Owen Oldham, Edna Onyiuke, Jess Otter, Hugh Page, Dhruvi Patel, Kayla Reid, Krishna Samaddar, Sheryar M Shabbir, Peter Shevlin, Callum Sinclair-Wright, Amanda Smithies, Amelia S Thomson, Jack Tinker, Alexander Uner, Natascha Van Pelt, Ewan Waddell, Hamis Wagwa, Claire Walthorne, Sophie Warner, Tobias Winge, Ngai Sum Wong, Leon Jan Wysocki, Callum A Yong, Zaria Zaheen, An Matheeussen, Guy Caljon, Richard Amewu, Anna Bertram, Bernhard Biersack, Carolyn Friel, Lídia Moreira Lima, Chase Smith, Bernhard Wünsch, Benjamin Perry, Luiza R. Cruz, Andrew Nortcliffe

**Affiliations:** † GlaxoSmithKline Carbon Neutral Laboratories for Sustainable Chemistry, School of Chemistry, 6123 University of Nottingham, Triumph Road, Nottingham NG7 2TU, U.K.; ‡ School of Chemistry, University of Nottingham, University Park, Nottingham NG7 2RD, U.K.; § 58076 Drugs for Neglected Diseases Initiative, 15 Chemin Camille Vidart, Geneva 1202, Switzerland; ∥ Laboratório de Avaliação e Síntese de Substâncias Bioativas, Centro de Ciências da Saúde, Universidade Federal do Rio de Janeiro, Cidade Universitária, Rio de Janeiro, Rio de Janeiro 20000-000, Brazil; ⊥ Department of Pharmaceutical Sciences, School of Pharmacy-Worcester/Manchester, 1825 Massachusetts College of Pharmacy and Health Sciences, 19 Foster Street, Worcester, Massachusetts 01608, United States of America; # Department of Chemistry, 58835 University of Ghana, P.O. Box LG56, Legon, Accra LG56, Ghana; ¶ 9185 Institut für Pharmazeutische und Medizinische Chemie der Westfälischen Wilhelms-Universität Münster, Corrensstraße 48, Münster D-48149, Germany; ∇ Organic Chemistry Laboratory, 26523 University of Bayreuth, Universitätsstrasse 30, Bayreuth 95440, Germany; ○ Laboratory of Microbiology, Parasitology and Hygiene (LMPH), 26660 University of Antwerp, Universiteitsplein 1, Wilrijk B-2610, Belgium; ⧫ Drugs for Neglected Diseases Initiative, Rio de Janeiro 20010020, Brazil

**Keywords:** infectious diseases, parasites, drug discovery, education, Trypanosoma cruzi, structure−activity relationships, NTDs, open innovation

## Abstract

Neglected tropical diseases (NTDs) make up a diverse group of debilitating illnesses disproportionately affecting impoverished communities in tropical and subtropical regions. Despite their significant global health burden, they are often overshadowed by more prominent diseases, resulting in a critical lack of investment in the research and development of new treatments. A renewed focus on NTDs is, therefore, urgently needed, particularly in terms of novel therapeutic strategies. The Open Synthesis Network, launched by DNDi and partner institutions in 2016, is an innovation powerhouse that taps into the potential of students to help drive the discovery of new drugs for patients living with NTDs. We present the results of student-led work into the development of a series of aminopyrazoles for Chagas disease, a multisystemic disease caused by the *Trypanosoma cruzi* parasite. Seventy-four compounds were synthesized by undergraduate and postgraduate students from six universities from Brazil, Ghana, Germany, USA, and UK, illustrating that open innovation and collaboration for education can drive drug discovery forward. Early evaluation of the structure–activity relationships identified a range of potent hit compounds with selectivity for *T. cruzi* and no observable cytotoxicity.

## Graphical abstract

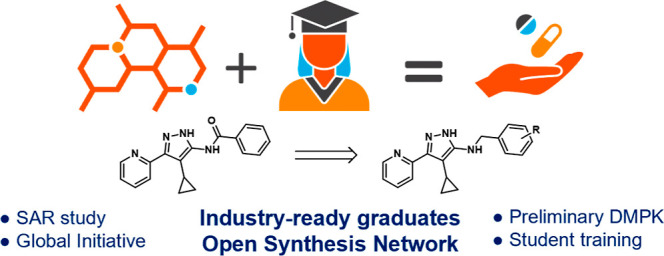

The Drugs for Neglected Diseases initiative (DNDi) is an international not-for-profit research and development (R&D) organization that leads international collaborations with pharmaceutical and academic partners to contribute to drug discovery in neglected tropical diseases (NTDs). In 2016, DNDi established the Open Synthesis Network (OSN) as a global collaborative initiative to harness the capacity of chemistry teaching laboratories to help discover new drugs for patients living with NTDs. The OSN engages with undergraduate and master’s-level students to carry out early-stage research. Students are given real problems to investigate from DNDi’s R&D portfolio, developing their synthetic chemistry skills while furthering their understanding of drug discovery and medicinal chemistry through active learning. Since its launch, more than 1200 compounds have been synthesized by students from more than 30 different academic institutions in ten countries around the globe.

Mirroring DNDi’s own R&D portfolio, the OSN has a clear focus on three main diseases caused by a group of flagellated protozoans: Chagas disease (CD), caused by *Trypanosoma cruzi*; leishmaniasis, caused by *Leishmania spp*.; and human African trypanosomiasis (HAT), also known as sleeping sickness, caused by two subspecies of *Trypanosoma brucei*.^1^ All compounds made by OSN students have parasitology data against these parasites (as well as cytotoxicity in the relevant cell lines) provided by the Laboratory of Microbiology, Parasitology, and Hygiene (LMPH) at the University of Antwerp in Belgium.

Within a drug discovery program, medicinal chemists are required to design, synthesize, and test significant numbers of diverse compounds within a chemical series. This generates information about the chemical series, contributing to one of the core tenets of medicinal chemistry, the SAR. The empirical approach to medicinal chemistry design (“what happens if I make this change?”) lends itself particularly well to parallel, matrix-based synthetic chemistry in which multiple analogues around a particular series are exemplified via the same fundamental synthetic route using a diversity of key reagents or building blocks to explore the resulting SAR. This is particularly pertinent in the field of NTDs, where traditionally drug discovery projects are based on phenotypic campaigns. Most of DNDi’s discovery projects start as phenotypic projects, building on data sets from the screening of large libraries. This pairs well with academic teaching laboratories, where students can synthesize, in parallel, multiple variants around a particular structural motif by the simple variation of a key building block.

Open and collaborative platforms such as the OSN are a strategy to overcome the lack of commercial interest in new treatments for NTDs. Furthermore, since no protective intellectual property, such as patents, for new chemical matter is required, the participation of universities and their students in collaborative endeavors is vastly simplified. As an open-source science project, all data generated by OSN is published in the public domain in real-time and remains free of intellectual property.

A series of aminopyrazoles, previously investigated as potential therapeutics for visceral leishmaniasis, resulted in preclinical candidate DNDI-5561. Unfortunately, due to unfavorable safety results in preclinical studies, the development of DNDI-5561 was halted in 2019.^2,3^ The goal of this OSN project was to identify the next generation of aminopyrazole compounds by exploring emerging *T. cruzi* activity from a benzylaminopyrazole series identified during the development of DNDI-5561 when removing the carbonyl in amide **1** and in urea **2** (Figure 1). Herein, we detail the optimization of the anti-*T. cruzi* activity in the P1Tc project.

**Figure 1:**
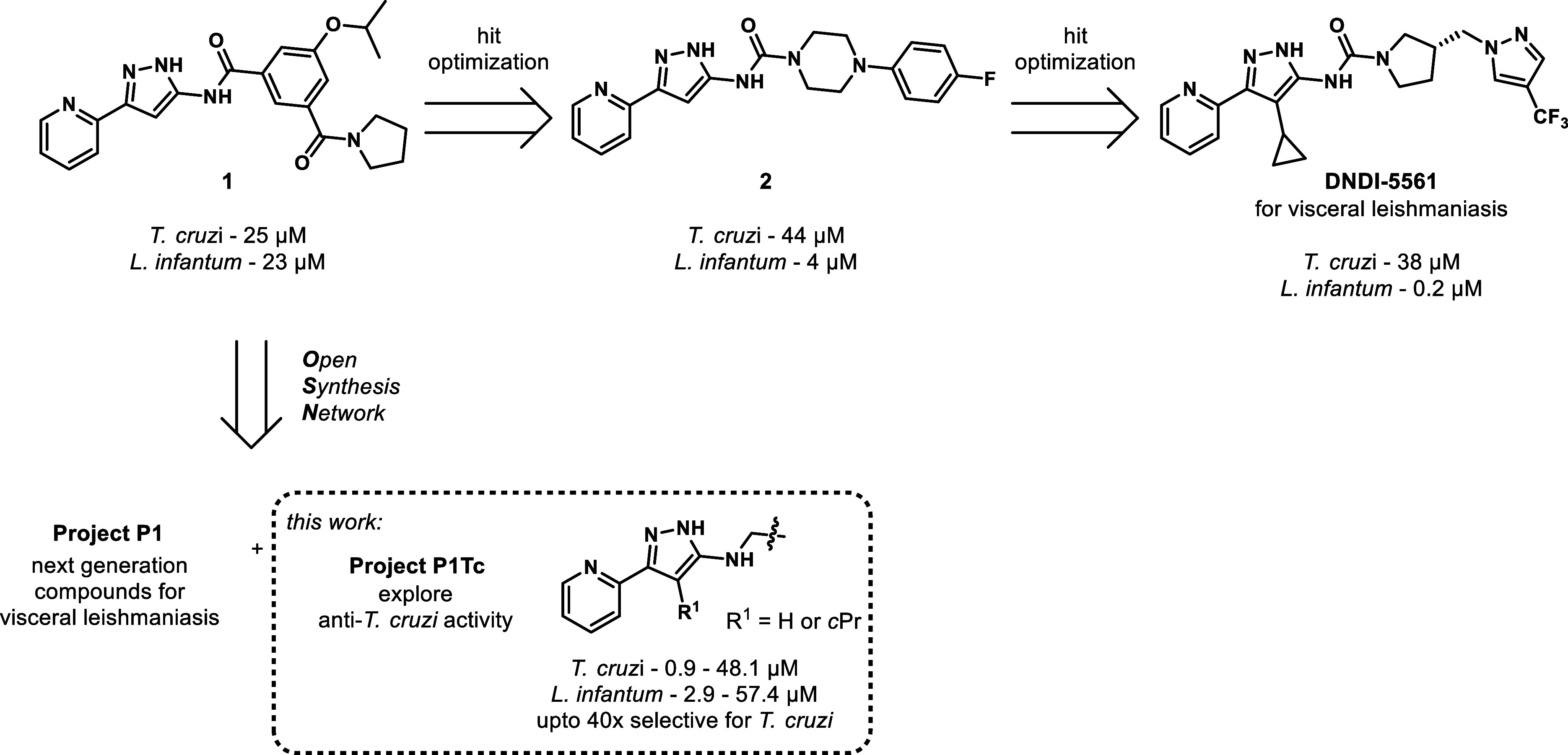
Open Synthesis Network’s P1Tc project.

CD, also known as American trypanosomiasis, is a multisystemic disease caused by *T. cruzi*.^4^ CD is transmitted through various species of hematophagous insects, in particular the triatomine bugs of the *Reduviidae* family. It can also be contracted through contaminated blood transfusion, organ transplantation and congenital infection.^4^ CD is endemic to Latin America, with an estimated 6–7 million people infected with *T. cruzi*, and mortality in Latin America is greater than for any other parasitic disease, including malaria.^5^ In recent decades, the epidemiological pattern of the disease has altered due to population mobility, urbanization, and migration.^6,7^ As a consequence, CD is now prevalent across southern North America,^8^ Europe,^9^ and the Western Pacific, including Japan.^10^ An estimated 70 million people in the Americas are at risk of contracting CD.

CD is a highly complex disease. While traditionally thought to have three disease phases (acute, chronic indeterminate/asymptomatic, and chronic symptomatic), infection is highly heterogeneous and manifests differently in each patient.^11^ The acute phase, which lasts about six to 8 weeks, is often not recognized due to the nonspecific symptoms of infection (fever, weight loss, and/or flu-like symptoms) or because it is asymptomatic, despite increasing *T. cruzi* parasitemia.^12^ A parasite-specific adaptive immunity develops following the acute phase, leading to low-level blood parasitemia for life. From here, patients may progress to the indeterminate or symptomatic stages. Approximately one-third of those infected develop disease-related cardiomyopathy or megacolon disease in the chronic phase.^13,14^

Acute *T. cruzi* infection is currently treated with either benznidazole **3** or nifurtimox **4** (Figure 2), which can be curative if administered soon after infection at the onset of the acute phase of the disease.^15^ The efficacy of both agents diminishes with persistent infection, and adverse reactions are more frequent in older age groups.^16^ Both agents are contraindicated in pregnancy.^17^

**Figure 2:**
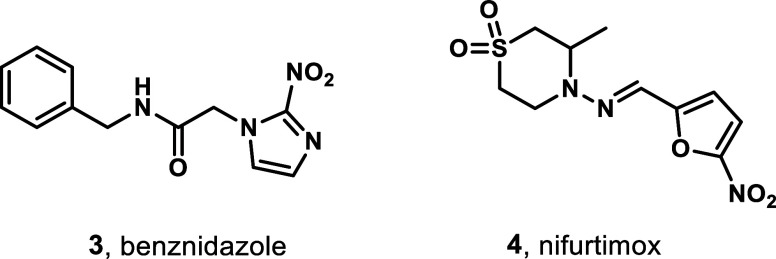
Chemical structures of benznidazole **3** and nifurtimox **4**.

While there have been continued efforts by the World Health Organization (WHO) to reduce transmission by implementing parasite/vector control,^18^ CD remains an invisible NTD. Clinical failures of the CYP51 inhibitors posocanozole^19^ and E1224 ^19^ (a prodrug of ravuconazole), as well as failure of the nitroimidazole fexinidazole, show how difficult it is to develop new drugs to treat the disease.^20^ Hence, there is a pressing need for new, safe, effective, and affordable oral chemotherapeutic agents that show superior activity to the current standard of care but also for new development models and strategies.

Our work within the OSN aims to optimize early hits with antitrypanosomal activity to identify the potential for lead optimization and hopefully progression to nominate a preclinical candidate that meets the target profile.^21^ While development of a selective agent for *T. cruzi* is not an essential criterion, it may serve as a strategic advantage by reducing the risk of cross-resistance between kinetoplastid parasites, e.g., *L. infantum*.^22^ Moreover, given the current shift in global drug discovery priorities particularly the reduced emphasis on leishmaniasis by DNDi and other stakeholders—such selectivity aligns with a portfolio strategy focused on addressing unmet needs in Chagas disease.

## Synthesis

The synthesis of *N*-benzylaminopyrazole derivatives was straightforward (Scheme 1). Cyclopropyl derivative **5** ^3^ was alkylated under standard reductive amination conditions with a range of commercially available aldehydes to generate a diverse library of substituted N-substituted products **6a**–**6bl** (Scheme 1).

**Scheme 1:**
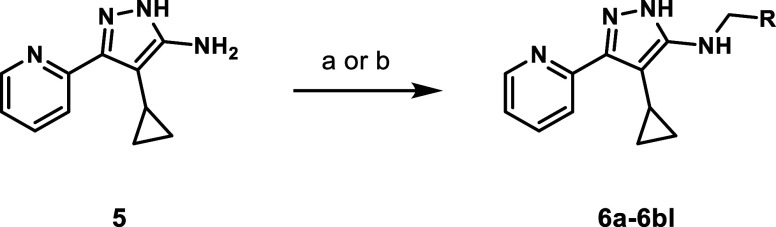
General Synthesis of Cyclopropyl-*N*-Benzylaminopyrazoles^a^

a Reagents and conditions: (a) Aldehyde, sodium triacetoxyborohydride, acetic acid, 4 Å molecular sieves, 1,2-dichloroethane, r.t. 16 h, 6–81%; (b) Aldehyde, CH_2_Cl_2_, Reflux, 4 h, Then NaBH_4_, MeOH, r.t., 1–18 h, 35–73%. R = (hetero)­aromatic or aliphatic.

Unsubstituted pyrazole analogues **8a**–**g** were prepared from pyrazole **7**. Preformed imines were reduced with sodium borohydrides to give the desired products in 42–87% yield (Scheme 2).

**Scheme 2:**
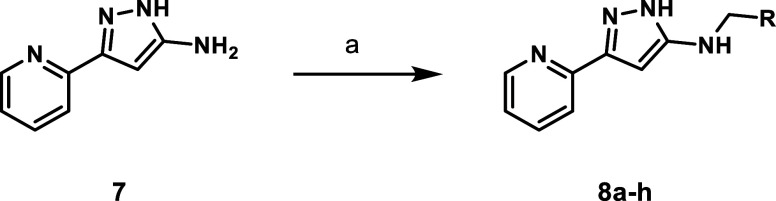
General Synthesis of Unsubstituted *N*-Benzylaminopyrazoles^a^

a Reagents and conditions: (a) (i). Aldehyde, HCl, THF, r.t., (ii) NaBH_4_, r.t., 16 h, 42–87%. R = Aromatic

Two pyrazole analogues were prepared to explore structure–activity relationship (SAR) of the 2-pyridyl portion of the scaffold. Briefly, methyl esters **9** and **10** were first treated with the anion of acetonitrile to give the corresponding β-ketonitriles, followed by cyclization with hydrazine to provide the respective aminopyrazoles **11** and **12**. Reductive amination with biphenyl-4-carboxaldehyde provided the pyrazoles **13** and **14** (Scheme 3).

**Scheme 3:**
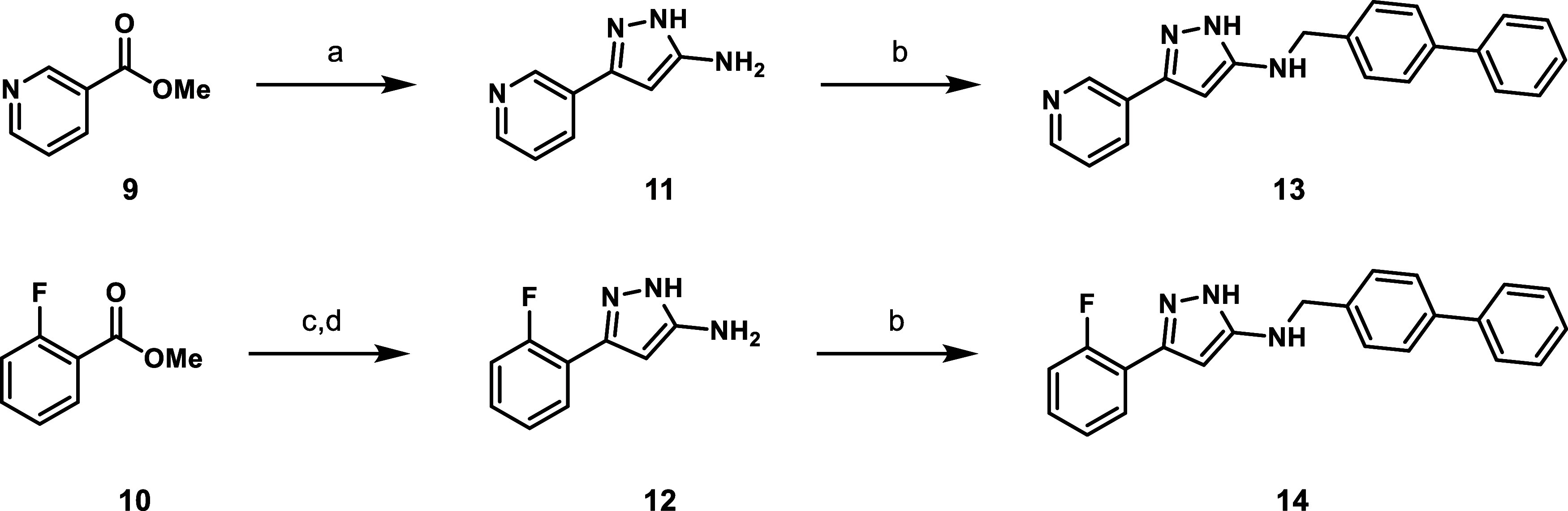
Synthesis of *N*-Benzylaminopyrazoles Varying the Pyridyl Ring

a Reagents and conditions: (a) (i). NaH, CH_3_CN, THF, 80 °C, 16 h; (ii). hydrazine Hydrate, EtOH, 80 °C, 16 h, 53% Over Two Steps; (b) (i). Biphenyl-4-Carboxaldehyde, Acetic Acid, 3 Å Molecular Sieves, EtOH, r.t., 22 h; (ii). NaBH_4_, r.t., 16 h, **13** = 72%, **14** = 69%; (c) nBuLi, CH_3_CN, THF, −78 °C to r.t., 16 h, 91%; (d) Hydrazine Hydrate, EtOH, 90 °C, 16 h, 81%

## Results and Discussion

The compounds synthesized were evaluated for antileishmanial and antitrypanosomal activity (Supporting Information). Briefly, in the antileishmanial assay, test compounds were incubated with the macrophage *L. infantum* (MHOM/MA­(BE)/67 strain) inoculum. After an incubation period of 5 days, the parasite burden of the macrophages was determined microscopically after staining with 10% Giemsa solution. The reference compounds miltefosine and amphotericin B were included in the *L. infantum* assay.^23^ In order to determine the antitrypanosomal activity, test compounds were incubated with human fetal lung fibroblasts (MRC-5) cells inoculated with *T. cruzi* (Tulahuen CL2, β-galactosidase strain) at 37 °C for 5 days. After the additions of chlorophenol red β- *d* -galactopyranoside (CPRG), the number of parasites was calculated from the intensity of the color formed (λ_max_ = 540 nm). Benznidazole and nifurtimox served as a reference compound in the antitrypanosomal assay. The unspecific toxicity was determined by incubation of the test compounds with noninfected MRC-5 and PMM cells. Table 1 displays the results of the biological assays performed with the test compounds **6a**–**6bl**.

**Table 1:**
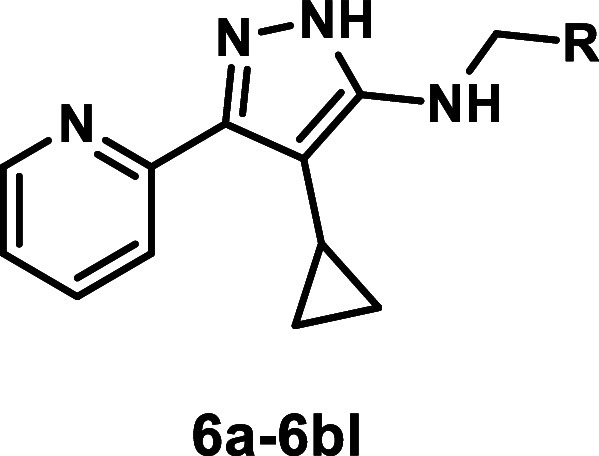

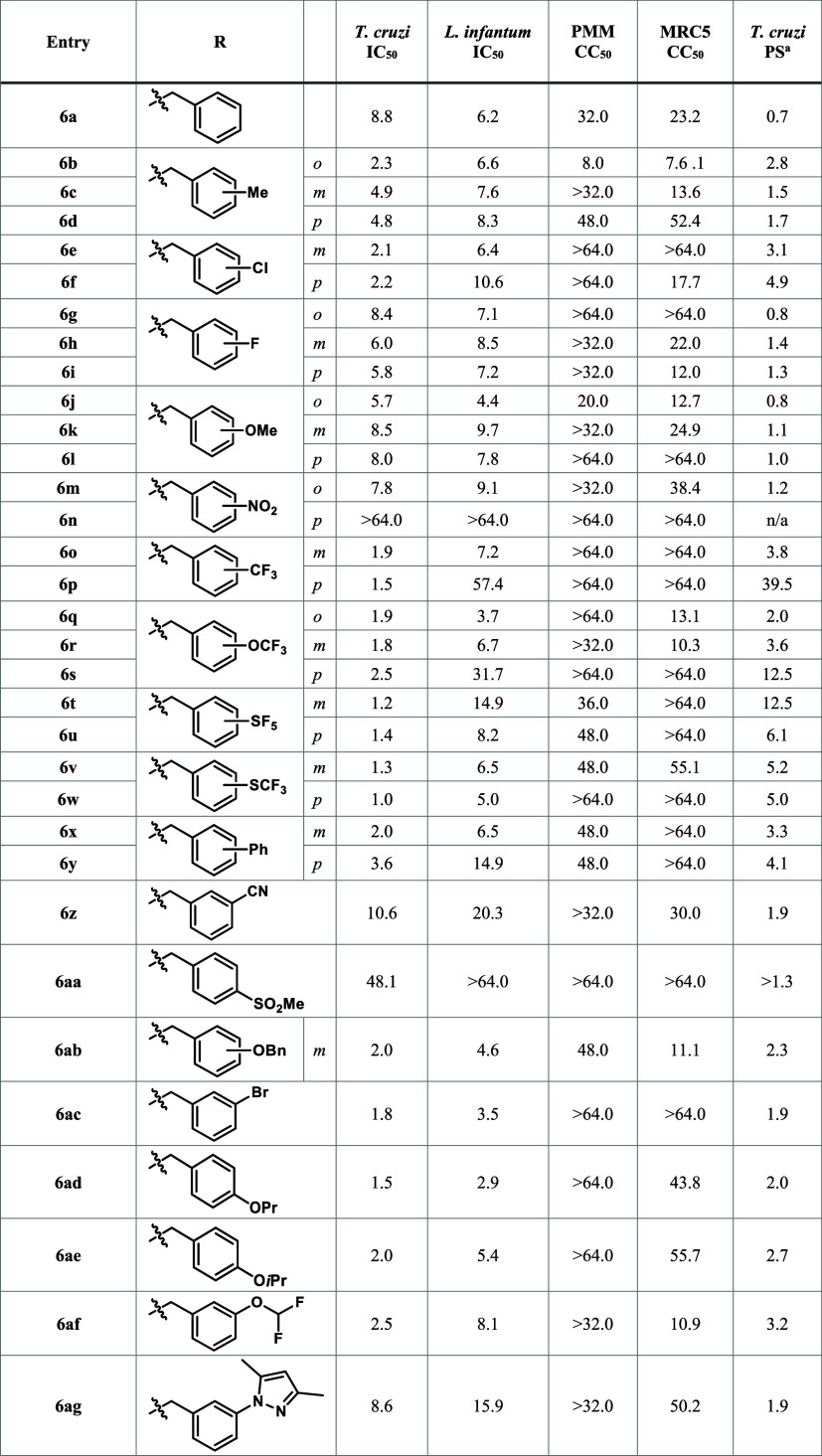

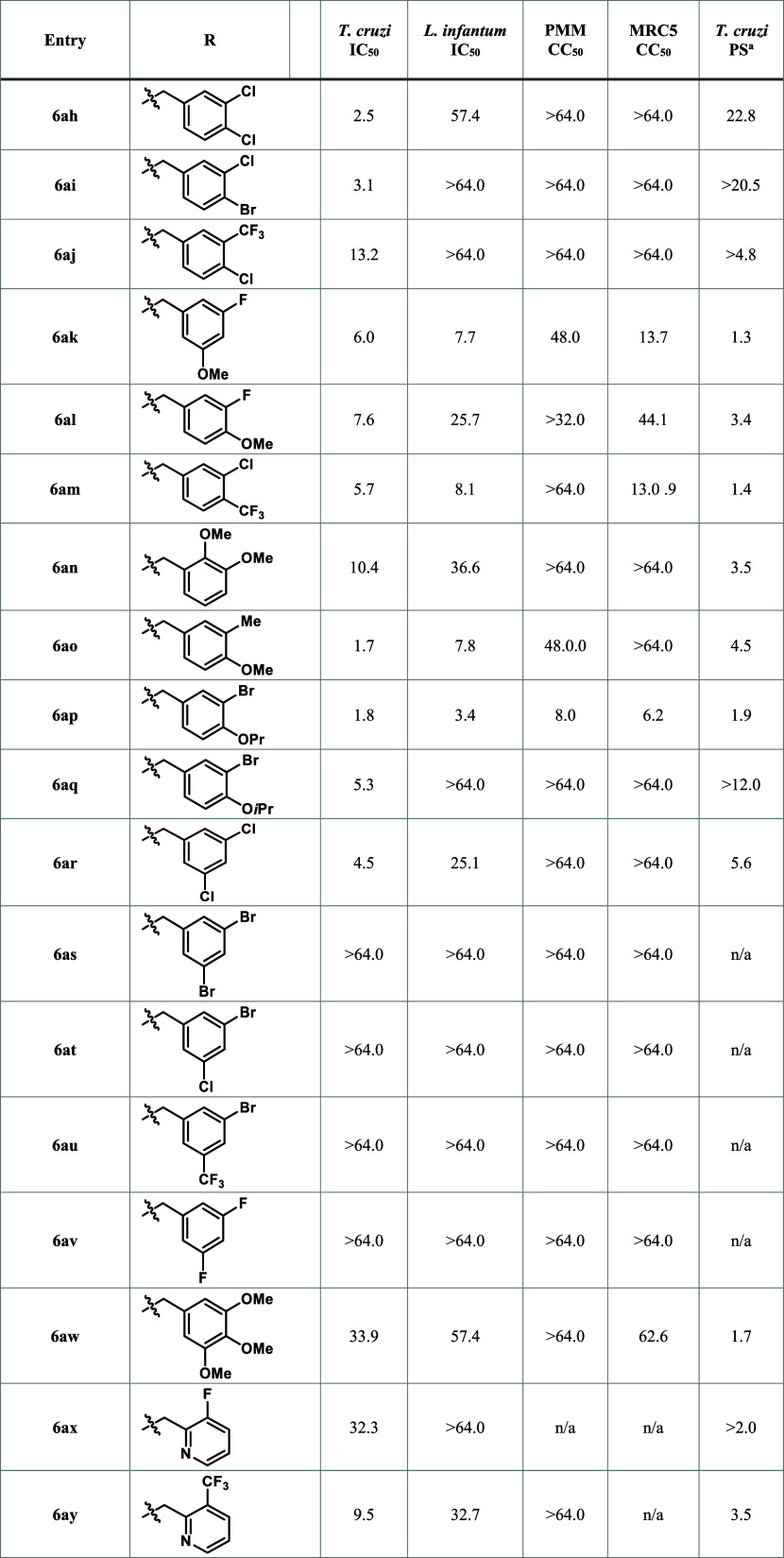

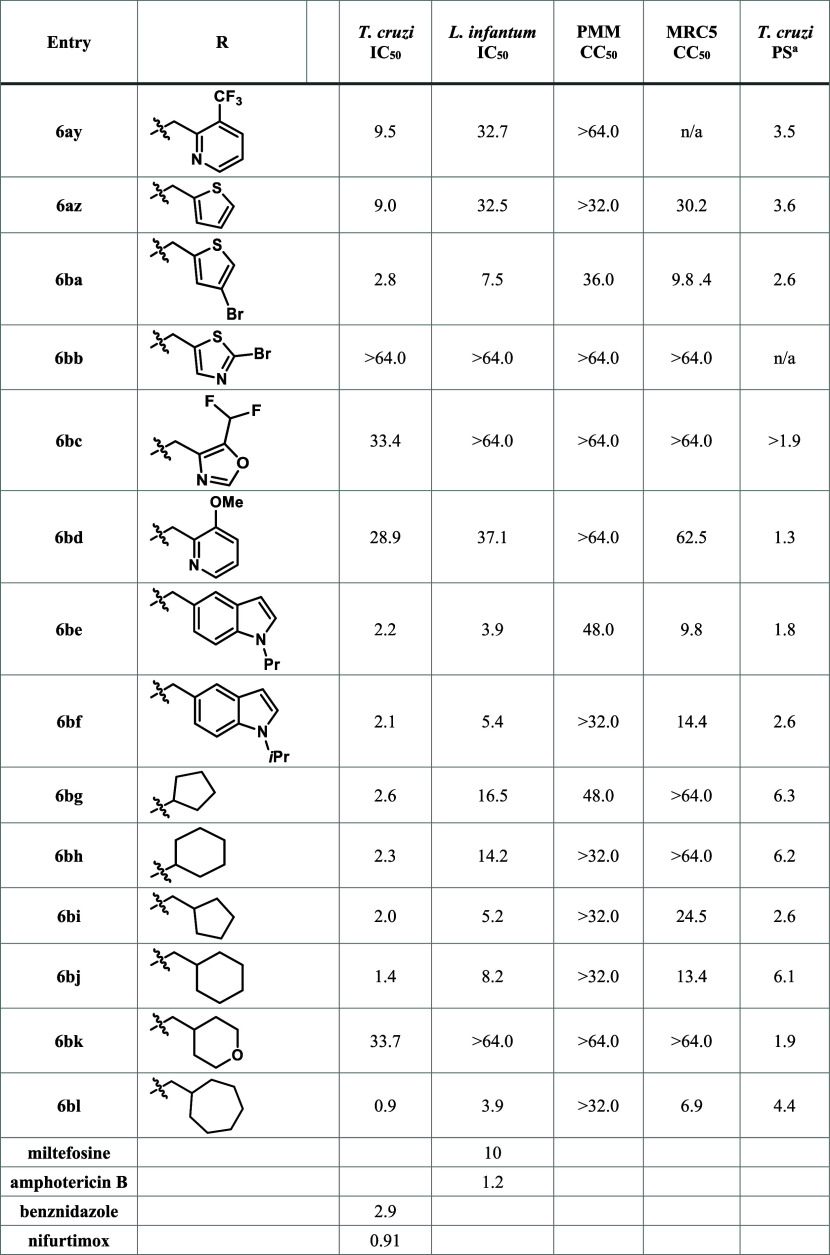
Antitrypanosomal Activity, Antileishmanial Activity, Cytotoxicity Assessment, and Selectivity Indices for *N*-Benzyl/Alkylaminocyclopropylpyrazole Analogues **6a**–**6bl** ^a^,^b^

a All parasitology and cytotoxicity data are shown as the average values in μM from two separate experiments. Individual replicates and SEM can be found in the Supporting Information.

b Parasite selectivity (PS) representing the ratio of *L. infantum* IC_50_/ *T. cruzi* IC_50_.

To establish preliminary SAR, methyl, fluoro, chloro, methoxy, nitro, trifluoro, and trifluoromethoxy analogues (**6b**–**6s**) were prepared in the ortho, meta, and para positions of the aryl ring (Table 1). The unsubstituted compound **6a** emerged as a moderately potent hit with an IC_50_ of 8.8 μM against *T. cruzi*, showing comparable potency against *L. infantum* and modest selectivity over PMM and MRC-5 cells, indicating target-derived activity over general cytotoxicity. Interestingly, with only one exception, substituting the aromatic ring generally improved or maintained the potency against *T. cruzi*. Additionally, the substitution pattern significantly influenced the selectivity for *T. cruzi* over *L. infantum*, varying from 0.7 to 50-fold. Each analogue in this initial SAR exploration demonstrated either improved or comparable potency against *T. cruzi* over *L. infantum*.

Adding a methyl group to any position of the ring (**6b**–**6d**) enhanced potency against *T. cruzi* up to 3.8-fold, while maintaining comparable potency against *L. infantum* *.* A similar increase in parasitic activity for *T. cruzi* was observed with fluorine substitution (**6g**–**6i)**, but chlorine substitution (**6e**–**6f**) was more successful, with the 4-chloro compound **6f** showing an 4.9-fold selectivity for *T. cruzi* over *L. infantum*. Methoxy substitution resulted in negligible change in potency compared to **6a**. Disubstitution with fluorine and methoxy groups (**6ak**–**6al**) provided compounds with modest potency and some improvement in the cytotoxicity window but negligible selectivity between the two parasites. Ortho-substitution with a nitro-group (**6m**) provided a compound with a comparable profile to **6a**, whereas para-substitution (**6n**) was inactive in the parasite and cytotoxicity assays. Trifluoromethyl and trifluoromethoxy containing compounds **6o**–**6s** provided a subseries of compounds with excellent selectivity for *T. cruzi* over *L. infantum* that are also inactive the cytotoxicity assays. Compound **6p** had the greatest selectivity for *T. cruzi*, 40-fold over *L. infantum*, and was also inactive in the cytotoxicity assays. 4-Trifluoromethoxy compound **6s** and 4-(trifluoromethyl)­thio-substituted compound **6w** had a similar profile to **6p** but slightly reduced *T. cruzi* parasite selectivity (PS). Addition of a pyridine nitrogen was detrimental to potency, as observed in fluorinated matched pairs **6g** to **6ax**.

Substitution with a pentafluorosulfanyl group in compounds **6t** and **6u** produced potent antitrypanosomal compounds with modest selectivity but increased toxicity compared to the trifluoromethyl compounds **6o** and **6p**. Overall, these simple substitutions to the aromatic ring were successful in providing confidence that changes to this ring could yield compounds with improved potency, although defining a clear preferred substitution pattern was not possible.

Based on these early observations, further investigation of the 3- and 4-positions of the aromatic ring yielded more compounds with improved potency against *T. cruzi* and varying degrees of selectivity toward *L. infantum*. Addition of a larger substituents in these positions, a second phenyl ring, generated biphenyl containing compounds **6x** and **6y** had similar profiles and comparable selectivity for *T. cruzi*. Similarly, larger ether compounds substituted with 3-benzyloxy **6ab**, 4-propoxy **6ad**, and 4-*iso*propoxy **6ae** had a more beneficial effect on potency c.f*.* methoxy substitution, with modest *T. cruzi* Supporting Information of between 2.0 and 2.7. Replacement of a phenyl group with 3,5-dimethylpyrazole, as in compound **6ag**, led to a modest drop in lipophilicity (cLogP^24^ **6x** = 4.54, **6ag** = 3.96) but also to a 4-fold reduction in potency and reduced parasite selectivity. 3,5-Disubstitution **6as**–**6av** was generally not tolerated, with poor potency measured in all the assays.

A small subset of heteroaromatic analogues was also prepared. The unsubstituted thiophene **6ba** had comparable potency (9.00 μM) to unsubstituted phenyl **6a**, but improved selectivity for *L. infantum*. Substituted thiophene **6bb** also had an improved PS for *T. cruzi* compared to 3-bromo analogue **6ac**, but was more cytotoxic. Thiazole **6bc** was inactive. Indole analogues **6bf**–**6bg** of the corresponding ethers had comparable potency but significant toxicity increases. Aliphatic analogues **6bh** to **6bm** showed very high potency against *T. cruzi*, with the cycloheptyl analogue **6bm** having a potency of 0.9 μM, and a significantly improved *F*sp^3^ (0.58). However, tetrahydropyran analogue **6bl** had a 35-fold reduction in potency of 33.7 μM. This significant reduction in potency with a bioisosteric CH_2_ to O exchange may indicate lipophilicity-driven potency within this subseries, as evidenced by the significant reduction in lipophilicity associated with this change (cLogP^24^ **6bj** = 3.68, **6bk** = 2.69).

The H-substituted series of pyrazoles **8a**–**8h** generally showed lower potency compared to their cyclopropyl counterparts (Table 2), with parasite selectivity indices ranging from 1.4 to 19.8. Pleasingly, the introduction of a biphenyl unit in compound **8g** matched the potency in *T. cruzi* as its cyclopropyl matched pair **6y** with a significantly reduced activity against *L. infantum*, providing a highly selective compound for *T. cruzi*.

**Table 2:**
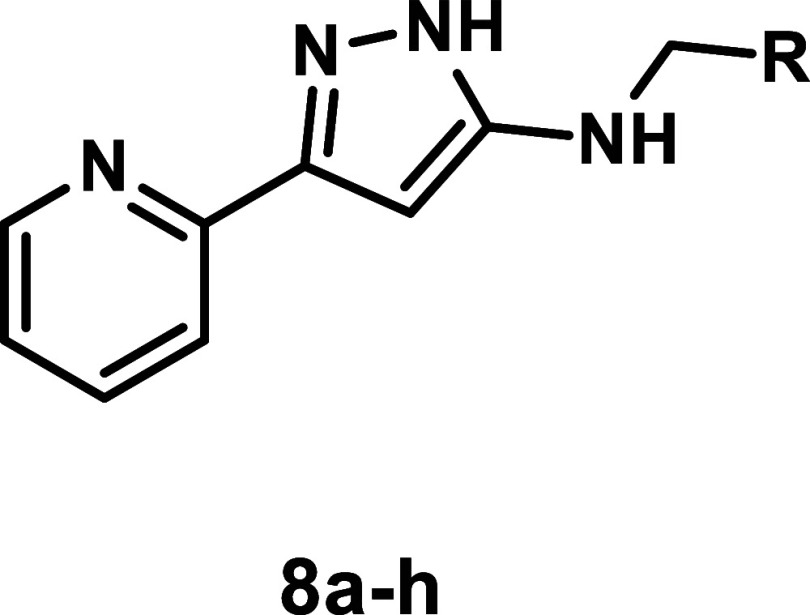

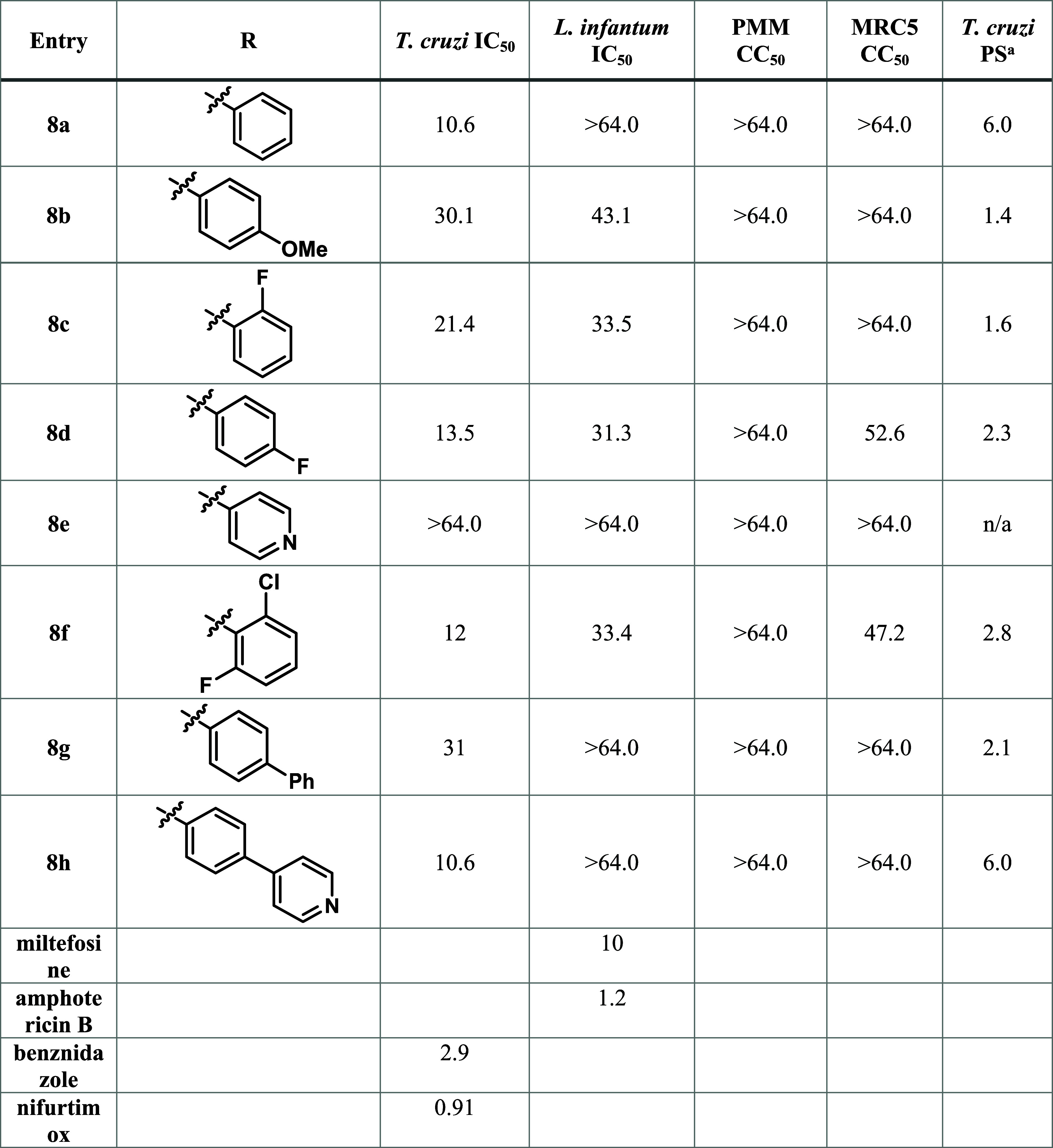
Antitrypanosomal Activity, Antileishmanial Activity, Cytotoxicity Assessment and Selectivity Indices for *N*-Benzylamino-*H*-Pyrazoles Analogues **8a**–**8h** ^a^

a All parasitology and cytotoxicity data are shown as the average values in μM from two separate experiments. Individual replicates and SEM can be found in the Supporting Information.

b Parasite selectivity (PS) represents the ratio of *L. infantum* IC_50_/*T. cruzi* IC_50_.

The analogues in this series generally showed no cytotoxicity in PMM or MRC5 cells.

Compounds **13** and **14** provided preliminary insight into the 2-pyridyl SAR and may be compared to compounds **6y** and **8g** as the matched pairs from the cyclopropyl and H-series (Table 3). Moving to a 3-pyridyl group (**13**) significantly increased the potency of the 4-biphenyl analogue compared to the 2-pyridyl **8g**, giving comparable potency to **6y**; **13** had improved selectivity over *L. infantum* and reduced cytotoxicity against PMM cells. The use of 2-fluorophenyl as a pyridyl isostere was ineffective, **14** was inactive against *T. cruzi*, but showed significant potency toward *L. infantum*, suggesting a critical interaction formed with the unknown *T. cruzi* target by a pyridyl nitrogen in either position.

**Table 3:**
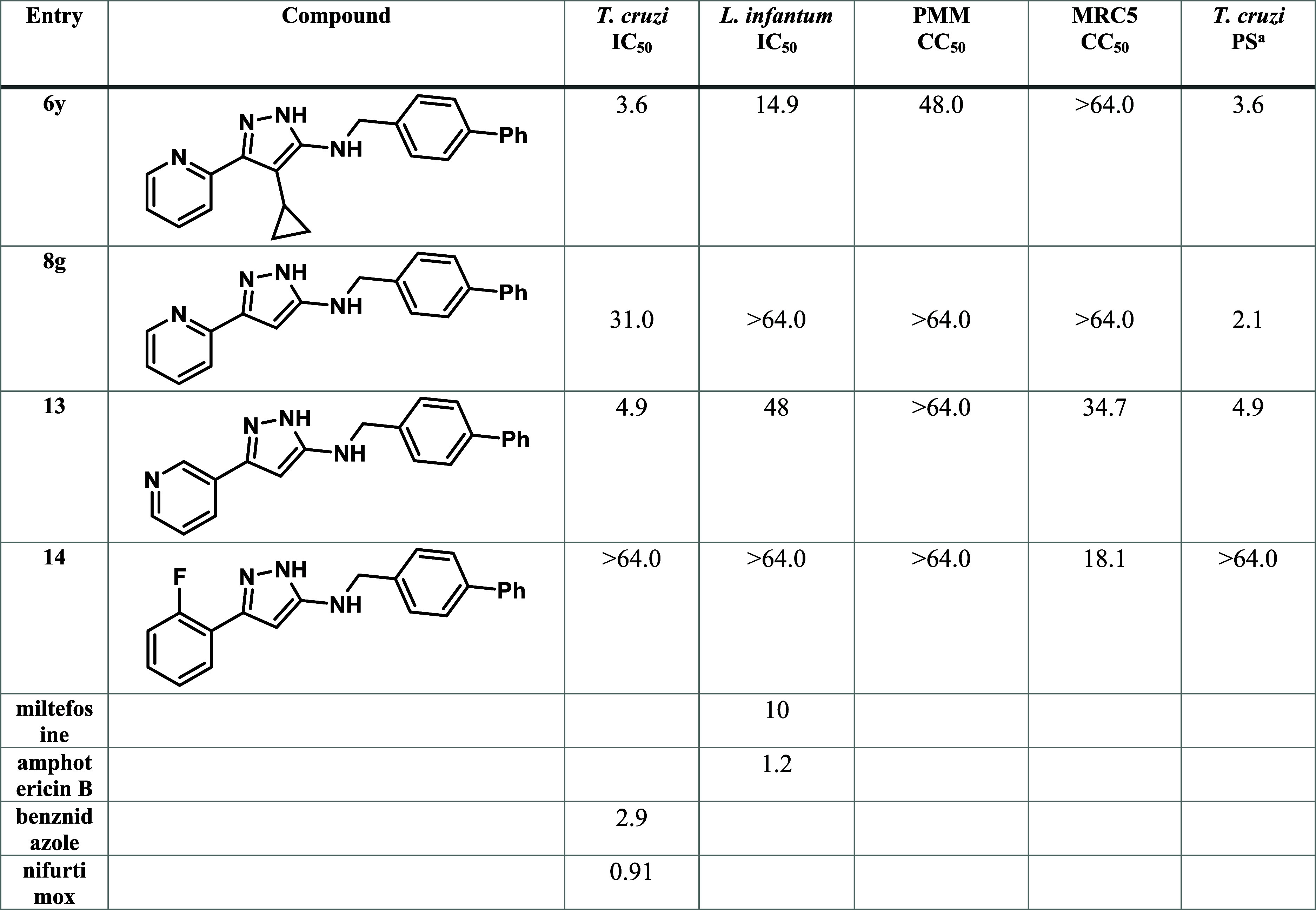
Antitrypanosomal Activity, Antileishmanial Activity, Cytotoxicity Assessment and Selectivity Indices for Pyridyl Isostere Analogues **6y, 8g, 13** and **14** ^a^

a All parasitology and cytotoxicity data are shown as the average values in μM from two separate experiments. Individual replicates and SEM can be found in the Supporting Information.

b Parasite selectivity (PS) representing the ratio of *L. infantum* IC_50_/ *T. cruzi* IC_50_.

### Physicochemical and Metabolic Evaluation

A set of analogues with good potency against *T. cruzi* and large cytotoxicity windows were investigated for solubility and stability in mouse liver microsomes (MLM) (Table 4). Disappointingly, the series showed very poor metabolic stability and low solubility at physiological pH. Each of the analogues tested possessed a very short half-life (*t*_1/2_ < 3 min) and was rapidly cleared. Using the Xenosite cytochrome P450 models, metabolism of the benzylic position (CYP2A6, CYP4A4) and cyclopropyl group (CYP2C8, CYP2E1) is predicted.^25^ This metabolic instability is greater than for the comparable amide and urea series assayed previously.^3^ The series’ poor metabolism and low solubility may be attributed to high lipophilicity and low *F*sp.^3^ Addressing these concerns is a key challenge for the future development of this series.

**Table 4:**
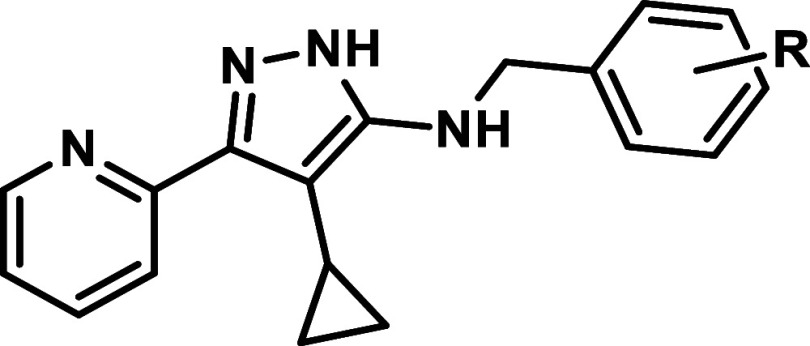
Structure, Antitrypanosomal Activity, Selectivity Indices, Mouse Liver Microsome (MLM), Solubility, cLogD, and *F*sp^3^ of Selected Analogues

entry	R	T. cruzi IC_50_ (μM)	MLM *t*_1/2_ (min)	MLM CL_int_ (μL/min/mg)	solubility, sifile1 (μM)	Solubility, pH = 7.4 (μM)	cLogD	*F*sp^3^
**6e**	3-Cl	2.1	<3.0	>577.5	199.6	4.0	4.15	0.16
**6x**	3-Ph	2.0	<3.0	>577.5	184.8	<2.5	5.19	0.13
**6y**	4-Ph	3.6	<3.0	>577.5	29.2	<2.5	5.19	0.13
**6ac**	3-Br	1.8	<3.0	>577.5	197.4	8.2	4.12	0.16
**6ai**	3-Cl, 4-Br	3.1	<3.0	>577.5	160.5	<2.5	4.92	0.16

## Conclusion

We have identified an *N*-alkylaminopyrazoles subseries from a published aminopyrazole amide series that gives compounds with preferred activity against *T. cruzi* over *L. infantum*. Early SAR investigation of this series, focusing on the benzyl ring, identified a series of potent compounds. Substitution is tolerated across the benzyl ring, leading to a range of compounds that have good *T. cruzi* potency and selectivity over *L. infantum*, and which were not cytotoxic in counter assays. Unfortunately, the most active compounds identified were poorly soluble at physiological pH and had poor microsomal stability, partly due to their high lipophilicity. A promising subset of potent alkyl-substituted pyrazoles (**6bu**, **6bx**) with improved *F*sp^3^ may indicate that the lipophilic group can be varied to a nonaryl group; further exploration of this SAR is ongoing.

Within a drug discovery program, medicinal chemists are required to design, synthesize, and test significant numbers of diverse compounds within a chemical series. This generates information about the chemical series, contributing to one of the core tenets of medicinal chemistry, the SAR. The empirical approach to medicinal chemistry design (“what happens if I make this change?”) lends itself particularly well to parallel, matrix-based synthetic chemistry in which multiple analogues around a particular series are exemplified via the same fundamental synthetic route using a diversity of key reagents or building blocks to explore the resulting SAR. This is particularly pertinent in the field of NTDs, where traditionally drug discovery projects are based on phenotypic campaigns. Most of DNDi’s discovery projects start as phenotypic projects, building on data sets from the screening of large libraries. This pairs well with academic teaching laboratories, where students can synthesize, in parallel, multiple variants around a particular structural motif by the simple variation of a key building block. As part of the OSN, an open initiative, we invite collaborators to participate in the further optimization of the series in the development of a preclinical candidate for CD.

## Methods

### Compound Synthesis

The synthesis of the compounds under investigation is reported in the Supporting Information.

### Biological Evaluation Assays

#### Compound Solutions/Dilutions

Compound stock solutions were prepared in 100% DMSO at 20 mM. The compounds were serially prediluted (2-fold or 4-fold) in DMSO, followed by a further (intermediate) dilution in demineralized water to ensure a final in-test DMSO concentration of <1%.

### Antileishmanial Activity

#### Parasite and Cell Cultures

The strain *Leishmania infantum* MHOM/MA­(BE)/67 was used. Parasites were maintained in golden hamsters (*Mesocricetus auratus*). Amastigotes were collected from the spleen of an infected donor hamster using three centrifugation purification steps (300 rpm, keeping the supernatants, 2200 rpm, keeping the supernatants and 3500 rpm, keeping the pellet), and spleen parasite burdens were assessed using the Stauber technique. Primary peritoneal mouse macrophages were used as host cells and were collected 2 days after peritoneal stimulation with a 2% potato starch suspension. All cultures and assays were conducted at 37 °C under an atmosphere of 5% CO_2_.

#### Drug Sensitivity Assays

Assays were performed in 96-well microtiter plates, each well containing 10 μL of the compound dilutions together with 190 μL of macrophage/parasite inoculum (3 × 10^4^ cells +4.5 × 10^5^ parasites/well). The inoculum was prepared in RPMI-1640 medium, supplemented with 2 mM l-glutamine, 16.5 mM NaHCO_3_, and 5% inactivated fetal calf serum. The macrophages were infected after 48 h. The compounds were added after 2 h of infection. Parasite multiplication was compared to untreated-infected controls (100% growth) and uninfected controls (0% growth). After 5 days of incubation, parasite burdens (mean number of amastigotes/macrophage) were microscopically assessed after staining the cells with a 10% Giemsa solution. The results are expressed as IC_50_ values.

#### Primary Screen

The compounds were tested at 5 or 10 concentrations depending upon the number of compounds (4-fold compound dilutions) (64–16–4–1–0.25–0.0625–0.015625–0.0039–0.000975 and 0.00024 μM). Amphotericin B and miltefosine were included as reference drugs. A test compound was classified as inactive when the IC_50_ was higher than 30 μM. When the IC_50_ was between 30 and 10 μM, the compound was regarded as moderately active. If the IC_50_ was lower than 10 μM, the compound was classified as highly active on the condition that it also demonstrated selective action (absence of cytotoxicity against primary peritoneal macrophages). A final recommendation for activity was given after a confirmatory evaluation in a secondary screen.

#### Secondary Screen

IC_50_ values were determined using an extended dose range (2-fold compound dilutions). Amphotericin B and miltefosine were included as reference drugs.

### Antitrypanosomal Activity

#### Parasite and Cell Cultures

*T. cruzi*, Tulahuen CL2, and β-galactosidase strain (nifurtimox-sensitive) were used.^26^ The strain was maintained on MRC-5SV2 (human lung fibroblast) cells in MEM medium, supplemented with 2 mM l-glutamine, 16.5 mM NaHCO_3_, and 5% inactivated fetal calf serum. All cultures and assays were conducted at 37 °C under an atmosphere of 5% CO_2_.

#### Drug Sensitivity Assays

Assays were performed in sterile 96-well microtiter plates, each well containing 10 μL of the aqueous compound dilutions together with 190 μL of MRC-5 cell/parasite inoculum (4 × 10^3^ cells/well +4 × 10^4^ parasites/well). Parasite growth was compared to untreated-infected controls (100% growth) and noninfected controls (0% growth) after 7 days of incubation at 37 °C and 5% CO_2_. Parasite burdens were assessed after adding 50 μL/well of a stock solution containing 15.2 mg of chlorophenol red β d-galactopyranoside +250 μL of Nonidet in 100 mL of phosphate buffered saline (PBS). The change in color was measured spectrophotometrically at 540 nm after 4 h incubation at 37 °C. The results were expressed as IC_50_ values.

#### Primary Screen

Compounds were tested at 5 or 10 concentrations depending upon the number of compounds (4-fold compound dilutions: 64–16–4–1–0.25–0.0625–0.015625–0.0039–0.000975 and 0.00024 μM or μg/mL). Nifurtimox or benznidazole was included as the reference drug. The test compound was classified as inactive when the IC_50_ was higher than 30 μM. When the IC_50_ was between 30 and 5 μM, the compound was regarded as moderately active. When the IC_50_ was lower than 5 μM, the compound was classified as highly active on the condition that it also demonstrated selective action (absence of cytotoxicity). A final recommendation for activity was given after confirmatory evaluation in a secondary screen.

#### Secondary Screen

IC_50_ values were determined using an extended dose range (2-fold compound dilutions). Nifurtimox and benznidazole were included as reference drugs.

### Toxicity against MRC-5 and PMM Cells

#### Cell Cultures

MRC-5_SV2_ cells were cultured in MEM + Earl’s salts-medium, supplemented with l-glutamine, NaHCO_3_ and 5% inactivated fetal calf serum. All cultures and assays were conducted at 37 °C under an atmosphere of 5% CO_2_.

#### Drug Sensitivity Assays

Assays were performed in sterile 96-well microtiter plates, each well containing 10 μL of the aqueous compound dilutions together with 190 μL of MRC-5_SV2_ inoculum (1.5 × 10^5^ cells/mL). Cell growth was compared to untreated control wells (100% cell growth) and medium control wells (0% cell growth). After 3 days of incubation, cell viability was assessed fluorometrically after addition of 50 μL of resazurin per well. After 4 h at 37 °C, fluorescence was measured (λ_ex_ 550 nm, λ_em_ 590 nm). The results were expressed as IC_50_ values.

#### Primary Screen

The compounds were tested at 5 or 10 concentrations depending upon the number of compounds (4-fold compound dilutions: 64–16–4–1–0.25–0.0625–0.015625–0.0039–0.000975 and 0.00024 μM or μg/mL). Tamoxifen or niclosamide were used as cytotoxic reference compounds.

#### Secondary Screen

IC_50_ values were determined using an extended dose range (2-fold compound dilutions) with a maximum concentration of 64 μM.

#### Kinetic Solubility Assay in PBS pH 7.4

The solubility assay was performed in a 96-well plate format. A solution of PBS and the test compound (200 μM) was incubated at 25 °C with constant shaking (600 rpm) for 2 h. The samples were filtered by using a multiscreen solubility filter plate. A five-point linearity curve was prepared in PBS: acetonitrile (1:1, v/v) at 200, 150, 75, 25, and 2.5 μM. Blank, linearity, and test samples (*n* = 2) were transferred to a UV readable plate, and the plate was scanned for absorbance. Best fit calibration curves were constructed using the calibration standards and used to determine the test sample solubility. The experiment was carried out in duplicate. Diethylstilbestrol, haloperidol, and sodium diclofenac were used as reference standards.

#### Metabolic Stability Study Using Liver Microsomes

A solution of the test compounds in phosphate buffer solution (1 μM) was incubated in pooled human liver microsomes (0.4 mg/mL) for 0, 5, 20, 30, 45, and 60 min at 37 °C in the presence and absence of an NADPH regeneration system (NRS). The reaction was terminated with the addition of an ice-cold acetonitrile-containing system suitability standard at designated time points. The sample was centrifuged (3500 rpm) for 20 min at 15 °C, and the supernatant was half diluted in water and then analyzed by means of LC–MS/MS. The percentage of the remaining parent compound, half-life (*t*_1/2_), and clearance (CL_int,app_) were calculated using standard methodology. The experiment was carried out in duplicate. Atenolol, propranolol, diclofenac, and verapamil were used as reference standards.

## Supplementary Material







## Notes

Molecular formula strings with associated data (XLSX)
Full description of in vitro protocols, synthesis of intermediates and additional compounds, and NMR and HPLC purity profile (PDF)
(PDF)

## Abbreviations

CD: Chagas disease
cLogD: calculated logarithm of distribution coefficient
CPRG: chlorophenol red β-d-galactopyranoside
CYP: cytochrome P450
DNDi: Drugs for Neglected Diseases Initiative
Fsp^3^: fraction of sp3-carbon atoms
HAT: human African Trypanosomiasis
HTS: high-throughput screening
MLM: mouse liver microsomes
MRC-5: human fetal lung fibroblasts
NTDs: Neglected tropical diseases
OSN: Open Synthesis Network
PMM: peritoneal mouse macrophages
R&D: research and development
SAR: structure–activity relationships
SI: selectivity index
WHO: World Health Organisation.
