# Supplementary material for: Collaborative Synthesis for Neglected Diseases through the Open Synthesis Network: Structure–Activity Relationships of Arylaminopyrazoles as Chagas Disease Treatments
Source: ACS Infect Dis. 2025 Aug 11;11(9):2593–606. doi: 10.1021/acsinfecdis.5c00481 (PMC12442103; doi:10.1021/acsinfecdis.5c00481)
Supplement: Supplementary file 2 [file id5c00481_si_002.pdf]

SUPPORTING INFORMATION

Collaborative Synthesis for Neglected Diseases through the Open Synthesis Network: Structure-Activity-Relationships of Arylaminopyrazoles as Chagas Disease Treatments

Zigli Abdulai,[f] Natasha Agbo,[f] Jonathan I Anderson,[b] Faye Astley,[b] Brian Chan,[e] Owen Atkinson-Evans,[b] João L Avelar,[d] Cristiane Aparecida-Silva,[d] KD Bhardwaj,[b] William J Bowley,[b] Nicholas Breitkreuz,[b] Rosie Canby,[b] Emerald Cartwright,[b] Charles Clifford,[b] Shannon A Cordell,[b] William J Donker,[b] Joseph Driscoll,[b] Matthew Grady,[b] Lauren Higginbotham,[b] Devon Hsu,[e] Jamie Hutchinson,[b] Lukas Imberg,[g] Harry F Jackson,[b] Finan Johns,[b] Emma Jones,[b] Dmitrii V. Kalinin,[g] Ceren Kardeșler,[b] Aaron Keal,[b] Libby Keel,[b] Sumin Kim,[e] Phoebe Knight,[b] Justus F. Ködel,[h] Luke Kumeta,[b] Hyejin Lee,[e] Sam Le Roy,[b] Rachelle Maccarone,[e] Maria Mahmud,[b] Matthew Martin,[b] Ignatius Nguyen,[e] Conor J Nolan,[b] Lucy Noyes,[b] Angela NW Ntuwa,[b] Wiktoria Obarska,[b] Owen Oldham,[b] Edna Onyiuke,[e] Jess Otter,[b] Hugh Page,[b] Dhruvi Patel,[e] Kayla Reid,[e] Krishna Samaddar,[b] Sheryar M Shabbir,[b] Peter Shevlin,[e] Callum Sinclair-Wright,[b] Amanda

Smithies,[b] Amelia S Thomson,[b] Jack Tinker,[b] Alexander Uner,[b] Natascha Van Pelt,[h] Ewan Waddell,[b] Hamis Wagwa,[e] Claire Walthorne,[b] Sophie Warner,[b] Tobias Winge,[g] Ngai Sum Wong,[b] Leon Jan Wysocki,[b] Callum A Yong,[b] Zaria Zaheen,[e] An Matheeussen,[i] Guy Caljon,[i] Richard Amewu,[f] Anna Bertram,[b] Bernhard Biersack,[h] Carolyn Friel,[e] Lídia Moreira Lima,[d] Chase Smith,[e] Bernhard Wünsch,[g] Benjamin Perry,[c] Luiza R. Cruz,[j] and Andrew Nortcliffe*,[ab]

[a]GlaxoSmithKline Carbon Neutral Laboratories for Sustainable Chemistry, School of Chemistry, University of Nottingham, Triumph Road, Nottingham, NG7 2TU, United Kingdom

[b]School of Chemistry, University of Nottingham, University Park, Nottingham, NG7 2RD, United Kingdom

[c]Drugs for Neglected Diseases initiative, 15 Chemin Camille Vidart, Geneva 1202, Switzerland

[d]Laboratório de Avaliação e Síntese de Substâncias Bioativas, Centro de Ciências da Saúde, Universidade Federal do Rio de Janeiro, Cidade Universitária, Rio de Janeiro, RJ, Brazil

[e]Department of Pharmaceutical Sciences, School of Pharmacy-Worcester/Manchester, Massachusetts College of Pharmacy and Health Sciences, 19 Foster Street, Worcester, MA 01608, United States of America.

[f]Department of Chemistry, University of Ghana, P.O. Box LG56, Legon, Accra, Ghana

[g]Institut für Pharmazeutische und Medizinische Chemie der Westfälischen Wilhelms-Universität Münster, Correnssstraße 48, D-48149 Münster, Germany

[h]Organic Chemistry Laboratory, University of Bayreuth, Universitätsstrasse 30, 95440 Bayreuth, Germany

[i]Laboratory of Microbiology, Parasitology and Hygiene (LMPH), University of Antwerp, Universiteitsplein 1, B-2610 Wilrijk, Belgium

[j]Drugs for Neglected Diseases initiative, Rio de Janeiro, 20010020, Brazil.

Corresponding author email: andrew.nortcliffe@nottingham.ac.uk

Contents

S3 General Experimental information

S6-S7 General procedure for reductive amination for cyclopropyl pyrazole compounds and General procedure for reductive amination for *H*-pyrazole compounds.

S8-S50 Characterisation data.

General experimental information

Reactions were carried out under an atmosphere of nitrogen, unless otherwise noted. Commercially available reagents were used throughout, without purification unless otherwise stated. Petroleum ether refers to the fraction with bp 40-60 °C. Ether refers to diethyl ether. All aqueous solutions were prepared using deionised water. The following solvents were dried for moisture sensitive reactions: THF (distillation from sodium and benzophenone under N_2), CH_2Cl_2 (distillation from $CaCl_2$ under N_2), DMF, toluene, ether, pyridine (stored over 3/4 Å MS)

Chemicals were purchased from: Sigma Aldrich, Alfa Aesar, Chempur, Fisher Scientific, Fluorochem, Manchester Organics or TCI.

Analytical thin-layer chromatography was carried out on aluminium-backed plates coated with Merck Kieselgel 60 GF254 or Merck Kieselgel 60 NH2 F_{254}S.

NMR spectra were recorded at 298 K using Bruker AV(III)400, AV400 (400 MHz ^{1}H frequency, 101 MHz ^{13}C frequency, Nottingham), Bruker AV(III)300, AV300 (300 MHz ^{1}H frequency, 101 MHz ^{13}C frequency, Bayreuth), Bruker DRX 400 (400 MHz ^{1}H frequency, 101 MHz ^{13}C frequency, Rio de Janeiro), Agilent DD2 400 (400 MHz ^{1}H frequency, 101 MHz ^{13}C frequency, Münster) Bruker AV(III)500 AV500 (500 MHz ^{1}H frequency, 126 MHz ^{13}C frequency, Worcester or Nottingham equipped with a cryoprobe) or Bruker DRX 500 (500 MHz ^{1}H frequency, 126 MHz ^{13}C frequency, Rio de Janeiro) or Agilent DD2 600 (600 MHz ^{1}H frequency, 126 MHz ^{13}C frequency, Münster equipped with a cryoprobe). Chemical shifts are

quoted in parts per million (ppm), referenced to residual chloroform (7.26 ppm for ^{1}H NMR, 77.16 ppm for ^{13}C NMR), and methanol (3.31 ppm for 1H NMR, 49.00 ppm for ^{13}C NMR) as internal standards and coupling constants, *J*, are quoted in Hz. Multiplicities are as follows: s – singlet, br s – broad singlet, m – multiplet, d – doublet, dd – doublet of doublets, ddd – doublet of doublet of doublets, dt – doublet of triplets, t – triplet, td – triplet of doublets, tt – triplet of triplets, q – quartet, qd – quartet of doublets, p – pentet (quintet).

Under reduced pressure refers to the use of a Vaccubrand CVC 3000 vacuum pump to remove solvent under reduced pressure on a Büchi Rotavapor R-3000 or Heidolph Vei-Vap Value G3 apparatus, with a water bath at 40 ºC.

TLC plates were visualised under UV light (254 or 365 nm) and/ or stained with the appropriate staining solution. The staining solution, basic aqueous potassium permanganate ($KMnO_4$), is reported when used.

Flash column chromatography was carried out using Interchim Puriflash pre-packed silica gel, eluting with the aid of an Asynt chromatography pump or Biotage SP4.

Melting points were measured on a Stuart SMP20 digital melting point apparatus (Nottingham), Mettler Toledo MP50 (Münster) or Gallenkamp (Bayreuth) apparatus or by Differential Scanning Calorimetry using a Shimadzu DSC-60 calibrated with indium (Rio de Janeiro); and are reported to the nearest degree, uncorrected

Mass Spectrometric analyses at the School of Chemistry, GlaxoSmithKline Carbon Neutral Laboratories, University of Nottingham were recorded on a Bruker MicroTOF 61 mass spectrometer using electrospray ionization (ESI) or at Boston University Chemistry Department Chemical Instrumentation Center, Boston, MA, USA recorded on an Agilent LC/MSD VL mass spectrometer using electrospray ionization or at LASSBio, Institute of Biomedical Sciences, Federal University of Rio de Janeiro, Brazil recorded on a QExactive Plus High Relution Mass Spectrometer (Thermo Scientific) using electrospray ionization, University of Bayreuth on UPLC/Orbitrap (Bayreuth). Atmospheric pressure chemical ionization (APCI) mass spectra were recorded with a MicroTOFQ mass spectrometer (Bruker) (Münster),. Data were analyzed with DataAnalysis. The DirectProbe/APCI-source was operated in the positive ionization mode, scan range 60 - 1000 *m/z*. APCI-conditions: The capillary was set to 4.0 kV, the nebulizer was operated at 0.7 bar, the dry gas was set to 3.0 L/min at a temperature of 200 °C. Mass calibration was done using a Fatty Acid Methyl Esters (FAME) solution (*m/z* 103 - 383 or 423 - 565) in dichloromethane. *m/z* values are reported in Daltons.

Liquid chromatography-mass spectrometry (LCMS) and High performance liquid chromatography (HPLC) analyses were performed using two methods. Method A: Agilent 1260 Infinity HPLC with a 6120 Quadrupole mass spectrometer. Chromatography conditions: High pH - Waters XBridge C18 3.5µm 2.1 x 30 mm column. Mobile phase A: 0.1% Ammonia in water, mobile phase B: acetonitrile. Flow rate 0.8 mL/min in a gradient of 5–95 % mobile phase B over 3.5 minutes with UV detection at 210 - 400 nm reported at 254nm. Column temperature 40 °C. Method B: Agilent LC/MSD VL system by electrospray (ESI) in the [positive or negative] mode with a reverse-phase C18 Zorbax Eclipse 2.1 x 50 mm column (Agilent).

Mobile phases were water and acetonitrile with 0.1% formic acid. Separation was achieved by a flow rate of 0.15 mL/min and a mobile phase gradient from 5 to 95% acetonitrile in 10 minutes. Method C: Dionex Ultimate 3000; UV detector: VDW-3400 RS; autosampler: WPS-3000 PL; pump: LPG-3600; column: LiChrospher® 60 RP –select B (5 µm), 250-4 mm cartridge. Method:flow rate: 1.00 mL/min; injection volume: 5.0 L; detection: λ = 210 nm; solvents: A: water with 0.05 % (*v*/*v*) trifluoroacetic acid, B: acetonitrile with 0.05 % (*v*/*v*) trifluoroacetic acid; gradient elution: (A %) 0-4 min: 90 %, 4-29 min: gradient from 90 % to 0 %, 29-31 min: 0 %, 31-31.5 min: gradient from 0 % to 90 %, 31.5-40 min: 90 %.

Infrared spectra were recorded using a Bruker Alpha Platinum ATR single reflection diamond module spectrometer over the range of 4000–600 cm^{-1} (Nottingham), using a Shimadzu IRTracer-100 over the range of 4000–600 cm^{-1} (Rio de Janeiro), MIRacle 10 (Shimadzu) using the IRsolution software over the range of 4000–600 cm^{-1} (Münster) or a Perkin-Elmer Spectrum One FT-IR spectrophotometer with ATR sampling unit over the range of 4000–600 cm^{-1} (Bayreuth).

Miscellaneous: Brine refers to aqueous saturated solution of sodium chloride.

General procedures for reductive amination for cyclopropyl pyrazole compounds.

Method A: To a solution of 4-cyclopropyl-3-(pyridin-2-yl)-1*H*-pyrazol-5-amine (200 mg, 1.00 mmol) in 1,2-dichloromethane (anhydrous, 5 mL) was added the appropriate aldehyde (1.2 mmol), acetic acid (57 µL, 1 mmol) and 3Å molecular sieves (200 mg, 100% *w*/*w*). After 30 minutes, sodium triacetoxyborohydride (636 mg, 3.00 mmol) was added. The reaction mixture was stirred overnight at room temperature. Following this time, aqueous sodium hydrogen carbonate solution (saturated, 50 mL) was added and the reaction mixture extracted with ethyl acetate (2 × 50 mL). The combined organic layers were washed with brine (50 mL), dried over Na_2SO_4, filtered and the solvent removed *in vacuo*. The residue was diluted with dichloromethane (5 mL) and adsorbed onto silica gel. Purification by flash column chromatography (silica gel), eluting with ethyl acetate and cyclohexane (2:8 to 10:0) gave the *title compound*.

OR

Method B: To a solution of 4-cyclopropyl-3-(pyridin-2-yl)-1*H*-pyrazol-5-amine (1 equiv.) in dichloromethane (anhydrous, 5 mL) the reaction mixture was stirred under reflux for 4 h. The precipitated imine was collected and suspended in methanol or ethanol (5 mL) and sodium borohydride (2 equiv.) was added. The reaction mixture was heated with a heat gun to dissolve the solids and stirred at room temperature for 1 h. Following this time, the reaction mixture was diluted with water and basified to pH = 9 with sodium hydroxide solution (2 M). The solution was extracted with dichloromethane and the organic layer washed with water (15 mL). The combined organic layers were dried over $MgSO_4$, filtered and the solvent removed *in vacuo*. The residue was purified by flash column chromatography (silica gel), eluting with ethyl acetate and cyclohexane (1:1) *to* give the *title compound*.

General procedure for reductive amination for *H*-pyrazole compounds.

To a solution of the appropriate aldehyde (6.68 mmol) in tetrahydrofuran (anhydrous, 15 mL) was added HCl (1 drop) and the reaction mixture stirred for 3 minutes. Following this time, 3-(pyridin-2-yl)-1*H*-pyrazol-5-amine (107 mg, 0.67 mmol) was added and reaction mixture stirred until the imine precipitated. Following this time, sodium borohydride (75 mg, 1.98 mmol) was added, and the reaction mixture stirred at room temperature for 16 h. The reaction mixture was alkalinised to pH 12 and extracted with ethyl acetate (2 × 50 mL). The combined organic layers were washed with brine (50 mL), dried over Na_2SO_4, filtered and the solvent removed *in vacuo.* Purification by flash column chromatography (silica gel), eluting with ethyl acetate and cyclohexane (7:3) gave the *title compound*.

***N*-Benzyl-4-cyclopropyl-3-(pyridin-2-yl)-1*H*-pyrazol-5-amine, 6a**

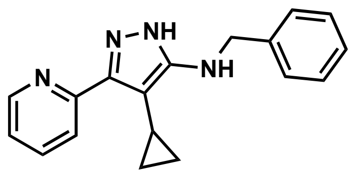

Isolated as a colourless solid (104 mg, 0.36 mmol, 36%); **mp.** 118 – 123 ºC; $\mathbf{R_f}$ 0.36 (1:1 EtOAc:*c*Hex); **FT-IR** ν_{max} (ATR)/cm^{-1} 3391, 3135, 3077, 3059, 3023, 2993, 2973, 2930, 2892, 2861, 1594, 1567, 1520, 1500, 1480, 1466, 1452, 1432, 1396; **^{1}H NMR** (400 MHz; $CDCl_3$) δ 10.22 (1 H, s), 8.59 (1 H, ddd, *J* 4.8, 1.9, 0.9), 7.75 (1 H, td, *J* 7.7, 1.8), 7.47 – 7.40 (2 H, m), 7.35 (2 H, tt, *J* 8.2, 1.6), 7.31 – 7.25 (2 H, m), 7.21 (1 H, ddd, *J* 7.5, 4.8, 1.2), 4.54 (2 H, s), 3.99 (1 H, s), 1.64 (1 H, tt, *J* 8.0, 5.3), 1.07 – 0.84 (2 H, m), 0.63 – 0.41 (2 H, m); **^{13}C NMR** (100 MHz; $CDCl_3$) δ 157.7, 149.5, 148.4, 140.6, 140.0, 136.6, 128.7, 127.9, 127.9, 127.2, 122.5, 121.5, 104.3, 48.6, 7.3, 3.8; **HRMS** *m*/*z* (ESI$^+$) calcd. for $C_{18}H_{18}N_4Na$ [M+Na]$^+$ requires 313.1424, found 313.1418; **LCMS** 2.75 min, 100%, *m/z* 291 [M+H]$^+$ (Method A).

4-Cyclopropyl-*N*-(2-methylbenzyl)-3-(pyridine-2-yl)-1*H*-pyrazol-5-amine, 6b

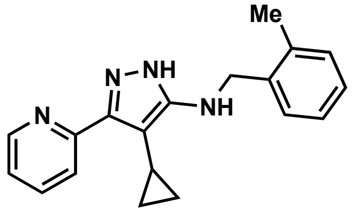

Isolated as a yellow solid (177 mg, 0.58 mmol, 58%); **mp.** 144 – 145 ºC; **R_f** 0.21 (1:1 EtOAc:*c*Hex); **FT-IR** ν_{max} (ATR)/cm^{-1} 3216, 1591, 1496, 1139, 1032, 998, 892, 825, 743; **^{1}H NMR** (400 MHz; $CDCl_3$) δ 10.22 (1 H, s), 8.59 (1 H, ddd, *J* 4.9, 1.9, 0.9), 8.00 (1 H, dt, *J* 8.0, 1.1), 7.76 (1 H, td, *J* 7.7, 1.8), 7.41 (1 H, td, *J* 4.0, 2.5), 7.25 – 7.16 (4 H, m), 4.63 – 4.43 (2 H, m), 3.82 (1 H, s), 2.42 (3 H, s), 1.63 (1 H, tt, *J* 8.0, 5.3), 1.04 – 0.92 (2 H, m), 0.57 – 0.46 (2 H, m); **^{13}C NMR** (100 MHz; $CDCl_3$) δ 157.8, 149.5, 148.4, 140.0, 138.1, 136.8, 136.5, 130.5, 128.5, 127.4, 126.2, 122.5, 121.5, 104.2, 46.6, 19.2, 7.3, 3.8; **HRMS** *m*/*z* (ESI$^+$) calcd. for $C_{19}H_{21}N_4$ [M+H]$^+$ requires 305.1761, found 305.1763; **LCMS** 2.83 min, 95%, *m/z* 305 [M+H]$^+$ (Method A).

4-Cyclopropyl-*N*-(3-methylbenzyl)-3-(pyridine-2-yl)-1*H*-pyrazol-5-amine, 6c

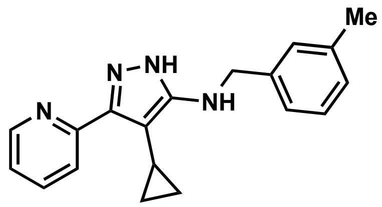

Isolated as a colourless solid (190 mg, 0.62 mmol, 62%); **mp.** 104 – 105 ºC; **R_f** 0.22 (1:1 EtOAc:*c*Hex); **FT-IR** ν_{max} (ATR)/cm^{-1} 3247, 1591, 1513, 1495, 1427, 1294, 1139, 999, 895; **^{1}H NMR** (400 MHz; $CDCl_3$) δ 10.33 (1 H, s), 8.59 (1 H, ddd, *J* 4.8, 1.9, 0.9), 8.00 (1 H, dt, *J* 8.0, 1.1), 7.75 (1 H, td, *J* 7.7, 1.8), 7.31 – 7.16 (4 H, m), 7.14 – 7.04 (1 H, m), 4.51 (2 H, s), 3.97 (1 H, s), 2.36 (3 H, s), 1.64 (1 H, tt, *J* 8.0, 5.3), 1.05 – 0.87 (2 H, m), 0.61 – 0.35 (2 H, m); **^{13}C NMR** (100 MHz; $CDCl_3$) δ 157.7, 149.4, 148.4, 140.5, 139.9, 138.3, 136.5, 128.8, 128.6, 127.9, 124.9, 122.5, 121.5, 104.3, 48.6, 29.8, 21.6, 7.3, 3.9; **HRMS** *m*/*z* (ESI$^+$) calcd. for

$C_{19}H_{21}N_4$ [M+H]$^+$ requires 305.1761, found 305.1754; **LCMS** 2.83 min, 95%, *m/z* 305 [M+H]$^+$ (Method A).

4-Cyclopropyl-*N*-(4-methylbenzyl)-3-(pyridine-2-yl)-1*H*-pyrazol-5-amine, 6d

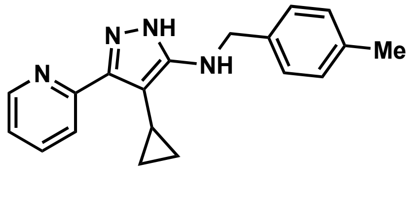

Isolated as a colourless solid (159 mg, 0.52 mmol, 52%); **mp.** 172 – 175 ºC; R_f 0.26 (1:1 EtOAc:*c*Hex); **FT-IR** ν_{max} (ATR)/cm^{-1} 3129. 1685, 1595, 1568, 1515, 1469, 1433. 1109; ^{1}**H NMR** (400 MHz; $CDCl_3$) δ 10.31 (1 H, s), 8.59 (1 H, ddd, *J* 4.8, 1.9, 1.0), 7.99 (1 H, dt, *J* 8.0, 1.1), 7.75 (1 H, td, *J* 7.7, 1.8), 7.38 – 7.29 (2 H, m), 7.21 (1 H, ddd, *J* 7.7, 4.9, 1.3), 7.18 – 7.10 (2 H, m), 4.50 (2 H, s), 3.94 (1 H, s), 2.35 (3 H, s), 1.63 (1 H, tt, *J* 7.9, 5.3), 1.07 – 0.89 (2 H, m), 0.63 – 0.39 (2 H, m); ^{13}**C NMR** (100 MHz; $CDCl_3$) δ 157.7, 149.4, 148.4, 139.9, 137.5, 136.8, 136.5, 129.3, 128.0, 127.9, 127.9, 122.5, 121.5, 104.3, 48.4, 21.3, 7.3, 3.8; **HRMS** *m*/*z* (ESI$^+$) calcd. for $C_{19}H_{20}N_4Na$ [M+Na]$^+$ requires 327.1580, found 327.1576; **LCMS** 2.87 min, 100%, *m/z* 305 [M+H]$^+$ (Method A).

4-Cyclopropyl-*N*-(3-chlorobenzyl)-3-(pyridine-2-yl)-1*H*-pyrazol-5-amine, 6e

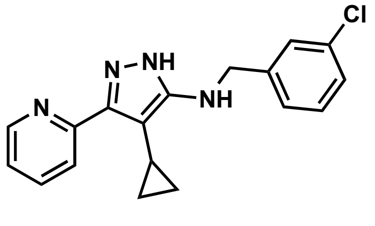

Isolated as a yellow solid (188 mg, 0.58 mmol, 58%); **mp.** 136 – 138 ºC; **R$_f$** 0.23 (1:1 EtOAc:*c*Hex); **FT-IR** ν_{max} (ATR)/cm^{-1} 3361, 1589, 1519, 1473, 1422, 1143, 781, 679, 551; **^{1}H NMR** (400 MHz; $CDCl_3$) δ 10.31 (1 H, s), 8.59 (1 H, ddd, *J* 4.8, 1.9, 1.0), 8.00 (1 H, dt, *J* 7.9, 1.1), 7.76 (1 H, td, *J* 7.8, 1.9), 7.46 – 7.40 (1 H, m), 7.34 – 7.18 (4 H, m), 4.63 – 4.41 (2 H, m), 4.03 (1 H, s), 1.65 (2 H, tt, *J* 8.0, 5.3), 1.12 – 0.90 (2 H, m), 0.68 – 0.37 (2 H, m); **^{13}C NMR** (100 MHz; $CDCl_3$) δ 157.3, 149.5, 148.3, 142.8, 140.0, 136.6, 134.4, 129.9, 127.9, 127.3, 125.9, 125.9, 122.6, 121.5, 104.3, 47.9, 7.3, 3.8; **HRMS** *m*/*z* (ESI$^+$) calcd. for $C_{18}H_{18}{}^{35}ClN_4$ [M+H]$^+$ requires 325.1215, found 325.1213; **LCMS** 2.86 min, 95%, *m/z* 325 [M+H]$^+$ (Method A).

4-Cyclopropyl-*N*-(4-chlorobenzyl)-3-(pyridine-2-yl)-1*H*-pyrazol-5-amine, 6f

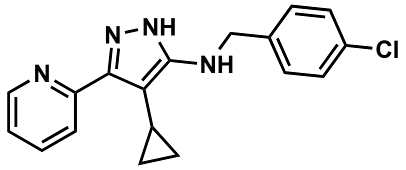

Isolated as a colourless solid (174 mg, 0.54 mmol, 54%); **mp.** 163 – 167 ºC; **R$_f$** 0.25 (1:1 EtOAc:*c*Hex); **FT-IR** ν_{max} (ATR)/cm^{-1} 3385, 3235, 3077, 3055, 3001, 2960, 2925, 2898, 2868, 1907, 1589, 1568, 1520, 1489, 1443; **^{1}H NMR** (400 MHz; $CDCl_3$) δ 10.36 (1 H, s), 8.59 (1 H, tt, *J* 2.8, 1.8), 7.99 (1 H, dt, *J* 8.0, 1.3), 7.75 (1 H, td, *J* 7.7, 1.9), 7.39 – 7.33 (2 H, m), 7.33 – 7.27 (2 H, m), 7.21 (1 H, ddt, *J* 7.5, 4.9, 1.3), 4.51 (2 H, d, *J* 1.9), 4.00 (1 H, s), 1.78 – 1.55 (1 H, m), 0.98 (2 H, dtd, *J* 8.2, 4.0, 1.9), 0.51 (2 H, dt, *J* 5.4, 1.8); **^{13}C NMR** (100 MHz; $CDCl_3$) δ 157.4, 149.5, 148.3, 140.0, 139.2, 136.6, 132.8, 129.2, 129.2, 128.7, 122.6, 121.5, 121.5, 104.3, 47.8, 7.3, 3.8; **HRMS** *m*/*z* (ESI$^+$) calcd. for $C_{18}H_{18}ClN_4$ [M+H]$^+$ requires 325.1215, found 325.1212; **LCMS** 2.91 min, 100%, *m/z* 325 [M+H]$^+$ (Method A).

4-Cyclopropyl-*N*-(2-fluorobenzyl)-3-(pyridine-2-yl)-1*H*-pyrazol-5-amine, 6g

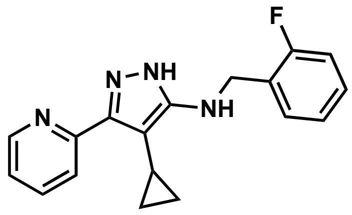

Isolated as a colourless solid (185 mg, 0.60 mmol, 60%); **mp.** 137 – 139 ºC; $\mathbf{R_f}$ 0.18 (1:1 EtOAc:*c*Hex); **FT-IR** ν_{max} (ATR)/cm^{-1} 3218, 1591, 1489, 1428, 1218, 1146, 1101, 996, 787; ^{1}**H NMR** (400 MHz; $CDCl_3$) δ 10.23 (1 H, s), 8.58 (1 H, ddd, *J* 4.9, 1.9, 1.0), 7.98 (1 H, dt, *J* 8.0, 1.1), 7.75 (1 H, td, *J* 7.7, 1.8), 7.47 (1 H, td, *J* 7.5, 1.9), 7.22 (2 H, dddd, *J* 8.8, 7.7, 4.6, 1.6), 7.13 – 6.99 (2 H, m), 4.60 (2 H, s), 4.25 – 3.82 (1 H, m), 1.63 (1 H, tt, *J* 8.0, 5.3), 1.08 – 0.85 (2 H, m), 0.59 – 0.37 (2 H, m); ^{13}**C NMR** (100 MHz; $CDCl_3$) δ 161.3 (d, *J* 245.4), 157.4, 149.4, 148.3, 139.9, 136.6, 130.3 (d, *J* 4.8), 128.8 (d, *J* 8.1), 127.4 (d, *J* 14.7), 124.2 (d, *J* 3.7), 122.5, 121.5, 115.4 (d, *J* 21.6), 104.6, 42.5 (d, *J* 4.0), 7.3, 3.8; ^{19}**F {^{1}H}** NMR (376 MHz; $CDCl_3$) δ -119.31; **HRMS** *m*/*z* (ESI^{+}) calcd. for $C_{18}H_{18}FN_4$ [M+H]$^{+}$ requires 309.1510, found 309.1518; **LCMS** 2.56 min, 95%, *m/z* 309 [M+H]$^{+}$ (Method A).

4-Cyclopropyl-*N*-(3-fluorobenzyl)-3-(pyridine-2-yl)-1*H*-pyrazol-5-amine, 6h

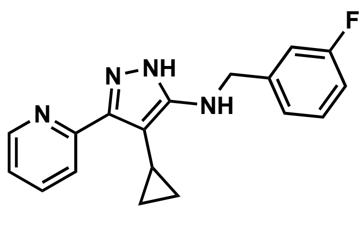

Isolated as a yellow solid (144 mg, 0.47 mmol, 47%); **mp.** 115 – 118 °C; **R$_f$** 0.35 (1:1 EtOAc:*c*Hex); **FT-IR** ν_{max} (ATR)/cm^{-1} 3342, 1587, 1520, 1476, 1445, 1430, 1347, 1312, 1241; **^{1}H NMR** (400 MHz; CDCl$_3$) δ 10.28 (1 H, s), 8.59 (1 H, ddd, *J* 4.9, 1.9, 1.0), 8.00 (1 H, dt, *J* 8.0, 1.1), 7.76 (1 H, td, *J* 7.8, 1.9), 7.30 (1 H, td, *J* 7.8, 5.8), 7.24 – 7.18 (2 H, m), 7.15 (1 H, dt, *J* 9.9, 2.1), 6.99 – 6.90 (1 H, m), 4.55 (2 H, s), 4.04 (1 H, s), 1.65 (1 H, tt, *J* 8.0, 5.3), 1.07 – 0.89 (2 H, m), 0.61 – 0.33 (2 H, m); **^{13}C NMR** (100 MHz; CDCl$_3$) δ 163.2 (d, *J* 245.4), 157.3, 149.5, 148.3, 143.4 (d, *J* 7.0), 140.0, 136.6, 130.1 (d, *J* 8.4), 123.2 (d, *J* 2.9), 122.6, 121.5, 114.6 (d, *J* 21.6), 114.0 (d, *J* 20.9), 104.3, 48.0 (d, *J* 2.2), 7.3, 3.8; **^{19}F {^{1}H}** NMR (376 MHz; CDCl$_3$) δ -113.35; **HRMS** *m*/*z* (ESI$^+$) calcd. for $C_{18}H_{18}FN_4$ [M+H]$^+$ requires 309.1510, found 309.1515; **LCMS** 2.74 min, 98%, *m/z* 309 [M+H]$^+$ (Method A).

4-Cyclopropyl-*N*-(4-fluorobenzyl)-3-(pyridine-2-yl)-1*H*-pyrazol-5-amine, 6i

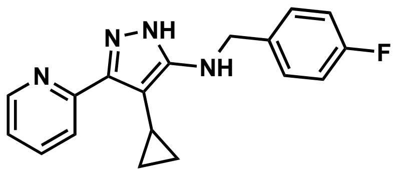

Isolated as a yellow solid (113 mg, 0.37 mmol, 37%); **mp.** 145 – 148 °C; **R$_f$** 0.33 (1:1 EtOAc:*c*Hex); **FT-IR** ν_{max} (ATR)/cm^{-1}; **^{1}H NMR** (400 MHz; CDCl$_3$) δ 10.26 (1 H, s), 8.59 (1 H, ddd, *J* 4.9, 1.9, 1.0), 7.99 (1 H, dt, *J* 8.0, 1.1), 7.75 (1 H, td, *J* 7.8, 1.9), 7.48 – 7.35 (2 H, m), 7.21 (1 H, ddd, *J* 7.7, 4.9, 1.3), 7.11 – 6.91 (2 H, m), 4.51 (2 H, s), 3.97 (1 H, s), 1.63 (1 H, tt, *J* 7.9, 5.2), 1.08 – 0.85 (2 H, m), 0.61 – 0.35 (2 H, m); **^{13}C NMR** (100 MHz; CDCl$_3$) δ 162.1 (d, *J* 244.7), 157.5, 149.5, 148.3, 140.0, 136.6, 136.3 (d, *J* 3.3), 129.4 (d, *J* 8.1), 122.6, 121.5, 115.4 (d, *J* 21.3), 104.3, 47.9, 7.3, 3.8; **^{19}F {^{1}H}** NMR (376 MHz; CDCl$_3$) δ -116.04; **HRMS**

m/z (ESI$^+$) calcd. for $C_{18}H_{17}FN_4$ [M+H]$^+$ requires 309.1510, found 309.1510; **LCMS** 2.72 min, 97%, *m/z* 309 [M+H]$^+$ (Method A).

4-Cyclopropyl-*N*-(2-methoxybenzyl)-3-(pyridine-2-yl)-1*H*-pyrazol-5-amine, 6j

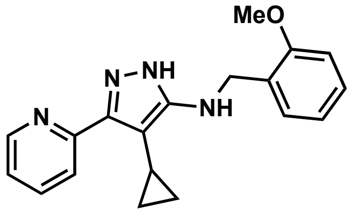

Isolated as a colourless solid (250 mg, 0.78 mmol, 78%); **mp.** 168 – 170 ºC; **R$_f$** 0.39 (1:1 EtOAc:*c*Hex); **FT-IR** ν_{max} (ATR)/cm^{-1} 3414, 3258, 3074, 2999, 2980, 2961, 2915, 2858, 2837, 1598, 1585, 1521; **^{1}H NMR** (400 MHz; CDCl$_3$) δ 9.96 (1 H, s), 8.57 (1 H, d, *J* 5.0), 7.97 (1 H, d, *J* 8.0), 7.74 (1 H, td, *J* 7.8, 1.8), 7.39 (1 H, dd, *J* 7.3, 1.7), 7.28 – 7.14 (2 H, m), 6.97 – 6.82 (2 H, m), 4.52 (2 H, s), 4.38 (1 H, s), 3.89 (3 H, s), 1.70 – 1.55 (1 H, m), 1.05 – 0.86 (2 H, m), 0.48 (2 H, dt, *J* 5.4, 2.9); **^{13}C NMR** (100 MHz; CDCl$_3$) δ 157.9, 157.8, 149.4, 148.4, 139.8, 136.5, 129.9, 128.4, 128.4, 122.4, 121.4, 120.7, 110.3, 104.7, 55.3, 44.7, 7.2, 3.9; **HRMS** *m/z* (ESI$^+$) calcd. for $C_{19}H_{21}N_4O$ [M+H]$^+$ requires 309.1510, found 309.1510; **LCMS** 2.73 min, 98%, *m/z* 321 [M+H]$^+$ (Method A).

4-Cyclopropyl-*N*-(3-methoxybenzyl)-3-(pyridine-2-yl)-1*H*-pyrazol-5-amine, 6k

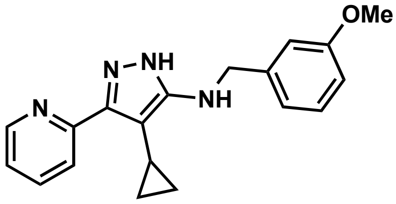

Isolated as a yellow gum (250 mg, 0.78 mmol, 78%); **R**$_f$ 0.39 (1:1 EtOAc:*c*Hex); **FT-IR** ν_{max} (ATR)/cm^{-1} 3228, 1594, 1490, 1463, 1428, 1262, 1145, 1040, 998, 784, 730; ^{1}**H NMR** (400 MHz; CDCl$_3$) δ 9.55 (1 H, s), 8.59 (1 H, d, *J* 5.0), 8.00 (1 H, d, *J* 8.0), 7.75 (1 H, td, *J* 7.8, 1.9), 7.27 (1 H, t, *J* 8.0), 7.21 (1 H, dd, *J* 8.0, 4.3), 7.07 – 6.98 (2 H, m), 6.86 – 6.78 (1 H, m), 4.52 (2 H, s), 4.00 (1 H, s), 3.81 (3 H, s), 1.74 – 1.53 (1 H, m), 1.12 – 0.84 (2 H, m), 0.52 (2 H, dt, *J* 5.4, 2.9); ^{13}**C NMR** (100 MHz; CDCl$_3$) δ 159.9, 157.6, 149.4, 148.4, 142.2, 140.0, 136.6, 129.6, 122.5, 121.5, 120.2, 113.4, 112.7, 104.3, 55.3, 48.6, 7.3, 3.8; **HRMS** *m/z* (ESI$^+$) calcd. for $C_{19}H_{21}N_4O$ [M+H]$^+$ requires 321.1710, found 321.1710; **LCMS** 2.70 min, 95%, *m/z* 321 [M+H]$^+$ (Method A).

4-Cyclopropyl-*N*-(4-methoxybenzyl)-3-(pyridine-2-yl)-1*H*-pyrazol-5-amine, 6l

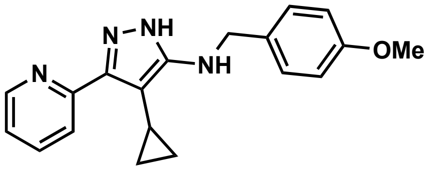

Isolated as a colourless solid (123 mg, 0.38 mmol, 38%); **mp.** 153 – 159 ºC; **R**$_f$ 0.22 (1:1 EtOAc:*c*Hex); **FT-IR** ν_{max} (ATR)/cm^{-1} 3366, 3266, 3089, 3063, 3053, 3015, 2994, 2964, 2833, 1895, 1608, 1587, 1521, 1508; ^{1}**H NMR** (400 MHz; CDCl$_3$) δ 10.25 (1 H, s), 8.59 (1 H, ddd, *J* 4.8, 1.9, 1.0), 7.99 (1 H, dt, *J* 8.0, 1.1), 7.75 (1 H, td, *J* 7.7, 1.8), 7.41 – 7.31 (2 H, m), 7.21 (1 H, ddd, *J* 7.5, 4.8, 1.1), 6.92 – 6.84 (2 H, m), 4.46 (2 H, s), 3.95 (1 H, s), 3.81 (3 H, s), 1.62 (1 H, tt, *J* 8.0, 5.3), 1.05 – 0.85 (2 H, m), 0.59 – 0.37 (2 H, m); ^{13}**C NMR** (100 MHz; CDCl$_3$) δ 158.9, 157.7, 149.4, 148.4, 139.9, 136.6, 132.7, 129.2, 122.5, 121.5, 114.0, 104.3, 55.4, 48.1,

7.3, 3.8; **HRMS** *m/z* (ESI$^+$) calcd. for $C_{19}H_{21}N_4O$ [M+H]$^+$ requires 321.1710, found 321.1712; **LCMS** 2.73 min, 100%, *m/z* 321 [M+H]$^+$ (Method A).

4-Cyclopropyl-*N*-(2-nitrobenzyl)-3-(pyridine-2-yl)-1*H*-pyrazol-5-amine, 6m

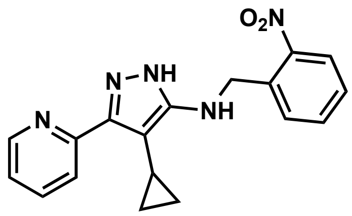

Isolated as a yellow solid (36 mg, 0.11 mmol, 11%); **mp.** 190 – 191 ºC; **R$_f$** 0.32 (1:1 EtOAc:*c*Hex); **FT-IR** ν_{max} (ATR)/cm^{-1} 3142, 3303, 1597, 1510, 1430, 1341, 1135, 1004, 827; **^{1}H NMR** (400 MHz; CDCl$_3$) δ 10.01 (1 H, s), 8.56 (1 H, d, *J* 5.0), 8.03 (1 H, dd, *J* 8.2, 1.4), 7.97 (1 H, d, *J* 8.0), 7.82 – 7.71 (2 H, m), 7.57 (1 H, td, *J* 7.6, 1.4), 7.45 – 7.36 (1 H, m), 7.24 – 7.17 (1 H, m), 4.83 (2 H, d, *J* 6.0), 4.56 (1 H, s), 1.63 (1 H, ddd, *J* 13.2, 8.0, 5.3), 1.06 – 0.93 (2 H, m), 0.48 (2 H, dt, *J* 5.4, 2.9); **^{13}C NMR** (100 MHz; CDCl$_3$) δ 153.1, 149.4, 148.3, 140.0, 138.1, 136.6, 133.6, 131.7, 128.1, 125.1, 122.6, 121.4, 115.4, 104.6, 46.0, 7.3, 3.7; **HRMS** *m/z* (ESI$^+$) calcd. for $C_{18}H_{17}N_5NaO_2$ [M+Na]$^+$ requires 358.1274, found 358.1270; **LCMS** 2.73 min, 95%, *m/z* 336 [M+H]$^+$ (Method A).

4-Cyclopropyl-*N*-(4-nitrobenzyl)-3-(pyridine-2-yl)-1*H*-pyrazol-5-amine, 6n

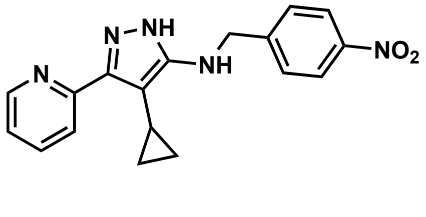

Isolated as a yellow solid (78 mg, 0.23 mmol, 23%); **mp.** 197 – 200 ºC; **R$_f$** 0.26 (1:1 EtOAc:*c*Hex); **FT-IR** ν_{max} (ATR)/cm^{-1}; 3372, 3244, 3083, 3059, 3006, 2983, 2964, 2922, 2905, 2851, 1598, 1591, 1513, 1495; **^{1}H NMR** (400 MHz; $CDCl_3$) δ 10.31 (1 H, s), 8.61 (1 H, ddd, *J* 4.8, 1.8, 0.9), 8.35 – 8.07 (2 H, m), 8.02 (1 H, dt, *J* 8.0, 1.1), 7.79 (1 H, td, *J* 7.7, 1.8), 7.69 – 7.50 (2 H, m), 7.25 (1 H, ddd, *J* 7.5, 4.8, 1.2), 4.68 (2 H, d, *J* 5.5), 4.22 (1 H, s), 1.69 (1 H, tt, *J* 8.0, 5.3), 1.14 – 0.90 (2 H, m), 0.72 – 0.34 (2 H, m); **^{13}C NMR** (100 MHz; $CDCl_3$) δ 156.9, 149.5, 148.7, 148.1, 147.1, 140.1, 136.6, 128.1, 123.9, 122.7, 121.5, 104.2, 47.7, 7.3, 3.8; **HRMS** *m*/*z* (ESI$^+$) calcd. for $C_{18}H_{17}N_5NaO_2$ [M+Na]$^+$ requires 358.1274, found 358.1264; **LCMS** 2.76 min, 100%, *m/z* 336 [M+H]$^+$ (Method A).

4-Cyclopropyl-3-(pyridin-2-yl)-*N*-(3-(trifluoromethyl)benzyl)-1*H*-pyrazol-5-amine, 6o

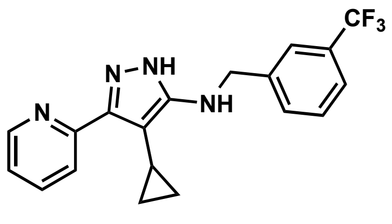

Isolated as a colourless solid (194 mg, 0.54 mmol, 54%); **mp.** 130 – 132 ºC; **R$_f$** 0.40 (1:1 EtOAc:*c*Hex); **FT-IR** ν_{max} (ATR)/cm^{-1} 3339, 3042, 2999, 2976, 2916, 2045, 1975, 1961, 1493, 1427, 1152; **^{1}H NMR** (400 MHz; $CDCl_3$) δ 8.60 (1 H, dd, *J* 5.0, 1.6), 8.02 (1 H, d, *J* 8.0), 7.79 (1 H, td, *J* 7.8, 1.8), 7.72 – 7.60 (2 H, m), 7.58 – 7.40 (2 H, m), 7.31 – 7.21 (1 H, m), 4.63 (2 H, s), 1.79 – 1.55 (1 H, m), 1.05 – 0.94 (2 H, m), 0.62 – 0.46 (2 H, m); **^{13}C NMR** (100 MHz; $CDCl_3$) δ 156.7, 149.1, 147.9, 141.5, 140.1, 137.0, 131.2, 130.9 (d, *J* 32.0), 129.1, 124.5 (q, *J* 3.9), 124.4 (q, *J* 273.1), 124.0 (q, *J* 3.7), 122.8, 121.8, 104.5, 48.1, 7.4, 3.8; **^{19}F{^{1}H} NMR** (376 MHz;

$CDCl_3$) -62.5; **HRMS** *m/z* (ESI$^+$) calcd. for $C_{19}H_{17}F_3N_4$ [M+H]$^+$ requires 359.1478 found 359.1407; 2.93 min, 100%, *m/z* 359 [M+H]$^+$. (Method A)

4-Cyclopropyl-3-(pyridin-2-yl)-*N*-(4-(trifluoromethyl)benzyl)-1*H*-pyrazol-5-amine, 6p

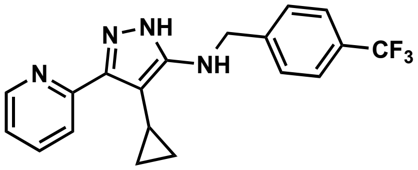

Isolated as a colourless solid (310 mg, 0.81 mmol, 81%); **mp.** 158 – 162 ºC; **R$_f$** 0.46 (1:1 EtOAc:*c*Hex); **FT-IR** ν_{max} (ATR)/cm^{-1} 3214, 3074, 3049, 2998, 2879, 1613, 1586, 1491, 1449, 1425, 1318, 1158, 1142, 1104; **^{1}H NMR** (400 MHz; $CDCl_3$) δ 8.61 (1 H, dd, *J* 5.0, 1.6), 8.05 (1 H, d, *J* 8.0), 7.83 (1 H, td, *J* 7.8, 1.8), 7.59 (2 H, d, *J* 8.2), 7.54 (2 H, d, *J* 8.3), 7.31 – 7.26 (1 H, m), 4.64 (2 H, s), 1.66 (1 H, tt, *J* 8.0, 5.3), 1.05 – 0.94 (2 H, m), 0.56 – 0.45 (2 H, m); **^{13}C NMR** (100 MHz; $CDCl_3$) δ 156.4, 148.7, 147.5, 144.4, 140.0, 137.5, 129.5 (q, *J* 32.2), 128.0, 125.6 (q, *J* 3.8), 124.4 (q, *J* 271.8). 122.9, 122.1, 104.5, 48.1, 7.4, 3.8; **^{19}F{^{1}H} NMR** (376 MHz; $CDCl_3$) δ -62.4; **HRMS** *m/z* (ESI$^+$) calcd. for $C_{19}H_{17}F_3N_4$ [M+H]$^+$ requires 359.1478 found 309.1410; **LCMS** 2.95 min, 98%, *m/z* 359 [M+H]$^+$ (Method A).

4-Cyclopropyl-3-(pyridin-2-yl)-*N*-(2-(trifluoromethoxy)benzyl)-1*H*-pyrazol-5-amine, 6q

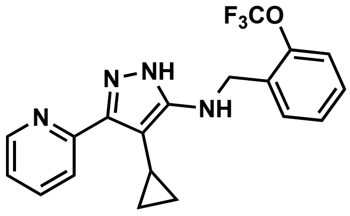

Isolated as a colourless solid (36 mg, 0.09 mmol, 9%); **mp.** 103 – 105 ºC; **R_f** 0.61 (1:1 EtOAc:*c*Hex); **FT-IR** ν_{max} (ATR)/cm^{-1} 3295, 1526, 1481, 1361, 1260, 1204, 1151, 1009, 921, 786, 705, 631; **^{1}H NMR** (400 MHz; CDCl$_3$) δ δ_H (400 MHz, CDCl$_3$) 8.59 (1 H, d, *J* 4.8), 7.99 (1 H, d, *J* 8.0), 7.76 (1 H, td, *J* 7.8, 1.8), 7.60 (1 H, d, *J* 7.3), 7.35 – 7.14 (5 H, m), 4.64 (2 H, s), 1.63 (1 H, ddd, *J* 10.6, 8.2, 5.3), 1.04 – 0.91 (2 H, m), 0.49 (2 H, dt, *J* 5.4, 2.9); **^{13}C NMR** (100 MHz; CDCl$_3$) δ 157.0, 149.3, 148.2, 147.6, 140.0, 136.7, 132.9, 130.5, 128.5, 127.0, 122.6, 121.6, 120.8 (q, *J* 257.3), 120.5, 104.5, 43.2, 7.2, 3.7; **^{19}F{^{1}H} NMR** (376 MHz; CDCl$_3$) δ -57.0; **HRMS** *m*/*z* (ESI$^+$) calcd. for $C_{19}H_{18}F_3N_4O$ [M+H]$^+$ requires 375.1427 found 375.1424; **LCMS** 2.95 min, 100%, *m/z* 375 [M+H]$^+$ (Method A).

4-Cyclopropyl-3-(pyridin-2-yl)-*N*-(3-(trifluoromethoxy)benzyl)-1*H*-pyrazol-5-amine, 6r

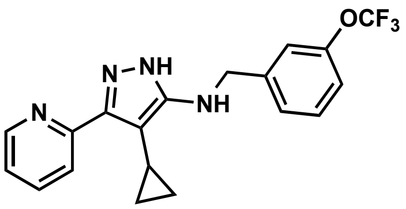

Isolated as a colourless solid (153 mg, 0.41 mmol, 41%); **mp.** 107 – 109 ºC; **R_f** 0.65 (1:1 EtOAc:*c*Hex); **FT-IR** ν_{max} (ATR)/cm^{-1} 3406, 3342, 3051, 2991, 2908, 1609, 1582, 1560, 1515; **^{1}H NMR** (400 MHz; CDCl$_3$) δ 8.60 (1 H, dd, *J* 5.0, 1.6), 8.02 (1 H, d, *J* 8.1), 7.79 (1 H, td, *J* 7.8, 1.8), 7.42 – 7.20 (4 H, m), 7.11 (1 H, dd, *J* 6.3, 3.5), 4.59 (2 H, s), 1.65 (1 H, tt, *J* 7.9, 5.2), 1.05 – 0.92 (2 H, m), 0.55 – 0.46 (2 H, m); **^{13}C NMR** (100 MHz; CDCl$_3$) δ 156.7, 149.6, 149.6, 149.1, 147.9, 142.9, 140.1, 137.0, 129.9, 126.1, 122.8, 121.9, 120.6 (q, *J* 257.0), 120.2, 119.5, 104.4, 48.0, 7.4, 3.8; **^{19}F{^{1}H} NMR** (376 MHz; CDCl$_3$) δ -57.7; **HRMS** *m*/*z* (ESI$^+$) calcd. for

$C_{19}H_{17}F_3N_4O$ $[M+H]^+$ requires 375.1427 found 375.1431; **LCMS** 3.00 min, 100%, *m/z* 375 $[M+H]^+$ (Method A).

4-Cyclopropyl-3-(pyridin-2-yl)-*N*-(4-(trifluoromethoxy)benzyl)-1*H*-pyrazol-5-amine, 6s

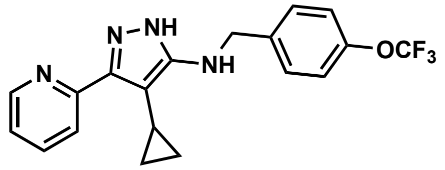

Isolated as a colourless solid (155 mg, 0.41 mmol, 41%); **mp.** 118 – 122 ºC; **R$_f$** 0.74 (1:1 EtOAc:*c*Hex); **FT-IR** ν_{max} (ATR)/cm^{-1} 3225, 1784, 1513, 1491, 1101, 1076, 1024, 996, 896; **^{1}H NMR** (400 MHz; CDCl$_3$) δ 8.60 (1 H, dd, *J* 4.9, 1.5), 8.02 (1 H, d, *J* 8.0), 7.79 (1 H, td, *J* 7.8, 1.8), 7.46 (2 H, d, *J* 8.4), 7.28 – 7.22 (1 H, m), 7.18 (2 H, d, *J* 8.2), 4.57 (2 H, s), 1.65 (1 H, tt, *J* 7.9, 5.2), 1.16 – 0.85 (2 H, m), 0.64 – 0.42 (2 H, m); **^{13}C NMR** (100 MHz; CDCl$_3$) δ 156.7, 149.1, 148.4, 147.9, 140.2, 139.2, 137.0, 129.1, 122.8, 121.8, 121.2, 120.3 (q, *J* 255.9), 104.4, 47.8, 7.4, 3.8; **^{19}F{^{1}H} NMR** (376 MHz; CDCl$_3$) δ -57.9; **HRMS** *m*/*z* (ESI$^+$) calcd. for $C_{19}H_{17}F_3N_4O$ $[M+H]^+$ requires 375.1427 found 375.1428; **LCMS** 3.01 min, 98%, *m/z* 375 $[M+H]^+$ (Method A).

4-Cyclopropyl-*N*-(3-(pentafluoro-λ^6-sulfaneyl)benzyl)-3-(pyridin-2-yl)-1*H*-pyrazol-5-amine, 6t

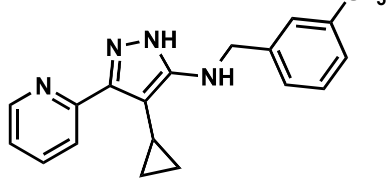

Isolated as a colourless solid (114 mg, 0.27 mmol, 73%); **FT-IR** ν_{max} (ATR)/cm^{-1} 3341, 3155, 3062, 3005, 2978, 2925, 1592, 1571, 1525, 1497, 1483, 1460, 1446, 1430, 1392, 1341, 1312, 1299, 1272, 1241, 1169, 1152, 1109, 1097, 1055, 1024, 1005, 910, 891, 878, 851, 821, 788, 776, 740, 734, 721, 687, 666; ^{1}**H NMR** (300 MHz; $CDCl_3$) δ 8.63 (1 H, ddd, *J* 4.9, 1.8, 1.0), 8.00 (1 H, dt, *J* 8.0, 1.1), 7.84 (1 H, t, *J* 1.9), 7.76 (1 H, ddd, *J* 8.1, 7.6, 1.8), 7.66 – 7.53 (2 H, m), 7.44 – 7.33 (1 H, m), 7.21 (1 H, ddd, *J* 7.6, 4.9, 1.2), 4.63 (2 H, s), 4.12 (0 H, d, *J* 7.1), 1.72 – 1.58 (1 H, m), 1.07 – 0.93 (2 H, m), 0.59 – 0.44 (2 H, m); ^{13}**C NMR** (75 MHz; $CDCl_3$) δ 156.9, 154.2 (pent, *J* 16.8), 149.4, 148.3, 142.2, 140.0, 136.6, 130.9, 128.8, 125.2 (pent, *J* 4.7), 124.5 (pent, *J* 4.7), 122.5, 121.6, 104.4, 48.0, 7.3, 3.8; **HRMS** *m/z* (ESI$^+$) calcd. for $C_{18}H_{18}F_5N_4S$ [M+H]$^+$ requires 417.1167 found 417.1160 (Method B)

4-Cyclopropyl-*N*-(4-(pentafluoro-λ^6-sulfaneyl)benzyl)-3-(pyridin-2-yl)-1*H*-pyrazol-5-amine, 6u

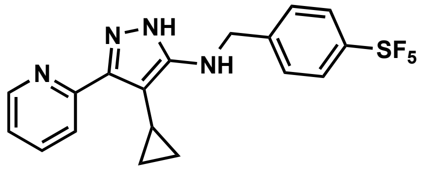

Isolated as a colourless solid (80 mg, 0.19 mmol, 63%); **FT-IR** ν_{max} (ATR)/cm^{-1} 3355, 3087, 3010, 2918, 1593, 1565, 1525, 1498, 1476, 1431, 1417, 1385, 1364, 1312, 1237, 1208, 1185, 1146, 1092, 1055, 1022, 1006, 969, 885, 843, 828, 815, 792, 782, 741, 729, 662; ^{1}**H NMR** (300 MHz; $CDCl_3$) δ 8.60 (1 H, ddd, *J* 4.8, 1.8, 0.9), 8.00 (1 H, dt, *J* 8.0, 1.1), 7.84 – 7.63 (3 H, m), 7.51 (2 H, dq, *J* 7.8, 1.0), 7.22 (1 H, ddd, *J* 7.6, 4.9, 1.2), 4.61 (2 H, s), 4.12 (0 H, d, *J* 7.2), 1.66 (1 H, tt, *J* 8.0, 5.2), 1.07 – 0.93 (2 H, m), 0.63 – 0.43 (2 H, m); ^{13}**C NMR** (75 MHz; $CDCl_3$) δ 157.0, 154.1 (pent, *J* 16.8), 149.5, 148.3, 144.9, 140.1, 136.6, 127.7, 126.2 (pent, *J* 4.8),

122.6, 121.5, 104.3, 47.6, 7.3, 3.8; **HRMS** *m/z* (ESI$^+$) calcd. for $C_{18}H_{18}F_5N_4S$ $[M+H]^+$ requires 417.1167 found 417.1156 (Method B).

4-Cyclopropyl-3-(pyridin-2-yl)-*N*-(3-((trifluoromethyl)thio)benzyl)-1*H*-pyrazol-5-amine, 6v

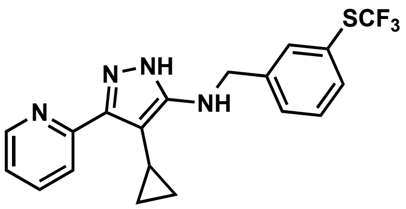

Isolated as a colourless solid (148 mg, 0.38 mmol, 53%); **mp.** 114 – 115 ºC; **FT-IR** ν_{max} (ATR)/cm^{-1} 3356, 3071, 3001, 2980, 2926, 1593, 1564, 1521, 1497, 1475, 1429, 1417, 1346, 1310, 1234, 1107, 1072, 1052, 1023, 1006, 969, 901, 886, 824, 786, 778, 755, 741, 729, 688; **^{1}H NMR** (300 MHz; $CDCl_3$) δ 8.58 (1 H, ddd, *J* 4.8, 1.8, 1.0), 8.00 (1 H, dt, *J* 8.0, 1.1), 7.76 (1 H, ddd, *J* 8.0, 7.5, 1.8), 7.72 (1 H, dd, *J* 1.2, 0.6), 7.61 – 7.50 (2 H, m), 7.45 – 7.34 (1 H, m), 7.22 (1 H, ddd, *J* 7.5, 4.8, 1.1), 4.58 (2 H, d, *J* 5.6), 4.07 (1 H, s), 1.65 (1 H, tt, *J* 8.0, 5.2), 1.07 – 0.90 (2 H, m), 0.61 – 0.42 (2 H, m); **^{13}C NMR** (75 MHz; $CDCl_3$) δ 157.3, 149.5, 148.3, 142.5, 140.1, 136.6, 135.4, 134.9, 130.3, 129.8 (q, *J* 307.2) 129.7, 122.6, 124.6 (q, *J* 2.2), 121.5, 104.3, 47.9, 7.3, 3.8; **HRMS** *m/z* (ESI$^+$) calcd. for $C_{19}H_{18}F_3N_4S$ $[M+H]^+$ requires 391.1199 found 391.1191 (Method B)

4-Cyclopropyl-3-(pyridin-2-yl)-*N*-(4-((trifluoromethyl)thio)benzyl)-1*H*-pyrazol-5-amine, 6w

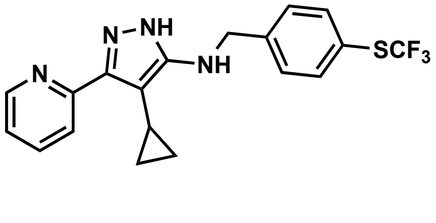

Isolated as a colourless solid (172 mg, 0.44 mmol, 62%); **mp.** 143 – 145 ºC; **FT-IR** ν_{max} (ATR)/cm^{-1} 3280, 3087, 3011, 2985, 1589, 1566, 1519, 1492, 1451, 1426, 1407, 1334, 1291, 1264, 1242, 1183, 1139, 1116, 1090, 1051, 1017, 998, 972, 899, 820, 787, 741, 733, 724, 669; **^{1}H NMR** (300 MHz; $CDCl_3$) δ 10.53 (1 H, s), 8.60 (1 H, ddd, *J* 4.9, 1.8, 1.0), 8.00 (1 H, dt, *J* 8.0, 1.1), 7.76 (1 H, ddd, *J* 8.0, 7.5, 1.8), 7.66 – 7.55 (2 H, m), 7.52 – 7.42 (2 H, m), 7.21 (1 H, ddd, *J* 7.6, 4.9, 1.2), 4.72 – 4.46 (2 H, m), 4.10 (1 H, d, *J* 7.3), 1.65 (1 H, tt, *J* 8.0, 5.2), 1.09 – 0.91 (2 H, m), 0.63 – 0.41 (2 H, m); **^{13}C NMR** (75 MHz; $CDCl_3$) δ 157.2, 149.5, 148.3, 144.2, 140.1, 136.6, 136.6, 129.8 (q, *J* 307.9), 128.7, 123.7 (q, *J* 2.0), 122.6, 121.5, 104.3, 47.9, 7.3, 3.8; **HRMS** *m*/*z* (ESI$^+$) calcd. for $C_{19}H_{18}F_3N_4S$ $[M+H]^+$ requires 391.1199 found 391.1187 (Method B)

***N*-([1,1'-Biphenyl]-3-ylmethyl)-4-cyclopropyl-3-(pyridin-2-yl)-1*H*-pyrazol-5-amine, x**

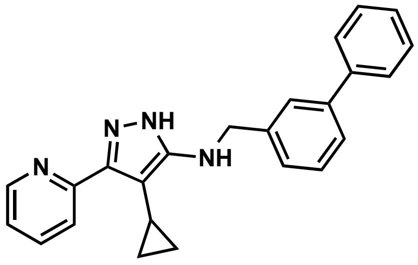

Isolated as a colourless solid (210 mg, 0.57 mmol, 57%); **mp.** 117 – 118 ºC; **R$_f$** 0.50 (1:1 EtOAc:*c*Hex); **FT-IR** ν_{max} (ATR)/cm^{-1} 3106, 2985, 2959, 2883, 2843, 1591, 1501, 1427, 1357; **^{1}H NMR** (400 MHz; $CDCl_3$) δ 8.62 (1 H, dd, *J* 4.9, 1.6), 8.05 (1 H, d, *J* 8.0), 7.82 (1 H, td, *J* 7.8, 1.8), 7.67– 7.65 (1 H, m), 7.63 – 7.57 (2 H, m), 7.51 (1 H, dd, *J* 6.8, 3.1), 7.47 – 7.38 (4 H, m), 7.38 – 7.26 (2 H, m), 4.65 (2 H, s), 1.65 (1 H, tt, *J* 7.9, 5.2), 1.08 – 0.88 (2 H, m), 0.68 – 0.45 (2 H, m); **^{13}C NMR** (100 MHz; $CDCl_3$) δ 156.4, 148.7, 147.5, 141.7, 141.2, 140.4, 140.2, 137.4, 129.2, 128.9, 127.4, 127.3, 126.9, 126.9, 126.3, 123.0, 122.2, 104.5, 48.9, 7.4, 3.8;

HRMS *m/z* (ESI+) calcd. for $C_{24}H_{25}N_4$ $[M+H]^+$ requires 367.1917 found 367.1919; **LCMS** 3.06 min, 100%, *m/z* 367 $[M+H]^+$ (Method A).

N-([1,1'-Biphenyl]-4-ylmethyl)-4-cyclopropyl-3-(pyridin-2-yl)-1H-pyrazol-5-amine, 6y

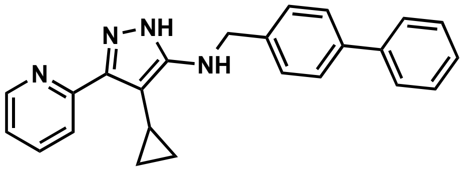

Isolated as a pale yellow solid (204 mg, 0.56 mmol, 56%); **mp.** 177 – 178 ºC; R_f 0.25 (1:1 EtOAc:*c*Hex); **FT-IR** ν_{max} (ATR)/cm^{-1} 3221, 1592, 1516, 1496, 1465, 1429; ^{1}**H NMR** (400 MHz; $CDCl_3$) δ 10.15 (1 H, s), 8.59 (1 H, ddd, *J* 4.9, 1.9, 1.0), 8.01 (1 H, dt, *J* 7.9, 1.1), 7.76 (1 H, td, *J* 7.8, 1.9), 7.64 – 7.55 (4 H, m), 7.55 – 7.47 (2 H, m), 7.47 – 7.39 (2 H, m), 7.39 – 7.29 (1 H, m), 7.22 (1 H, ddd, *J* 7.5, 4.8, 1.1), 4.59 (2 H, s), 4.05 (1 H, s), 1.66 (1 H, tt, *J* 8.0, 5.3), 1.08 – 0.92 (2 H, m), 0.61 – 0.44 (2 H, m); ^{13}**C NMR** (100 MHz; $CDCl_3$) δ 157.6, 149.3, 148.2, 141.0, 140.0, 139.9, 139.5, 136.4, 128.8, 128.2, 127.3, 127.2, 127.1, 122.4, 121.3, 104.2, 48.2, 7.2, 3.7; **HRMS** *m/z* (ESI+) calcd. for $C_{24}H_{23}N_4$ $[M+H]^+$ requires 367.1917, found 367.1919; **LCMS** 3.07 min, 95%, *m/z* 367 $[M+H]^+$ (Method A).

3-(((4-Cyclopropyl-3-(pyridin-2-yl)-1H-pyrazol-5-yl)amino)methyl)benzonitrile, 6z

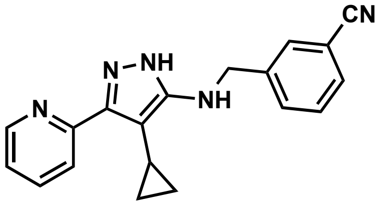

Isolated as a colourless solid (78 mg, 0.25 mmol, 25%); **mp.** 138 – 140 ºC; **R**$_f$ 0.24 (1:1 EtOAc:*c*Hex); **FT-IR** ν_{max} (ATR)/cm^{-1} 3410, 3133, 3052, 2987, 2907, 2218, 1589, 1563, 1519, 1495, 1477; ^{1}**H NMR** (500 MHz; CDCl$_3$) δ 8.61 (1 H, ddd, *J* 5.0, 1.8, 0.9), 8.05 (1 H, dt, *J* 8.0, 1.1), 7.84 (1 H, td, *J* 7.8, 1.8), 7.72 (1 H, d, *J* 1.8), 7.67 (1 H, dt, *J* 7.7, 1.5), 7.54 (1 H, dt, *J* 7.7, 1.5), 7.43 (1 H, t, *J* 7.7), 7.29 (1 H, ddd, *J* 7.6, 5.0, 1.1), 4.61 (2 H, s), 1.66 (1 H, tt, *J* 8.0, 5.2), 1.10 – 0.95 (2 H, m), 0.55 – 0.50 (2 H, m); ^{13}**C NMR** (125 MHz; CDCl$_3$) 156.2, 148.6, 147.5, 142.1, 139.9, 137.5, 132.2, 131.1, 130.9, 129.4, 122.9, 122.1, 119.2, 112.6, 104.6, 47.7, 7.4, 3.8; **HRMS** *m*/*z* (ESI$^+$) calcd. for $C_{19}H_{17}F_3N_4O$ [M+H]$^+$ requires 316.1557 found 316.1556; **LCMS** 2.63 min, 100%, *m/z* 316 [M+H]$^+$ (Method A).

4-Cyclopropyl-*N*-(4-(methylsulfonyl)benzyl)-3-(pyridin-2-yl)-1*H*-pyrazol-5-amine, 6aa

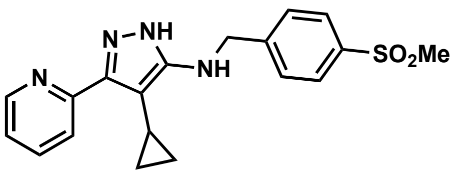

Isolated as a colourless solid (101 mg, 0.27 mmol, 27%); **mp.** 166 – 169 ºC; **R**$_f$ 0.14 (1:1 EtOAc:*c*Hex); **FT-IR** ν_{max} (ATR)/cm^{-1} 3158, 3046, 2992, 2914, 1562, 1426, 1403, 1013, 1293, 1139; ^{1}**H NMR** (400 MHz; CDCl$_3$) δ 8.61 (1 H, d, *J* 4.9), 8.20 – 8.00 (1 H, m), 7.95 – 7.77 (3 H, m), 7.67 – 7.59 (2 H, m), 7.33 – 7.25 (1 H, m), 4.68 (2 H, s), 3.03 (3 H, s), 1.67 (1 H, tt, *J* 8.0, 5.2), 1.06 – 0.96 (2 H, m), 0.53 (2 H, dd, *J* 5.4, 1.9); ^{13}**C NMR** (100 MHz; CDCl$_3$) δ 156.2, 148.8, 147.6, 147.1, 140.1, 139.2, 137.4, 128.4, 127.8, 123.0, 122.0, 104.4, 47.9, 44.7, 7.4, 3.8; **HRMS** *m*/*z* (ESI$^+$) calcd. for $C_{19}H_{21}N_4O_2S$ [M+H]$^+$ requires 369.1380 found 369.1377; **LCMS** 2.68 min, 95%, *m/z* 369 [M+H]$^+$ (Method A).

***N*-(3-(Benzyloxy)benzyl)-4-cyclopropyl-3-(pyridin-2-yl)-1*H*-pyrazol-5-amine, 6ab**

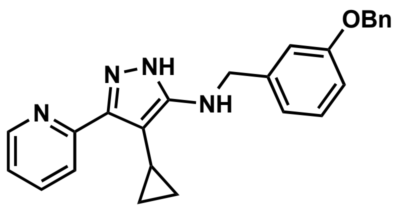

Isolated as a colourless solid (206 mg, 0.52 mmol, 52%); **mp.** 142 – 144 °C; $\mathbf{R_f}$ 0.32 (1:1 EtOAc:*c*Hex); **FT-IR** ν_{max} (ATR)/cm^{-1}; 3300, 1494, 1264, 1157, 1033, 739, 696; **^{1}H NMR** (400 MHz; $CDCl_3$) δ 10.14 (1 H, s), 8.59 (1 H, ddd, *J* 4.9, 1.9, 1.0), 8.00 (1 H, dt, *J* 8.0, 1.1), 7.76 (1 H, td, *J* 7.7, 1.8), 7.46 – 7.40 (2 H, m), 7.40 – 7.34 (2 H, m), 7.34 – 7.28 (1 H, m), 7.26 (1 H, d, *J* 8.0), 7.21 (1 H, ddd, *J* 7.5, 4.8, 1.1), 7.09 (1 H, dd, *J* 2.6, 1.7), 7.03 (1 H, ddd, *J* 7.5, 1.6, 0.9), 6.89 (1 H, ddd, *J* 8.3, 2.6, 1.1), 5.07 (2 H, s), 4.52 (2 H, s), 3.99 (1 H, s), 1.63 (1 H, tt, *J* 8.0, 5.3), 1.02 – 0.93 (2 H, m), 0.54 – 0.47 (2 H, m); **^{13}C NMR** (100 MHz; $CDCl_3$) δ 159.2, 157.6, 149.5, 148.4, 142.3, 140.0, 137.2, 136.6, 129.7, 128.7, 128.0, 127.6, 122.5, 121.5, 120.4, 114.3, 113.7, 104.3, 70.1, 48.6, 7.3, 3.8; **HRMS** *m*/*z* (ESI$^+$) calcd. for $C_{25}H_{25}N_4O$ $[M+H]^+$ requires 397.2023, found 397.2025; **LCMS** 3.05 min, 100%, *m/z* 397 $[M+H]^+$ (Method A).

4-Cyclopropyl-*N*-(3-bromobenzyl)-3-(pyridine-2-yl)-1*H*-pyrazol-5-amine, 6ac

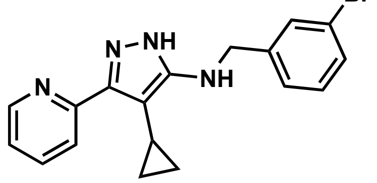

Isolated as a colourless solid (166 mg, 0.45 mmol, 45%); **mp.** 147 – 150 ºC; **R$_f$** 0.21 (1:1 EtOAc:*c*Hex); **FT-IR** ν_{max} (ATR)/cm^{-1} 3362, 1588, 1519, 1472, 1339, 1307, 1142, 1006; **^{1}H NMR** (400 MHz; CDCl$_3$) δ 10.15 (1 H, s), 8.58 (1 H, ddd, *J* 4.8, 1.8, 1.0), 8.00 (1 H, dt, *J* 7.9, 1.1), 7.76 (1 H, td, *J* 7.7, 1.8), 7.59 (1 H, t, *J* 1.9), 7.41 – 7.32 (2 H, m), 7.25 – 7.16 (2 H, m), 4.52 (2 H, d, *J* 5.1), 4.03 (1 H, s), 1.65 (1 H, tt, *J* 7.9, 5.3), 1.12 – 0.87 (2 H, m), 0.64 – 0.41 (2 H, m); **^{13}C NMR** (100 MHz; CDCl$_3$) δ 157.3, 149.5, 148.3, 143.1, 140.0, 139.3, 136.6, 130.8, 130.2, 130.2, 126.4, 122.6, 121.5, 104.3, 47.9, 7.3, 3.8; **HRMS** *m*/*z* (ESI$^+$) calcd. for $C_{18}H_{18}BrN_4$ [M+H]$^+$ requires 369.0709, found 369.0702; **LCMS** 2.89 min, 95%, *m/z* 369 [M+H]$^+$ (Method A).

4-Cyclopropyl-*N*-(4-propoxybenzyl)-3-(pyridin-2-yl)-1*H*-pyrazol-5-amine, 6ad

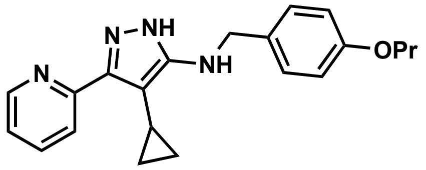

Isolated as a beige solid (24 mg, 0.07 mmol, 7%); **mp.** 115 – 117 ºC; **R$_f$** 0.47 (1:1 EtOAc:*c*Hex); **FT-IR** ν_{max} (ATR)/cm^{-1} 2964, 1591, 1510, 1465, 1241, 1174, 998, 975, 787, 731, 624; **^{1}H NMR** (400 MHz; CDCl$_3$) δ 8.75 – 8.46 (1 H, m), 8.01 (1 H, d, *J* 8.0), 7.89 – 7.63 (1 H, m), 7.34 (2 H, d, *J* 8.6), 7.22 (1 H, dd, *J* 7.6, 5.0), 6.90 – 6.86 (2 H, m), 4.47 (2 H, s), 3.91 (2 H, t, *J* 6.6), 1.80 (2 H, tq, *J* 7.1, 6.6), 1.62 (1 H, td, *J* 8.1, 4.1), 1.03 (3 H, t, *J* 7.5), 0.95 (2 H, dd, *J* 8.0, 2.0), 0.53 – 0.44 (2 H, m); **^{13}C NMR** (100 MHz; CDCl$_3$) δ 158.5, 157.2, 149.3, 148.3, 140.2, 136.7, 132.3, 129.2, 122.6, 121.7, 114.7, 104.3, 69.7, 48.2, 22.7, 10.7, 7.3, 3.8.; **HRMS** *m*/*z* (ESI$^+$) calcd. for $C_{21}H_{15}N_4O$ [M+H]$^+$ requires 349.2023 found 349.2024; **LCMS** 3.00 min, 98%, *m/z* 349 [M+H]$^+$.

4-Cyclopropyl-*N*-(4-isopropoxybenzyl)-3-(pyridin-2-yl)-1*H*-pyrazol-5-amine, 6ae

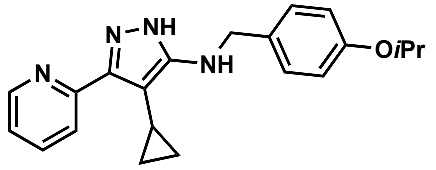

Isolated as a colourless solid (244 mg, 0.70 mmol, 70%); **mp.** 106 – 108 ºC; **R_f** 0.14 (1:1 EtOAc:*c*Hex); **FT-IR** ν_{max} (ATR)/cm^{-1} 3369, 3233, 3044, 2967, 2921, 2864, 1606, 1584, 1562, 1503, 1490; **^{1}H NMR** (400 MHz; $CDCl_3$) δ 8.68 – 8.52 (1 H, m), 7.99 (1 H, d, *J* 8.0), 7.75 (1 H, td, *J* 7.8, 1.8), 7.34 (2 H, d, *J* 8.4), 7.24 – 7.18 (1 H, m), 6.87 (2 H, d, *J* 8.7), 4.52 (1 H, dq, *J* 13.2, 6.6), 4.47 (2 H, s), 1.62 (1 H, tt, *J* 8.0, 5.3), 1.33 (6 H, d, *J* 6.0), 1.05 – 0.88 (2 H, m), 0.49 (2 H, dd, *J* 5.3, 1.9); **^{13}C NMR** (100 MHz; $CDCl_3$) δ 157.4, 157.2, 149.4, 148.3, 140.1, 136.6, 132.3, 129.2, 122.6, 121.6, 116.0, 104.3, 70.0, 48.2, 22.2, 7.3, 3.8; **HRMS** *m*/*z* (ESI$^+$) calcd. for $C_{21}H_{15}N_4O$ $[M+H]^+$ requires 349.2023 found 349.2016; **LCMS** 2.98 min, 100%, *m/z* 349 $[M+H]^+$ (Method A).

4-Cyclopropyl-*N*-(3-(difluoromethoxy)benzyl)-3-(pyridin-2-yl)-1*H*-pyrazol-5-amine, 6af

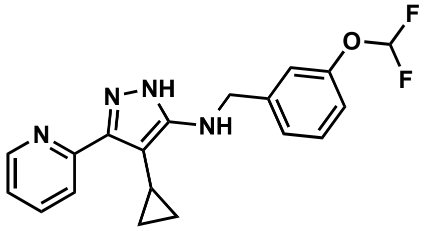

Isolated as a colourless solid (205 mg, 0.57 mmol, 57%); **mp.** 81 – 82 ºC; $\mathbf{R_f}$ 0.39 (1:1 EtOAc:*c*Hex); **FT-IR** ν_{max} (ATR)/cm^{-1} 3426, 3351, 3134, 3048, 2986, 2926, 1588, 1524, 1499, 1478, 1146; ^{1}**H NMR** (400 MHz; $CDCl_3$) δ 8.60 (1 H, dd, *J* 5.0, 1.5), 8.04 (1 H, d, *J* 8.0), 7.81 (1 H, td, *J* 7.8, 1.7), 7.48 – 7.16 (4 H, m), 7.04 – 6.95 (1 H, m), 6.52 (1 H, t, *J* 74.1), 4.58 (2 H, s), 1.65 (1 H, tt, *J* 7.9, 5.2), 1.13 – 0.89 (2 H, m), 0.59 – 0.47 (2 H, m); ^{13}**C NMR** (100 MHz; $CDCl_3$) δ 156.5, 151.7 (t, *J* 2.9), 148.9, 147.7, 142.7, 140.1, 137.3, 130.0, 124.7, 122.9, 122.0, 118.7, 118.1, 116.2 (t, *J* 259.5), 104.5, 48.1, 7.4, 3.8. ^{19}**F{^{1}H} NMR** (376 MHz; $CDCl_3$) δ -80.6; **HRMS** *m/z* (ESI$^+$) calcd. for $C_{19}H_{17}F_3N_4$ [M+H]$^+$ requires 357.1521 found 357.1522; **LCMS** 2.87 min, 100%, *m/z* 357 [M+H]$^+$ (Method A).

4-Cyclopropyl-*N*-(3-(3,5-dimethyl-1*H*-pyrazol-1-yl)benzyl)-3-(pyridin-2-yl)-1*H*-pyrazol-5-amine, 6ag

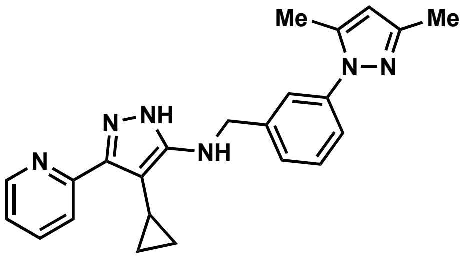

Isolated as a colourless solid (310 mg, 0.81 mmol, 81%); **mp.** 149 – 152 ºC; $\mathbf{R_f}$ 0.14 (1:1 EtOAc:*c*Hex); **FT-IR** ν_{max} (ATR)/cm^{-1} 3192, 3065, 2967, 2921, 2858, 1591, 1493, 1380, 1149, 1024, 1001, 787, 743, 624; ^{1}**H NMR** (400 MHz; $CDCl_3$) δ 10.31 (1 H, s), 8.59 (1 H, ddd, *J* 4.8, 1.9, 0.9), 8.03 – 7.95 (1 H, m), 7.75 (1 H, tt, *J* 7.8, 2.0), 7.51 (1 H, dq, *J* 1.5, 0.9), 7.44 – 7.38 (2 H, m), 7.32 (1 H, qq, *J* 4.5, 2.4), 7.21 (1 H, ddd, *J* 7.5, 4.8, 1.1), 5.97 (1 H, s), 4.59 (2 H, s), 4.05 (1 H, s), 2.29 (3 H, s), 2.27 (3 H, s), 1.64 (1 H, qt, *J* 8.0, 4.8), 0.98 (2 H, dddd, *J* 11.8, 8.0,

6.0, 3.9), 0.60 – 0.42 (2 H, m); **^{13}C NMR** (100 MHz; $CDCl_3$) δ 157.4, 149.4, 149.0, 148.3, 141.9, 140.2, 140.0, 139.6, 136.6, 129.2, 126.7, 124.1, 123.4, 122.5, 121.5, 107.0, 104.3, 48.2, 13.6, 12.5, 7.4, 3.8; **HRMS** *m/z* (ESI$^+$) calcd. for $C_{23}H_{24}N_6$ $[M+H]^+$ requires 385.2135, found 385.2139; **LCMS** 2.92 min, 99%, *m/z* 385 $[M+H]^+$ (Method A).

4-Cyclopropyl-*N*-(3,4-dichlorobenzyl)-3-(pyridin-2-yl)-1*H*-pyrazol-5-amine, 6ah

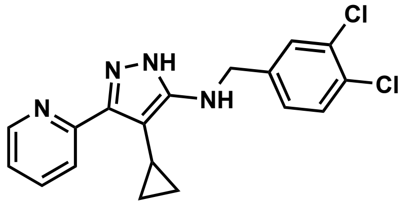

Isolated as a yellow solid (210 mg, 0.58 mmol, 58%); **mp.** 161 – 164 °C; **R$_f$** 0.24 (1:1 EtOAc:*c*Hex); **FT-IR** ν_{max} (ATR)/cm^{-1} 1 3340, 1590, 1518, 1468, 1332, 1053; **^{1}H NMR** (400 MHz; $CDCl_3$) δ 10.26 (1 H, s), 8.59 (1 H, ddd, *J* 4.9, 1.9, 1.0), 7.99 (1 H, dt, *J* 8.0, 1.1), 7.76 (1 H, td, *J* 7.7, 1.8), 7.52 (1 H, d, *J* 2.1), 7.39 (1 H, d, *J* 8.3), 7.28 – 7.24 (2 H, m), 7.22 (1 H, ddd, *J* 7.5, 4.8, 1.1), 4.50 (2 H, d, *J* 6.0), 4.26 – 3.93 (1 H, m), 1.65 (1 H, tt, *J* 7.9, 5.3), 1.12 – 0.89 (2 H, m), 0.64 – 0.41 (2 H, m); **^{13}C NMR** (100 MHz; $CDCl_3$) δ 156.9, 149.4, 148.1, 141.0, 139.9, 136.5, 132.4, 130.8, 130.4, 129.5, 126.9, 122.5, 121.3, 104.1, 47.2, 7.2, 3.7; **HRMS** *m/z* (ESI$^+$) calcd. for $C_{18}H_{17}{}^{35}Cl_2N_4$ $[M+H]^+$ requires 359.0525, found 359.0524; **LCMS** 3.00 min, 95%, *m/z* 359 $[M+H]^+$ (Method A).

***N*-(4-Bromo-3-chlorobenzyl)-4-cyclopropyl-3-(pyridin-2-yl)-1*H*-pyrazol-5-amine, 6ai**

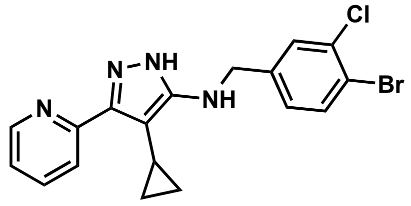

Isolated as a colourless solid (157 mg, 0.39 mmol, 39%); **mp.** 150 – 155 ºC; $\mathbf{R_f}$ 0.35 (1:1 EtOAc:*c*Hex); **FT-IR** ν_{max} (ATR)/cm^{-1}; 3340, 1590, 1516, 1498, 1477, 1464, 1424, 1329, 1054, 1015, 741, 624, 571; ^{1}**H NMR** (400 MHz; CDCl$_3$) δ 8.59 (1 H, ddd, *J* 4.9, 1.9, 1.0), 7.99 (1 H, dt, *J* 8.0, 1.1), 7.76 (1 H, td, *J* 7.7, 1.8), 7.60 – 7.45 (2 H, m), 7.22 (1 H, ddd, *J* 7.7, 4.9, 1.3), 7.19 (1 H, dd, *J* 8.2, 2.1), 4.49 (2 H, s), 1.65 (1 H, tt, *J* 8.0, 5.3), 1.06 – 0.94 (2 H, m), 0.58 – 0.32 (2 H, m); ^{13}**C NMR** (100 MHz; CDCl$_3$) δ 157.0, 149.4, 148.2, 141.9, 140.1, 136.6, 134.5, 133.8, 129.6, 127.3, 122.6, 121.5, 120.7, 104.3, 47.4, 7.3, 3.8; **HRMS** *m*/*z* (ESI$^+$) calcd. for $C_{18}H_{17}{}^{81}Br^{35}ClN_4$ [M+H]$^+$ requires 305.0299, found 305.0306; **LCMS** 3.04 min, 95%, *m/z* 305 [M+H]$^+$ (Method A).

***N*-(4-Chloro-3-(trifluoromethyl)benzyl)-4-cyclopropyl-3-(pyridine-2-yl)-1*H*-pyrazol-5-amine, 6aj**

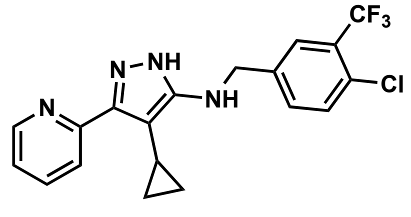

Isolated as an off-white solid (163 mg, 0.42 mmol, 42%); **mp.** 205 – 210 ºC; $\mathbf{R_f}$ 0.26 (1:1 EtOAc:*c*Hex); **FT-IR** ν_{max} (ATR)/cm^{-1} 3238, 1591, 1516, 1495, 1415, 1320, 1257, 1167, 1117, 1031, 788, 741, 638, 583; ^{1}**H NMR** (400 MHz; CDCl$_3$) δ 10.18 (1 H, s), 8.58 (1 H, dt, *J* 4.8,

1.5), 7.99 (1 H, dt, *J* 8.0, 1.1), 7.83 – 7.67 (2 H, m), 7.55 (1 H, dd, *J* 8.2, 2.3), 7.45 (1 H, d, *J* 8.2), 7.22 (1 H, ddd, *J* 7.7, 4.9, 1.3), 4.56 (2 H, d, *J* 6.0), 4.30 – 3.88 (1 H, m), 1.65 (1 H, tt, *J* 7.9, 5.2), 1.06 – 0.94 (2 H, m), 0.58 – 0.48 (2 H, m); **^{13}C NMR** (100 MHz; $CDCl_3$) δ 157.0, 149.5, 148.2, 140.1, 140.1, 136.6, 132.1, 131.6, 130.7 (q, *J* 2.0), 128.4 (q, *J* 31.1), 126.8 (q, *J* 5.3), 123.1 (d, *J* 273.3), 122.7, 121.5, 104.3, 47.4, 7.3, 3.8; **^{19}F {^{1}H}** NMR (376 MHz; $CDCl_3$) δ -62.52; **HRMS** *m/z* (ESI$^+$) calcd. for $C_{19}H_{17}{}^{35}ClF_3N_4$ [M+H]$^+$ requires 393.1088, found 393.1094; **LCMS** 3.05 min, 100%, *m/z* 393 [M+H]$^+$ (Method A).

4-Cyclopropyl-*N*-(3-fluoro-5-methoxybenzyl)-3-(pyridin-2-yl)-1*H*-pyrazol-5-amine, 6ak

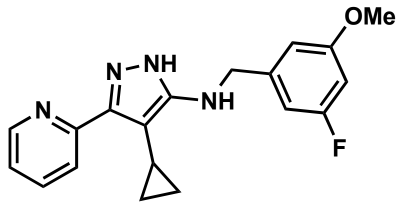

Isolated as a pale yellow solid (214 mg, 0.63 mmol, 63%); **mp.** 112- 114 ºC; **R$_f$** 0.10 (1:1 EtOAc:*c*Hex); **FT-IR** ν_{max} (ATR)/cm^{-1} 3435, 3167, 1929, 1593, 1301, 1146, 1053; **^{1}H NMR** (400 MHz; $CDCl_3$) δ 10.12 (1 H, s), 8.59 (1 H, ddd, *J* 4.9, 1.9, 1.0), 8.00 (1 H, dt, *J* 8.0, 1.1), 7.76 (1 H, td, *J* 7.8, 1.9), 7.21 (1 H, ddd, *J* 7.7, 4.8, 1.2), 6.90 – 6.64 (2 H, m), 6.51 (1 H, dt, *J* 10.7, 2.4), 4.51 (2 H, s), 3.99 (1 H, s), 3.78 (3 H, s), 1.65 (1 H, tt, *J* 8.0, 5.3), 1.10 – 0.90 (2 H, m), 0.63 – 0.39 (2 H, m); **^{13}C NMR** (100 MHz; $CDCl_3$) δ 163.9 (d, *J* 244.7), 161.1 (d, *J* 11.4), 157.3, 149.5, 148.3, 144.0 (d, *J* 8.8), 140.0, 136.6, 122.6, 121.5, 109.0 (d, *J* 2.6), 106.7 (d, *J* 22.0), 104.3, 100.3 (d, *J* 25.3), 55.6, 48.1, 48.1, 7.3, 3.8; **^{19}F {^{1}H}** NMR (376 MHz; $CDCl_3$) δ -

111.91; **HRMS** *m/z* (ESI$^+$) calcd. for $C_{19}H_{20}FN_4O$ [M+H]+ requires 339.1616, found 339.1616; **LCMS** 2.85 min, 97%, *m/z* 339 $[M+H]^+$ (Method A).

4-Cyclopropyl-*N*-(3-fluoro-4-methoxybenzyl)-3-(pyridin-2-yl)-1*H*-pyrazol-5-amine, 6al

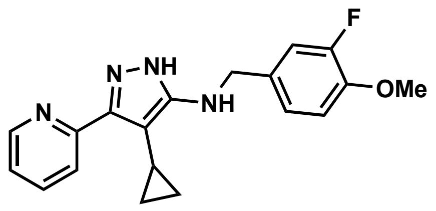

Isolated as a colourless solid (144 mg, 0.43 mmol, 43%); **mp.** 133 – 136 °C; $\mathbf{R_f}$ 0.21 (1:1 EtOAc:*c*Hex); **FT-IR** ν_{max} (ATR)/cm^{-1} 3364, 3264, 3083, 3044, 2984, 2906, 1507, 1273, 1219, 1180, 1103; ^{1}**H NMR** (400 MHz; $CDCl_3$) δ 8.62 – 8.57 (1 H, m), 8.00 (1 H, d, *J* 8.0), 7.77 (1 H, td, *J* 7.8, 1.8), 7.29 – 7.10 (3 H, m), 6.92 (1 H, t, *J* 8.5), 4.48 (2 H, s), 3.87 (3 H, s), 1.63 (1 H, tt, *J* 7.9, 5.2), 1.06 – 0.91 (2 H, m), 0.57 – 0.45 (2 H, m); ^{13}**C NMR** (100 MHz; $CDCl_3$) δ 156.9, 152.5 (d, *J* 245.6), 149.2, 148.1, 146.8 (d, *J* 10.7), 140.2, 136.8, 133.6 (d, *J* 5.8), 123.5 (d, *J* 3.5), 122.7, 121.7, 115.6 (d, *J* 18.5), 113.6 (d, *J* 2.1), 104.4, 56.5, 47.7, 7.3, 3.8; ^{19}**F{^{1}H} NMR** (376 MHz; $CDCl_3$) δ -135.2; **HRMS** *m/z* (ESI$^+$) calcd. for $C_{19}H_{17}FN_4O$ $[M+H]^+$ requires 339.1616 found 339.1611; **LCMS** 2.69 min, 100%, *m/z* 339 $[M+H]^+$ (Method A).

***N*-(3-Chloro-4-(trifluoromethyl)benzyl)-4-cyclopropyl-3-(pyridin-2-yl)-1*H*-pyrazol-5-amine, 6am**

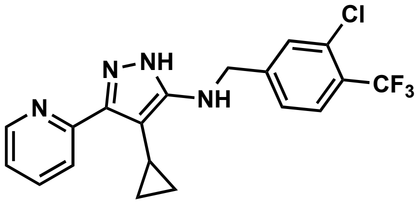

Isolated as a colourless solid (220 mg, 0.56 mmol, 56%); **mp.** 190 – 194 ºC; **R$_f$** 0.31 (1:1 EtOAc:*c*Hex); **FT-IR** ν_{max} (ATR)/cm^{-1} 3326, 1531, 1310, 1175, 1120, 1099, 1023, 823, 787, 703, 657; **^{1}H NMR** (400 MHz; CDCl$_3$) δ 10.26 (1 H, s), 8.59 (1 H, ddd, *J* 4.8, 1.8, 0.9), 8.00 (1 H, dt, *J* 8.0, 1.1), 7.76 (1 H, td, *J* 7.7, 1.8), 7.63 (1 H, d, *J* 8.2), 7.57 (1 H, dd, *J* 1.6, 0.8), 7.40 (1 H, ddq, *J* 8.2, 1.8, 0.8), 7.22 (1 H, ddd, *J* 7.5, 4.8, 1.1), 4.76 – 4.40 (2 H, m), 4.25 – 4.02 (1 H, m), 1.67 (2 H, tt, *J* 7.9, 5.2), 1.17 – 0.90 (2 H, m), 0.63 – 0.39 (2 H, m); **^{13}C NMR** (100 MHz; CDCl$_3$) δ 156.9, 149.5, 148.2, 146.8, 140.1, 136.6, 132.5 (q, *J* 1.8), 130.3, 127.8 (q, *J* 5.3), 126.9 (d, *J* 31.5), 125.6, 123.1 (d, *J* 272.5), 122.7, 121.5, 104.2, 47.3, 7.3, 3.8; **^{19}F {^{1}H}** NMR (376 MHz; CDCl$_3$) δ -62.29 **HRMS** *m/z* (ESI$^+$) calcd. for $C_{19}H_{17}{}^{35}ClF_3N_4$ [M+H]$^+$ requires 393.1088, found 393.1091; **LCMS** 3.05 min, 100%, *m/z* 393 [M+H]$^+$ (Method A).

4-Cyclopropyl-*N*-(2,3-dimethoxybenzyl)-3-(pyridin-2-yl)-1*H*-pyrazol-5-amine, 6an

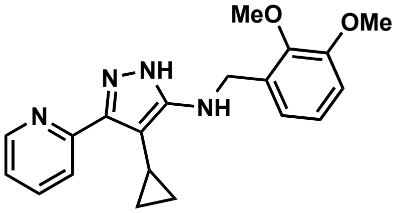

Isolated as a colourless solid (166 mg, 0.49 mmol, 49%); **mp.** 155 – 156 ºC; **R$_f$** 0.41 (1:1 EtOAc:*c*Hex); **FT-IR** ν_{max} (ATR)/cm^{-1} 3352, 2922, 2973, 1586, 1507, 1424, 1322; **^{1}H NMR**

(400 MHz; $CDCl_3$) δ 8.71 – 8.55 (1 H, m), 8.01 (1 H, d, *J* 8.0), 7.78 (1 H, td, *J* 7.9, 1.8), 7.28 – 7.22 (2 H, m), 7.02 (1 H, d, *J* 1.9), 6.97 (1 H, dd, *J* 8.2, 2.0), 6.85 (1 H, d, *J* 8.1), 4.49 (2 H, s), 3.88 – 3.86 (6 H, m), 1.63 (1 H, tt, *J* 7.9, 5.2), 1.07 – 0.91 (2 H, m), 0.59 – 0.45 (2 H, m); **^{13}C NMR** (100 MHz; $CDCl_3$) δ δ_C (101 MHz, $CDCl_3$) 156.8, 149.2, 149.2, 148.3, 147.9, 140.3, 136.9, 132.8, 122.8, 121.9, 120.1, 111.5, 111.3, 104.4, 56.1, 56.0, 48.6, 7.4, 3.8; **HRMS** *m/z* (ESI$^+$) calcd. for $C_{20}H_{23}N_4O_2$ [M+H]$^+$ requires 351.1816 found 351.1820; **LCMS** 2.74 min, 100%, *m/z* 351 [M+H]$^+$ (Method A).

4-Cyclopropyl-*N*-(4-methoxy-3-methylbenzyl)-3-(pyridin-2-yl)-1*H*-pyrazol-5-amine, 6ao

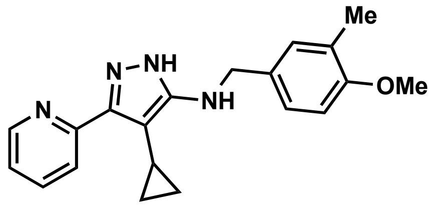

Isolated as a colourless solid (128 mg, 0.38 mmol, 35%); **mp.** 150 – 151 °C; **FT-IR** ν_{max} (ATR)/cm^{-1} 3379, 3230, 2986, 2831, 1589, 1519, 1497, 1464, 1445, 1429, 1391, 1329, 1293, 1256, 1225, 1185, 1145, 1134, 1091, 1073, 1052, 1029, 998, 970. 904, 893, 816, 788, 744, 732, 703; **^{1}H NMR** (300 MHz; $CDCl_3$) δ 8.58 (1 H, ddd, *J* 4.8, 1.8, 1.0), 7.99 (1 H, dt, *J* 8.0, 1.1), 7.75 (1 H, ddd, *J* 8.0, 7.5, 1.8), 7.26 – 7.16 (3 H, m), 6.91 – 6.71 (1 H, m), 4.42 (2 H, s), 3.83 (3 H, s), 2.23 (3 H, d, *J* 0.7), 1.63 (1 H, tt, *J* 8.0, 5.3), 0.99 – 0.85 (2 H, m), 0.58 – 0.40 (2 H, m); **^{13}C NMR** (75 MHz; $CDCl_3$) δ 157.8, 157.1, 149.4, 148.4, 139.9, 136.5, 132.1, 130.6, 126.8, 126.4, 122.5, 121.4, 110.1, 104.3, 55.5, 48.2, 16.4, 7.3, 3.9; **HRMS** *m/z* (ESI$^+$) calcd. for $C_{20}H_{23}N_4O$ [M+H]$^+$ requires 335.1867 found 335.1857 (Method A).

***N*-(3-Bromo-4-propoxybenzyl)-4-cyclopropyl-3-(pyridin-2-yl)-1*H*-pyrazol-5-amine, 6ap**

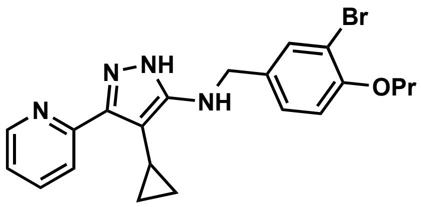

Isolated as a colourless solid (200 mg, 0.46 mmol, 42%); **mp.** 170 – 171 °C; **FT-IR** ν_{max} (ATR)/cm^{-1} 3401, 3142, 3074, 2971, 2938, 2869, 1595, 1499, 1481, 1468, 1433, 1407, 1388, 1338, 1291, 1280, 1259, 1143, 1108, 1094, 1057, 1045, 1004, 981, 893, 867, 828, 809, 787, 746, 730, 716, 672; **^{1}H NMR** (300 MHz; $CDCl_3$) δ 10.19 (1 H, s), 8.58 (1 H, ddd, *J* 4.9, 1.8, 1.0), 7.99 (1 H, dt, *J* 8.0, 1.1), 7.75 (1 H, td, *J* 7.8, 1.8), 7.61 (1 H, d, *J* 2.1), 7.31 (1 H, dd, *J* 8.3, 2.2), 7.21 (1 H, ddd, *J* 7.6, 4.9, 1.2), 6.85 (1 H, d, *J* 8.4), 4.44 (2 H, d, *J* 5.8), 3.98 (3 H, t, *J* 6.4), 1.93 – 1.76 (2 H, m), 1.63 (1 H, tt, *J* 8.0, 5.2), 1.07 (3 H, t, *J* 7.4), 1.02 – 0.93 (2 H, m), 0.55 – 0.46 (2 H, m); **^{13}C NMR** (75 MHz; $CDCl_3$) δ 157.5, 154.7, 149.5, 148.3, 136.6, 134.1, 132.8, 128.0, 122.6, 121.5, 113.4, 112.4, 104.3, 70.9, 47.5, 22.7, 10.7, 7.3, 3.8; **HRMS** *m/z* (ESI$^+$) calcd. for $C_{21}H_{24}{}^{79}BrN_4O$ $[M+H]^+$ requires 427.1128 found 417.1123. (Method X)

***N*-(3-Bromo-4-isopropoxybenzyl)-4-cyclopropyl-3-(pyridin-2-yl)-1*H*-pyrazol-5-amine, 6aq**

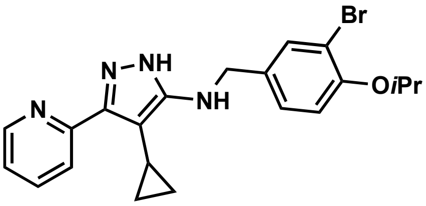

Isolated as a colourless solid (98 mg, 0.23 mmol, 45%); **mp.** 120 – 121 °C; **FT-IR** ν_{max} (ATR)/cm^{-1} 3325, 2977, 2927, 2852, 1592, 1491, 1463, 1430, 1405, 1376, 1336, 1280, 1252, 1181, 1139, 1110, 1042, 999, 972, 952, 898, 859, 788, 732, 714, 672; **^{1}H NMR** (300 MHz; $CDCl_3$) δ 8.61 (1 H, ddd, *J* 4.9, 1.8, 0.9), 7.99 (1 H, dt, *J* 8.1, 1.1), 7.75 (0 H, d, *J* 1.9), 7.62 (1

H, d, *J* 2.2), 7.30 (1 H, dd, *J* 8.4, 2.2), 7.20 (1 H, ddd, *J* 7.5, 4.8, 1.1), 6.87 (1 H, d, *J* 8.4), 4.70 – 4.32 (3 H, m), 1.63 (1 H, tt, *J* 8.0, 5.3), 1.37 (6 H, d, *J* 6.1), 1.08 – 0.83 (2 H, m), 0.59 – 0.39 (2 H, m); **^{13}C NMR** (75 MHz; $CDCl_3$) δ 157.3, 153.7, 149.4, 148.4, 139.9, 136.6, 134.6, 132.9, 127.9, 122.5, 121.6, 116.0, 113.8, 104.3, 72.4, 47.5, 22.2, 7.3, 3.8; **HRMS** *m/z* (ESI$^+$) calcd. for $C_{21}H_{24}{}^{79}BrN_4O$ $[M+H]^+$ requires 427.1128 found 427.1117 (Method A).

4-Cyclopropyl-*N*-(3,5-dichlorobenzyl)-3-(pyridin-2-yl)-1*H*-pyrazol-5-amine, 6ar

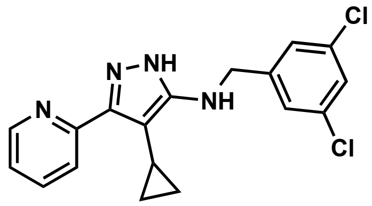

Isolated as a colourless solid (143 mg, 0.40 mmol, 40%); **mp.** 177 – 180 ºC; **R$_f$** 0.27 (1:1 EtOAc:*c*Hex); **FT-IR** ν_{max} (ATR)/cm^{-1} 3429, 3142, 3084, 3063, 2993, 2931, 2910, 1721, 1592, 1571, 1527, 1502, 1482; **^{1}H NMR** (400 MHz; $CDCl_3$) δ 10.29 (1 H, s), 8.59 (1 H, dd, *J* 5.9, 1.0), 8.00 (1 H, d, *J* 8.0), 7.76 (1 H, td, *J* 7.8, 1.9), 7.31 (2 H, d, *J* 2.0), 7.26 – 7.17 (2 H, m), 4.51 (2 H, d, *J* 6.3), 4.19 – 3.92 (1 H, m), 1.66 (1 H, ddd, *J* 8.0, 5.2, 2.8), 1.07 – 0.95 (2 H, m), 0.54 (2 H, dt, *J* 5.4, 2.9); **^{13}C NMR** (100 MHz; $CDCl_3$) δ 156.9, 149.5, 148.2, 144.4, 140.1, 136.6, 135.1, 127.2, 126.1, 122.6, 122.6, 121.5, 104.3, 47.4, 7.3, 3.8; **HRMS** *m/z* (ESI$^+$) calcd. for $C_{18}H_{17}N_4{}^{35}Cl_2$ $[M+H]^+$ requires 359.0825, found 359.0823; **LCMS** 3.06 min, 96%, *m/z* 359 $[M+H]^+$ (Method A).

4-Cyclopropyl-*N*-(3,5-dibromobenzyl)-3-(pyridin-2-yl)-1*H*-pyrazol-5-amine, 6as

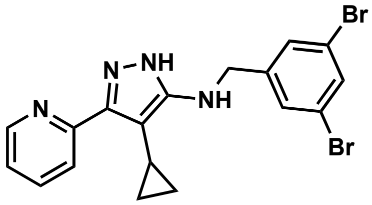

Isolated as a red solid (123 mg, 0.27 mmol, 27%); **mp.** 189 – 191 ºC; **R_f** 0.21 (1:1 EtOAc:*c*Hex); **FT-IR** ν_{max} (ATR)/cm^{-1} 3364, 1522, 1143, 845, 785, 731, 664, 535; **^{1}H NMR** (400 MHz; $CDCl_3$) δ 10.39 (1 H, s), 8.60 (1 H, ddd, *J* 4.8, 1.9, 1.1), 8.00 (1 H, dt, *J* 7.9, 1.1), 7.76 (1 H, td, *J* 7.8, 1.9), 7.52 (3 H, m), 7.22 (1 H, ddd, *J* 7.5, 4.8, 1.2), 4.50 (2 H, s), 4.10 (1 H, s), 1.66 (1 H, tt, *J* 8.0, 5.3), 1.07 – 0.92 (2 H, m), 0.64 – 0.33 (2 H, m); **^{13}C NMR** (100 MHz; $CDCl_3$) δ 156.9, 149.5, 148.2, 148.2, 145.0, 140.1, 136.6, 132.7, 129.5, 123.1, 122.6, 121.5, 104.3, 47.3, 7.4, 3.8; **HRMS** *m*/*z* (ESI$^+$) calcd. for $C_{18}H_{17}{}^{81}Br_2N_4$ [M+H]$^+$ requires 450.9774, found 450.9772; **LCMS** 3.11 min, 95%, *m/z* 450 [M+H]$^+$ (Method A).

***N*-(3-Bromo-5-chlorobenzyl)-4-cyclopropyl-3-(pyridin-2-yl)-1*H*-pyrazol-5-amine, 6at**

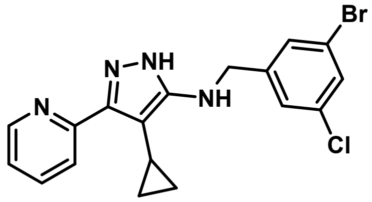

Isolated as a pale yellow solid (109 mg, 0.27 mmol, 27% yield); **mp.** 188 – 189 ºC; **R_f** 0.15 (1:1 EtOAc:*c*Hex); **FT-IR** ν_{max} (ATR)/cm^{-1} 3142, 1590, 1566, 1527, 1502, 1481, 1426, 1194; **^{1}H NMR** (400 MHz; $CDCl_3$) δ 10.17 (1 H, s), 8.58 (1 H, ddd, *J* 4.9, 1.9, 1.0), 8.00 (1 H, dt, *J* 8.0, 1.1), 7.76 (1 H, td, *J* 7.8, 1.9), 7.46 (1 H, tt, *J* 1.5, 0.8), 7.39 (1 H, t, *J* 1.9), 7.36 (1 H, tq, *J* 1.5,

0.8), 7.22 (1 H, ddd, *J* 7.5, 4.8, 1.2), 4.57 – 4.43 (2 H, m), 4.15 – 4.01 (1 H, m), 1.66 (1 H, tt, *J* 7.9, 5.3), 1.08 – 0.92 (2 H, m), 0.61 – 0.41 (2 H, m); **^{13}C NMR** (100 MHz; $CDCl_3$) δ 157.0, 149.5, 148.2, 144.7, 140.1, 136.6, 135.2, 130.0, 129.0, 126.6, 122.9, 122.7, 121.5, 104.2, 47.4, 7.3, 3.8; **HRMS** *m/z* (ESI$^+$) calcd. for $C_{18}H_{17}{}^{81}Br^{35}ClN_4$ [M+H]$^+$ requires 405.0299, found 405.0300; **LCMS** 3.08 min, 95%, *m/z* 405 [M+H]$^+$ (Method A).

***N*-(3-Bromo-5-(trifluoromethyl)benzyl)-4-cyclopropyl-3-(pyridin-2-yl)-1*H*-pyrazol-5-amine, 6au**

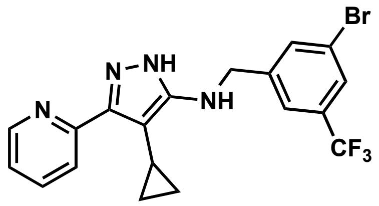

Isolated as a beige solid (150 mg, 0.34 mmol, 34%); **mp.** 183 – 184 °C; **R$_f$** 0.36 (1:1 EtOAc:*c*Hex); **FT-IR** ν_{max} (ATR)/cm^{-1} 3354, 1588, 1418, 1343, 1163, 1117; **^{1}H NMR** (400 MHz; $CDCl_3$) δ 10.15 (1 H, s), 8.58 (1 H, ddd, *J* 4.9, 1.9, 1.0), 8.00 (1 H, dt, *J* 7.9, 1.1), 7.82 – 7.71 (2 H, m), 7.63 (2 H, dtd, *J* 9.3, 1.7, 0.8), 4.58 (2 H, s), 4.25 – 3.95 (1 H, m), 1.66 (1 H, tt, *J* 8.0, 5.3), 1.08 – 0.92 (2 H, m), 0.64 – 0.44 (2 H, m); **^{13}C NMR** (100 MHz; $CDCl_3$) δ 156.9, 149.5, 148.2, 144.3, 140.1, 136.6, 134.1, 132.5 (q, *J* 32.6), 127.1 (q, *J* 4.0), 123.4 (q, *J* 272.9), 123.2 (q, *J* 3.7), 122.9, 122.7, 121.5, 104.3, 47.5, 7.3, 3.8; **^{19}F {^{1}H}** NMR (376 MHz; $CDCl_3$) δ -62.70; **HRMS** *m/z* (ESI$^+$) calcd. for $C_{19}H_{17}{}^{79}BrF_3N_4$ [M+H]$^+$ requires 437.0583, found 437.0586; **LCMS** 3.13 min, 95%, *m/z* 437 [M+H]$^+$ (Method A).

4-Cyclopropyl-*N*-(3,5-difluorobenzyl)-3-(pyridin-2-yl)-1*H*-pyrazol-5-amine, 6av

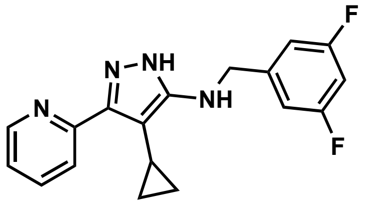

Isolated as a white solid with (32 mg, 0.10 mmol, 30%) **FT-IR** ν_{max} (ATR)/cm^{-1} 3340, 3055, 1591, 1520, 1456, 1301,1105, 844; ^{1}**H NMR** (500 MHz, $CDCl_3$) δ 9.04 – 8.42 (1H, m), 8.00 (1H, d, *J* 8.0), 7.76 (1H, td, *J* 7.8, 1.8), 7.22 (1H, ddd, *J* 7.6, 4.9, 1.2), 6.95 (2H, h, *J* 4.4), 6.67 (1H, tt, *J* 9.0, 2.4), 4.55 (2H, s), 1.66 (1H, tt, *J* 8.2, 5.3), 1.00 (2H, dt, *J* 10.0, 3.0), 0.52 (2H, dt, *J* 6.0, 3.0); ^{13}**C NMR** (126 MHz, $CDCl_3$) δ 163.2 (dd, *J* 248.1, 12.7), 156.9, 149.5, 148.2, 145.1 (t, *J* 8.5), 140.1, 136.6, 122.6, 121.6, 110.2 (dd, *J* 25.4, 13.8), 104.2, 102.3 (t, *J* 25.4), 47.6 (t, *J* 2.2), 7.3, 3.8; **LCMS** 3.54 min, 100%, *m/z* 327.1 [M+H]$^+$. (Method A)

4-Cyclopropyl-3-(pyridin-2-yl)-*N*-(3,4,5-trimethoxybenzyl)-1*H*-pyrazol-5-amine, 6aw

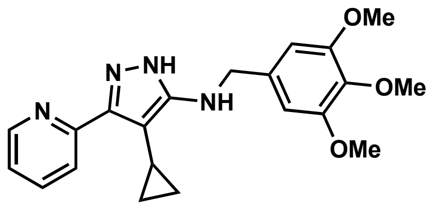

Isolated as a white solid with (30 mg, 0.08 mmol, 30%) **FT-IR** ν_{max} (ATR)/cm^{-1} 3351, 3056, 1590, 1506,1454, 1232, 1122, 1003; ^{1}**H NMR** (500 MHz, $CDCl_3$) δ 8.60 (1H, d, *J* 4.4), 7.99 (1H, d, *J* 8.0), 7.78 (1H, td, *J* 7.8, 1.7), 7.26 – 7.22 (1H, m), 6.68 (2H, s), 4.43 (2H, s), 3.86 (6H, s), 3.85 (3H, s), 1.70 – 1.58 (1H, m), 1.04 – 0.95 (2H, m), 0.52 – 0.43 (2H, m); ^{13}**C NMR** (126 MHz, $CDCl_3$) δ 175.5, 156.6, 153.5, 149.3, 148.0, 140.1, 136.5, 135.7, 122.5, 121.8,

104.8, 104.1, 61.0, 56.1, 48.9, 20.8, 7.0, 3.8; **LCMS** 3.00 min, 100%, *m/z* 381 [M+H]$^+$. (Method B)

4-Cyclopropyl-*N*-((3-fluoropyridin-2-yl)methyl)-3-(pyridin-2-yl)-1*H*-pyrazol-5-amine, 6ax

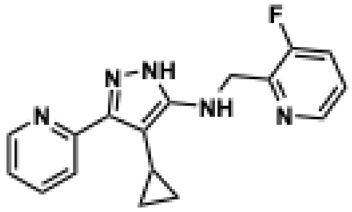

Isolated after recrystallization in hexane /ethyl acetate to yield an off-white solid (11 mg, 0.04 mmol, 7%); **mp.** 130-134 ºC; **R$_f$** 0.40 (100% Ethyl Acetate); ^{1}**H NMR** (500 M $CDCl_3$) δ 10.51 (1 H, s), 8.60 (1 H, ddd, *J* 4.8, 1.9, 1.0), 8.42 (1 H, dt, *J* 4.8, 1.4), 8.02 (1 H, dt, *J* 8.0, 1.1), 7.75 (1 H, td, *J* 7.8, 1.8), 7.36 (1 H, ddd, *J* 9.5, 8.2, 1.4), 7.30 – 7.12 (2 H, m), 5.15 (1 H, t, *J* 5.7), 4.74 (2 H, dd, *J* 5.5, 1.8), 1.71 (1 H, tt, *J* 8.0, 5.2), 1.32 – 1.24 (1 H, m), 1.07 – 1.00 (2 H, m), 0.62 – 0.56 (2 H, m); ^{13}**C NMR** (126 MHz; $CDCl_3$) δ 157.5, 157.3 (d, *J* 257.3), 150.8 – 144.8 (m), 144.8 (d, *J* 5.4) 139.9, 136.5, 123.2 (d, *J* 3.5), 124.0 – 121.1 (m), 121.5, 104.7, 43.4, 7.3, 4.0. (Method A)

4-Cyclopropyl-3-(pyridin-2-yl)-*N*-((3-(trifluoromethyl)pyridin-2-yl)methyl)-1*H*-pyrazol-5-amine, 6ay

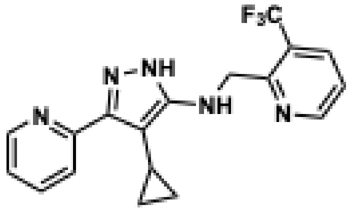

Isolated as an off-white crystal after recrystallisation from EtOAc/Hex (82 mg, 0.23 mmol, 46%); **mp.** 163 ºC; **R$_f$** 0.41 (100% EtOAc); ^{1}**H NMR** (500 MHz; $CDCl_3$) δ 10.05 (1 H, s), 8.78 (1

H, ddd, *J* 4.9, 1.8, 0.8), 8.57 (1 H, ddd, *J* 4.8, 1.8, 1.0), 8.03 (1 H, dt, *J* 8.0, 1.1), 7.97 (1 H, ddd, *J* 7.8, 1.7, 0.7), 7.75 (1 H, ddd, *J* 8.0, 7.5, 1.8), 7.34 (1 H, ddq, *J* 7.9, 4.8, 0.8), 7.30 – 7.17 (1 H, m), 5.48 (1 H, s), 4.84 (2 H, d, *J* 4.7), 1.74 (1 H, tt, *J* 8.1, 5.3), 1.11 – 1.01 (2 H, m), 0.69 – 0.60 (2 H, m); **^{13}C NMR** (125 MHz; $CDCl_3$) δ 158.3, 157.5, 156.4, 151.6, 149.3, 148.3, 136.4, 134.1, 134.0, 122.3, 121.5, 121.3, 104.6, 46.2, 46.1, 7.1, 3.9. (Method A)

4-Cyclopropyl-3-(pyridin-2-yl)-*N*-(thiophen-2-ylmethyl)-1*H*-pyrazol-5-amine, 6az

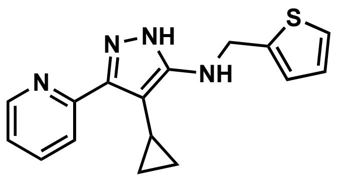

Isolated as a colourless gum (140 mg, 0.47 mmol, 47%); **R$_f$** 0.25 (1:1 EtOAc:*c*Hex); **FT-IR** ν_{max} (ATR)/cm^{-1} 3190, 1591, 1498, 1429, 1296, 1149, 1053, 1000, 789, 696, 625; **^{1}H NMR** (400 MHz; $CDCl_3$) δ 8.59 (1 H, d, *J* 5.3), 7.99 (1 H, d, *J* 8.0), 7.75 (1 H, td, *J* 7.8, 1.8), 7.22 (2 H, dd, *J* 7.8, 4.8), 7.05 (1 H, d, *J* 3.8), 6.96 (1 H, dd, *J* 5.1, 3.5), 4.72 (2 H, s), 4.04 (1 H, s), 1.67 – 1.58 (1 H, m), 1.01 – 0.92 (2 H, m), 0.51 (2 H, dt, *J* 5.4, 2.9); **^{13}C NMR** (100 MHz; $CDCl_3$) δ 157.1, 149.5, 148.3, 143.9, 140.0, 136.6, 126.8, 125.2, 124.6, 122.6, 121.5, 104.7, 43.6, 7.3, 3.8; **HRMS** *m/z* (ESI$^+$) calcd. for $C_{16}H_{17}N_4S$ [M+H]$^+$ requires 297.1168, found 297.1168; **LCMS** 1.92 min, 95%, *m/z* 297 [M+H]$^+$ (Method A).

***N*-((4-Bromothiophen-2-yl)methyl)-4-cyclopropyl-3-(pyridin-2-yl)-1*H*-pyrazol-5-amine, 6ba**

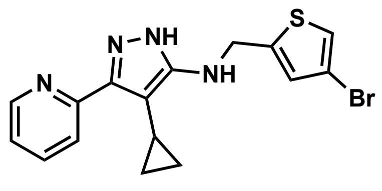

Isolated as a yellow solid (131 mg, 0.35 mmol, 35%); **mp.** 140 – 142 ºC; **R$_f$** 0.60 (1:1 EtOAc:*c*Hex); **FT-IR** ν$_{max}$ (ATR)/cm^{-1} 3361, 3103, 1518, 1473, 1360, 740, 542; ^{1}**H NMR** (400 MHz; CDCl$_3$) δ 10.25 (1 H, s), 8.59 (1 H, ddd, *J* 4.8, 1.8, 0.9), 7.99 (1 H, dt, *J* 8.0, 1.1), 7.76 (1 H, td, *J* 7.8, 1.9), 7.22 (1 H, ddd, *J* 7.7, 4.9, 1.3), 7.09 (1 H, d, *J* 1.4), 6.96 (1 H, q, *J* 1.1), 4.68 (2 H, s), 4.07 (1 H, s), 1.63 (1 H, tt, *J* 8.0, 5.3), 1.05 – 0.86 (2 H, m), 0.68 – 0.37 (2 H, m); ^{13}**C NMR** (100 MHz; CDCl$_3$) δ 156.7, 149.5, 148.2, 145.6, 140.1, 136.6, 127.6, 122.7, 121.7, 121.4, 109.2, 104.7, 43.3, 7.3, 3.8; **HRMS** *m/z* (ESI$^+$) calcd. for $C_{16}H_{16}BrN_4S$ [M+H]$^+$ requires 375.0274, found 375.0278; **LCMS** 2.86 min, 98%, *m/z* 375 [M+H]$^+$ (Method A).

***N*-((2-Bromothiazol-5-yl)methyl)-4-cyclopropyl-3-(pyridin-2-yl)-1*H*-pyrazol-5-amine, 6bb**

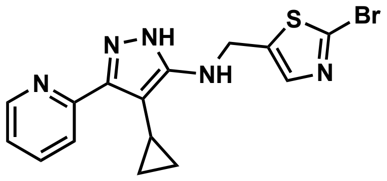

Isolated as a colourless solid (21 mg, 0.06 mmol, 6%); **mp.** 145 – 149 ºC; **R$_f$** 0.58 (1:1 EtOAc:*c*Hex); **FT-IR** ν$_{max}$ (ATR)/cm^{-1} 3295, 1592, 1512, 1429, 1251, 1152, 999, 787, 742, 625, 573; ^{1}**H NMR** (400 MHz; CDCl$_3$) δ 8.63 (1 H, d, *J* 4.8), 8.01 (1 H, d, *J* 8.0), 7.78 (1 H, td, *J* 7.8, 1.8), 7.48 (1 H, s), 7.28 – 7.18 (1 H, m), 4.70 (2 H, s), 1.61 (1 H, tt, *J* 8.0, 5.3), 1.04 – 0.92 (2 H, m), 0.47 (2 H, dt, *J* 5.9, 3.0); ^{13}**C NMR** (100 MHz; CDCl$_3$) δ 155.9, 149.3, 148.0,

142.3, 140.3, 140.3, 136.9, 135.9, 122.8, 121.7, 104.8, 40.9, 7.3, 3.7; **HRMS** *m/z* (ESI^+) calcd. for $C_{15}H_{15}{}^{79}BrN_5S$ $[M+H]^+$ requires 376.0226, found 376.0216; **LCMS** 2.74 min, 100%, *m/z* 376 $[M+H]^+$ (Method A).

4-Cyclopropyl-*N*-((5-(difluoromethyl)oxazol-4-yl)methyl)-3-(pyridin-2-yl)-1*H*-pyrazol-5-amine, 6bc

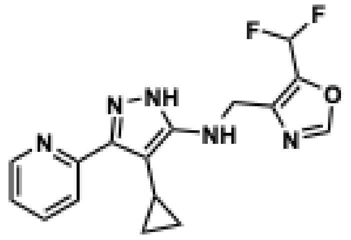

Isolated after recrystallization from hexane/ethyl acetate to yield a crystal (68 mg, 0.21 mmol, 41%); **mp.** 118-128 °C ; $\mathbf{R_f}$ 0.60 (8:2 Ethyl Acetate:Hexane); **^{1}H NMR** (500 MHz; $CDCl_3$) δ 8.61 (1 H, ddd, *J* 4.9, 1.8, 0.9), 8.00 – 7.95 (1 H, m), 7.89 (1 H, d, *J* 1.2), 7.75 (1 H, td, *J* 7.8, 1.8), 7.24 – 7.18 (1 H, m), 7.09 (1 H, s), 5.29 (2 H, s), 4.53 (1 H, s), 2.04 (1 H, s), 1.61 (1 H, tt, *J* 8.0, 5.3), 1.25 (1 H, t, *J* 7.1), 1.02 – 0.92 (3 H, m), 0.51 – 0.44 (2 H, m); **^{13}C NMR** (126 MHz; $CDCl_3$) δ (*rotamers*) 156.2, 151.4, 149.3, 148.2, 140.0, 140.0, 139.8, 139.6, 139.3, 139.2, 139.2, 136.5, 122.5, 121.5, 109.3, 107.4, 105.5, 104.7, 39.2, 14.2, 8.1, 7.7, 7.1, 3.6. (Method A)

4-Cyclopropyl-*N*-((3-methoxypyridin-2-yl)methyl)-3-(pyridin-2-yl)-1*H*-pyrazol-5-amine, 6bd

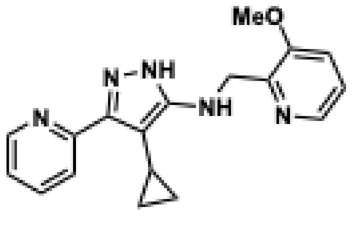

Isolated after recrystallization from hexane /ethyl acetate to yield a light brown solid (86 mg, 0.27 mmol, 54%); **mp.** 132-136 ºC ; **R$_f$** 0.07 (8:2 EtOAc:hexane); **^{1}H NMR** (500 MHz; $CDCl_3$) δ 10.07 (1 H, s), 8.60 – 8.54 (1 H, m), 8.19 (1 H, dd, *J* 4.7, 1.4), 8.03 (1 H, dt, *J* 7.9, 1.1), 7.75 (1 H, td, *J* 7.8, 1.8), 7.23 – 7.14 (2 H, m), 7.13 (1 H, dd, *J* 8.2, 1.4), 5.38 (1 H, s), 4.66 (2 H, s), 3.87 (3 H, s), 1.73 (1 H, tt, *J* 8.0, 5.3), 1.11 – 1.00 (2 H, m), 0.66 – 0.55 (2 H, m); **^{13}C NMR** (126 MHz; $CDCl_3$) δ 153.2, 149.4, 148.6, 148.2, 140.3, 136.5, 122.4, 122.4, 121.5, 116.5, 104.6, 55.3, 44.4, 7.3, 4.0. (Method A)

4-Cyclopropyl-*N*-((1-propyl-1*H*-indol-5-yl)methyl)-3-(pyridin-2-yl)-1*H*-pyrazol-5-amine, 6be

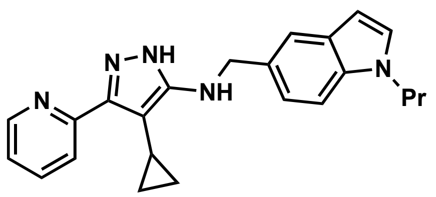

Isolated as a colourless solid (112 mg, 0.30 mmol, 71%); **mp.** 65 – 67 ºC; **FT-IR** ν_{max} (ATR)/cm^{-1} 3189, 3076, 2973, 2928, 2866, 1591, 1518, 1497, 1480, 1466, 1429, 1409, 1364, 1353, 1317, 1293, 1222, 1138, 1094, 1053, 1017, 1000, 973, 883, 822, 788, 760, 716, 668; **^{1}H NMR** (300 MHz; $CDCl_3$) δ 8.58 (1 H, ddd, *J* 4.8, 1.8, 0.9), 8.00 (1 H, dt, *J* 8.0, 1.1), 7.75 (1 H, td, *J* 7.7, 1.8), 7.69 (1 H, h, *J* 0.7), 7.34 – 7.31 (2 H, m), 7.21 (1 H, ddd, *J* 7.6, 4.9, 1.2), 7.11 (1 H, d, *J* 3.1), 6.47 (1 H, dd, *J* 3.1, 0.7), 4.60 (2 H, s), 4.09 (2 H, t, *J* 7.1), 1.88 (2 H, h, *J* 7.3), 1.61 (1 H, tt, *J* 8.1, 5.3), 1.00 – 0.85 (5 H, m), 0.59 – 0.44 (2 H, m); **^{13}C NMR** (75 MHz; $CDCl_3$) δ 158.1, 149.4, 148.5, 139.9, 136.5, 135.6, 131.1, 128.8, 128.4, 122.5, 122.1, 121.4, 120.4, 109.6, 104.3, 100.9, 49.3, 48.3, 23.7, 11.7, 7.4, 3.9; **HRMS** *m/z* (ESI$^+$) calcd. for $C_{23}H_{26}N_5$ $[M+H]^+$ requires 372.2183 found 372.2171 (Method B).

4-Cyclopropyl-*N*-((1-isopropyl-1*H*-indol-5-yl)methyl)-3-(pyridin-2-yl)-1*H*-pyrazol-5-amine, 6bf

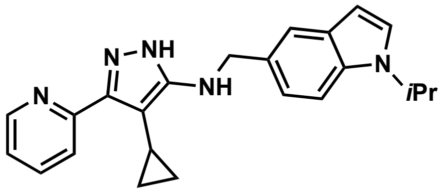

Isolated as a colourless solid (112 mg, 0.30 mmol, 71%); **mp.** 65 – 67 ºC; **FT-IR** ν_{max} (ATR)/cm^{-1} 3189, 3076, 2973, 2928, 2866, 1591, 1518, 1497, 1480, 1466, 1429, 1409, 1364, 1353, 1317, 1293, 1222, 1138, 1094, 1053, 1017, 1000, 973, 883, 822, 788, 760, 716, 668; **^{1}H NMR** (300 MHz; $CDCl_3$) δ 10.09 (1 H, s), 8.58 (1 H, ddd, *J* 4.8, 1.8, 1.0), 8.00 (1 H, ddt, *J* 8.0, 2.3, 1.1), 7.75 (1 H, tdd, *J* 7.9, 3.5, 1.8), 7.71 – 7.66 (1 H, m), 7.41 – 7.29 (2 H, m), 7.25 – 7.17 (2 H, m), 6.51 (1 H, dd, *J* 3.3, 0.8), 4.68 (1 H, hept, *J* 6.7), 4.60 (2 H, d, *J* 5.8), 3.95 (1 H, t, *J* 5.8), 1.73 – 1.47 (7 H, m), 1.07 – 0.87 (2 H, m), 0.60 – 0.44 (2 H, m); **^{13}C NMR** (75.5 MHz; $CDCl_3$) δ 158.1, 149.4, 139.9, 136.5, 135.0, 131.2, 128.8, 124.1, 122.5, 122.0, 121.4, 120.5, 113.8, 109.7, 104.3, 101.3, 49.3, 47.3, 22.9, 7.4, 3.9; **HRMS** *m*/*z* (ESI$^+$) calcd. for $C_{21}H_{22}N_5$ $[M+H]^+$ requires 372.2183 found 372.1276 (Method B).

***N*-Cyclopentyl-4-cyclopropyl-3-(pyridin-2-yl)-1*H*-pyrazol-5-amine, 6bg**

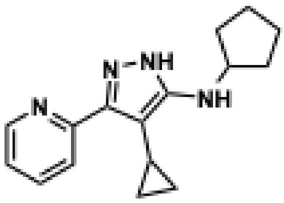

Isolated as a tan soft solid (190 mg, 0.71 mmol, 69%); **R$_f$** 0.14 (Ethyl Acetate); **^{1}H NMR** (500 MHz; $CDCl_3$) δ 9.98 (1 H, s), 8.59 – 8.54 (1 H, m), 7.97 (1 H, dd, *J* 8.0, 1.1), 7.74 (1 H, td, *J*

7.8, 1.8), 7.23 – 7.17 (1 H, m), 4.06 (1 H, t, *J* 6.5), 3.64 (1 H, s), 2.14 – 2.01 (2 H, m), 1.75 (2 H, q, *J* 6.5, 5.6), 1.63 (2 H, qd, *J* 8.2, 7.8, 3.9), 1.52 (2 H, dddd, *J* 13.3, 7.9, 6.0, 1.8), 1.02 – 0.92 (2 H, m), 0.54 – 0.44 (2 H, m); **^{13}C NMR** (126 MHz; $CDCl_3$) δ 149.4, 148.4, 136.5, 122.5, 121.4, 105.9, 55.7, 34.0, 24.0, 7.3, 3.9. (Method A)

***N*-Cyclohexyl-4-cyclopropyl-3-(pyridin-2-yl)-1*H*-pyrazol-5-amine, 6bh**

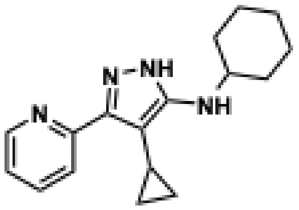

Isolated as a tan waxy solid (179 mg, 0.63 mmol, 63%); **R_f** 0.63 (EtOAc); **^{1}H NMR** (500 MHz; $CDCl_3$) δ 8.57 (1 H, ddd, *J* 4.9, 1.9, 1.0), 7.97 (1 H, dt, *J* 8.0, 1.1), 7.74 (1 H, td, *J* 7.8, 1.8), 7.20 (1 H, ddd, *J* 7.6, 4.8, 1.1), 3.54 (1 H, tt, *J* 10.3, 3.8), 2.17 – 2.10 (2 H, m), 1.76 (2 H, dt, *J* 14.0, 4.1), 1.62 (2 H, qt, *J* 8.0, 4.7), 1.42 (2 H, dtt, *J* 13.2, 11.8, 3.5), 1.29 – 1.15 (4 H, m), 1.02 – 0.95 (2 H, m), 0.52 – 0.46 (2 H, m); **^{13}C NMR** (126 MHz; $CDCl_3$) δ157.1, 149.3, 148.3, 139.6, 136.3, 122.3, 121.3, 104.3, 34.2, 26.1, 25.1, 7.2, 3.7. (Method A)

***N*-(Cyclopentylmethyl)-4-cyclopropyl-3-(pyridin-2-yl)-1*H*-pyrazol-5-amine, 6bi**

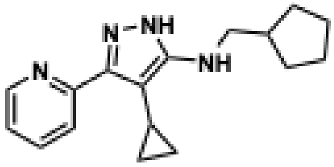

Isolated as an off-white solid (23 mg, 0.08 mmol, 34%); **mp.** 110 ºC; **R_f** 0.22 (9:1 CH_2Cl_2:MeOH); **^{1}H NMR** (500 MHz, $CDCl_3$) δ 8.59 (1 H, ddd, *J* 4.8, 1.8, 1.0), 7.98 (1 H, dt, *J* 8.0, 1.1 Hz), 7.74 (1 H, td, *J* 7.8, 1.9 Hz), 7.20 (1 H, ddd, *J* 7.6, 4.8, 1.2), 3.26 (2 H, d, *J* 7.3), 2.24 (1 H, hept, *J* 7.6), 1.90 – 1.77 (m, 2 H), 1.71 – 1.50 (4 H, m,), 1.38 – 1.29 (1 H, m,), 1.33 – 1.24 (1 H, m,), 1.03 – 0.93 (2 H, m,), 0.56 – 0.44 (2 H, m,); **^{13}C NMR** (126 MHz, $CDCl_3$) δ 157.9, 149.3, 148.3, 139.7, 136.4, 122.3, 121.4, 104.0, 82.6, 77.3, 77.0, 76.8, 49.7, 39.9, 30.6, 25.4, 7.1, 3.7. (Method A)

***N*-(Cyclohexylmethyl)-4-cyclopropyl-3-(pyridin-2-yl)-1*H*-pyrazol-5-amine, 6bj**

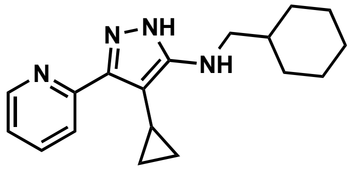

Isolated as an off-white solid (50 mg, 0.17 mmol, 14%); **mp.** 84 – 86 ºC; **R_f** 0.22 (9:1 CH_2Cl_2:MeOH); **^{1}H NMR** (500 MHz; $CDCl_3$) δ 10.31 (1 H, s), 8.59 (1 H, ddd, *J* 4.8, 1.9, 0.9), 7.97 (1 H, dt, *J* 8.0, 1.1), 7.73 (1 H, td, *J* 7.8, 1.8), 7.18 (1 H, ddd, *J* 7.6, 4.8, 1.2), 3.71 (1 H, *br* s), 3.18 (2 H, d, *J* 6.8), 1.88 – 1.83 (2 H, m), 1.74 (2 H, dt, *J* 11.7, 3.0), 1.71 – 1.59 (4 H, m), 1.34 – 1.15 (4 H, m), 1.06 – 0.94 (4 H, m), 0.52 – 0.41 (2 H, m); **^{13}C NMR** (126 MHz; $CDCl_3$) δ 158.0, 149.3, 148.3, 139.7, 136.4, 122.4, 121.3, 103.9, 50.8, 37.9, 31.2, 26.7, 26.1, 7.1, 3.7; **LRMS** *m*/*z* (ESI$^+$) 297; **LCMS** 100%, *m/z* 297 $[M+H]^+$ (Method A).

4-Cyclopropyl-3-(pyridin-2-yl)-*N*-((tetrahydro-2*H*-pyran-4-yl)methyl)-1*H*-pyrazol-5-amine, 6bk

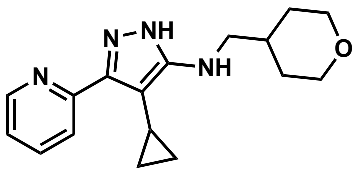

Isolated as an off-white solid (28 mg, 0.09 mmol, 11%); **mp.** 150 – 152 ºC; **R$_f$** 0.15 (EtOAc); **^{1}H NMR** (500 MHz; CDCl$_3$) δ 8.59 (1 H, d, *J* 4.8), 7.97 (1 H, d, *J* 7.9), 7.75 (1 H, s), 7.21 (1 H, d, *J* 2.8), 6.19 (1 H, s), 4.06 – 3.93 (2 H, m), 3.41 (2 H, td, *J* 11.8, 2.2), 3.26 (2 H, d, *J* 6.8), 2.04 – 1.87 (1 H, m), 1.73 (2 H, dd, *J* 13.0, 3.6), 1.62 (1 H, s), 1.38 (2 H, dd, *J* 12.8, 4.5), 1.31 – 1.19 (1 H, m), 0.98 (2 H, dd, *J* 8.0, 2.2), 0.48 (2 H, dd, *J* 5.4, 1.9); **^{13}C NMR** (126 MHz; CDCl$_3$) δ 157.2, 148.2, 140.0, 122.5, 121.5, 103.9, 67.9, 50.2, 35.1, 31.0, 7.2, 3.7; **LRMS** *m/z* (ESI$^+$) 299; **LCMS** 60%, *m/z* 299 [M+H]$^+$ (Method A).

***N*-(Cycloheptylmethyl)-4-cyclopropyl-3-(pyridin-2-yl)-1*H*-pyrazol-5-amine, 6bl**

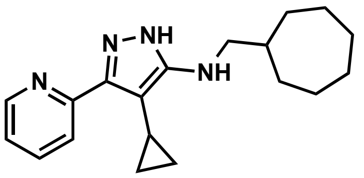

Isolated as a yellow oil (47 mg, 0.15 mmol, 19%); **R$_f$** 0.56 (7:1 CH_2Cl_2:MeOH); **^{1}H NMR** (500 MHz; CDCl$_3$) δ 8.57 (1 H, ddd, *J* 4.9, 1.8, 1.0), 7.98 (1 H, d, *J* 8.0), 7.74 (1 H, td, *J* 7.8, 1.9), 7.20 (1 H, ddd, *J* 7.6, 4.8, 1.2), 3.17 (2 H, d, *J* 6.5), 1.64 – 1.58 (4 H, m), 1.51 (5 H, dd, *J* 13.7, 10.3), 1.26 (5 H, d, *J* 2.1), 0.99 (2 H, dd, *J* 8.0, 2.0), 0.51 (2 H, dd, *J* 5.3, 2.0); **^{13}C NMR** (126

MHz; $CDCl_3$) δ 157.7, 149.3, 148.2, 139.8, 136.4, 122.4, 121.4, 103.9, 51.0, 39.6, 32.4, 28.6, 26.5, 7.1, 3.7; **LRMS** *m*/*z* (ESI$^+$) 311; **LCMS** 100%, *m/z* 311 [M+H]$^+$ (Method A).

***N*-Benzyl-3-(pyridin-2-yl)-1*H*-pyrazol-5-amine, 8a.**

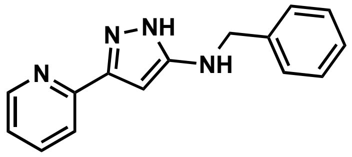

Isolated as a beige solid (94 mg, 0.38 mmol, 56%); **DSC** 137 ºC (solvates 89 ºC); **FT-IR** ν_{max} (ATR)/cm^{-1} 3197, 1597, 1565, 1504, 1409; **^{1}H NMR** (500 MHz; DMSO-*d*$_6$) δ 8.64 (1 H, s), 7.93 (2 H, s), 7.46 – 7.31 (6 H, m), 6.71 – 6.69 (1 H, m), 6.28 (1 H, s), 4.43 (2 H, d, *J* 5.0); **HRMS** *m*/*z* (ESI$^+$) calcd. for $C_{15}H_{15}N_4$ [M+H]$^+$ requires 251.1291, found 251.1281. (Method B)

***N*-(4-Methoxybenzyl)-3-(pyridin-2-yl)-1*H*-pyrazol-5-amine, 8b.**

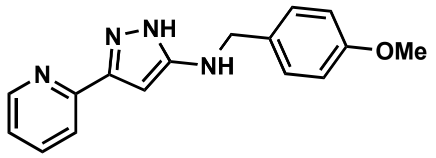

Isolated as a brown solid (112 mg, 0.40 mmol, 60%); **DSC** 131 ºC (solvates 101 ºC); **FT-IR** ν_{max} (ATR)/cm^{-1} 3145, 2925, 2597, 1508, 1242; **^{1}H NMR** (400 MHz; CD_3OD) δ 8.50 (1 H, s), 7.82 – 7.70 (2 H, m), 7.30 – 7.28 (3 H, m), 6.84 (2 H, d, *J* 8.4), 6.07 (1H, s, C*H*), 4.25 (2 H, s), 3.73 (3H, s); **HRMS** *m*/*z* (ESI$^+$) calcd. for $C_{16}H_{17}N_4O$ [M+H]$^+$ requires 281.1397, found 281.1383. (Method B)

***N*-(2-Fluorobenzyl)-3-(pyridin-2-yl)-1*H*-pyrazol-5-amine, 8c**

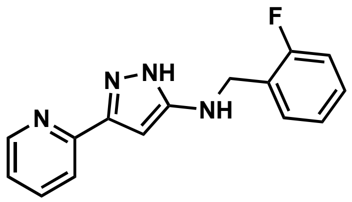

Isolated as a yellow solid (96 mg, 0.36 mmol, 53%); **DSC** 122 ºC; **FT-IR** ν_{max} (ATR)/cm^{-1} 3368, 3140, 3048, 2909, 1578, 1539; ^{1}**H NMR** (400 MHz; CD_3OD) δ 8.49 (1 H, d, *J* 4.0), 7.82 – 7.28 (2 H, m), 7.46 – 7.28 (3 H, m), 7.01 (2 H, t, *J* 6.0), 6.06 (1H, s, C*H*), 4.31 (2 H, s); **HRMS** *m*/*z* (ESI$^+$) calcd. for $C_{15}H_{14}FN_4$ [M+H]$^+$ requires 269.1197, found 269.1156. (Method B)

***N*-(4-Fluorobenzyl)-3-(pyridin-2-yl)-1*H*-pyrazol-5-amine, 8d**

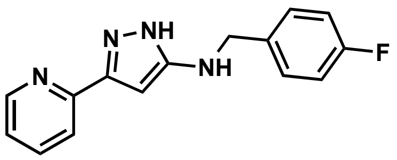

Isolated as a brown oil (76 mg, 0.28 mmol, 42%); **FT-IR** ν_{max} (ATR)/cm^{-1} 3368, 3140, 3048, 2909, 1578, 1539; ^{1}**H NMR** (400 MHz; CD_3OD) δ 8.49 (1 H, d, *J* 4.0), 7.80 – 7.72 (2 H, m), 7.44 (1H, t, *J* 7.0), 7.29 – 7.02 (4 H, m), 6.10 (1H, s), 4.40 (2 H, s); **HRMS** *m*/*z* (ESI$^+$) calcd. for $C_{15}H_{14}FN_4$ [M+H]$^+$ requires 269.1197, found 269.1184.(Method B)

3-(Pyridin-2-yl)-*N*-(pyridin-4-ylmethyl)-1*H*-pyrazol-5-amine, 8e

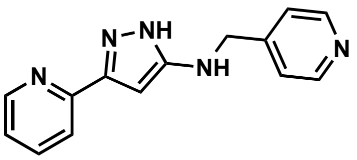

Isolated as a colourless solid (87 mg, 0.35 mmol, 52%); **DSC** 122 ºC; **FT-IR** ν_{max} (ATR)/cm^{-1} 3371, 2979, 2946, 2306, 2497, 1637, 1474, 1397; ^{1}**H NMR** (400 MHz; DMSO-d_6) δ 8.39 (2H, d, *J* 5.2), 7.75 (2 H, d, *J* 8.4), 7.68 (2H, d, *J* 6.0), 7.43 (2 H, d, *J* 8.4), 5.28 (1H, s, C*H*), 4.53 (2 H, s); **HRMS** *m*/*z* (ESI$^+$) calcd. for $C_{14}H_{14}N_5$ [M+H]$^+$ requires 252.1244, found 252.1234. (Method B)

***N*-(2-Chloro-6-fluorobenzyl)-3-(pyridin-2-yl)-1*H*-pyrazol-5-amine, 8f**

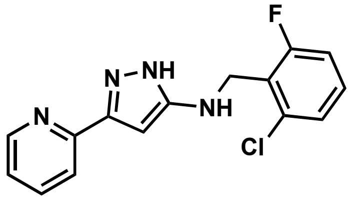

Isolated as a beige solid (91 mg, 0.30 mmol, 45%); **DSC** 113 ºC; **FT-IR** ν_{max} (ATR)/cm^{-1} 3238, 1599, 1567, 1518, 1460; ^{1}**H NMR** (500 MHz; CD_3OD) δ 8.52 (1H, d, *J* 4.0), 7.82 (1 H, td, *J* 8.0, 1.8), 7.75 (1H, d, *J* 8.0), 7.41 – 7.39 (2H, m), 7.31 – 7.24 (2H, m), 6.22 (1H, s), 4.60 (2H, s); **HRMS** *m*/*z* (ESI$^+$) calcd. for $C_{15}H_{13}{}^{35}ClFN_4$ [M+H]$^+$ requires 303.0807, found 303.0792. (Method B)

***N*-([1,1'-Biphenyl]-4-ylmethyl)-3-(pyridin-2-yl)-1*H*-pyrazol-5-amine, 8g**

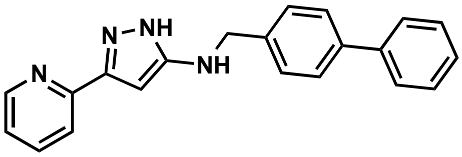

Isolated as a brown gum (129 mg, 0.40 mmol, 59%); **FT-IR** ν_{max} (ATR)/cm^{-1} 3373, 2983, 1653, 1595, 1456, 1382; ^{1}**H NMR** (500 MHz; DMSO-d_6) δ 8.01 – 7.99 (1 H, m), 7.92 – 7.90 (1 H, m), 7.77 – 7.75 (1H, m), 7.65 – 7.65 (4 H, m), 7.52 – 7.33 (4 H, m), 5.27 (1H, t, *J* 5.0), 4.54 (2H, d, *J* 5.0); **HRMS** *m/z* (ESI$^+$) calcd. for $C_{21}H_{19}N_4$ [M+H]$^+$ requires 327.1604, found 327.1594. (Method B)

3-(Pyridin-2-yl)-*N*-(4-(pyridin-4-yl)benzyl)-1*H*-pyrazol-5-amine, 8h

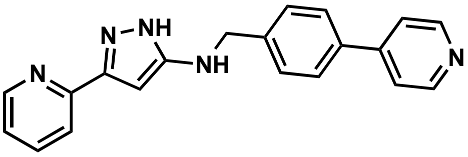

Isolated as a brown gum (134 mg, 0.41 mmol, 61%); **FT-IR** ν_{max} (ATR)/cm^{-1} 3217, 2979, 2946, 1738, 2603, 2497, 1598, 1475, 1398; ^{1}**H NMR** (400 MHz; DMSO-d_6) δ 7.95 – 7.88 (1 H, m), 7.73 – 7.58 (5 H, m), 7.48 – 7.31 (6H, m), 5.22 (1H, s), 4.50 (2H, s); **HRMS** *m/z* (ESI$^+$) calcd. for $C_{21}H_{19}N_4$ [M+H]$^+$ requires 328.1557, found 328.1537. (Method B)

3-(Pyridin-3-yl)pyrazol-5-amine, 11

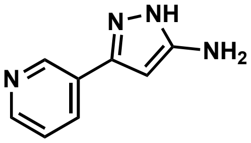

Under N_2 at room temperature, sodium hydride (60% mineral oil dispersion, 2.59 g, 64.8 mmol) was added to a solution of ethyl nicotinate (9.01 mL, 66.2 mmol) and acetonitrile (3.39 mL, 64.8 mmol) in anhydrous THF (110 mL). The resulting suspension was stirred for 16 h at 80 °C. The reaction mixture was cooled to room temperature, filtered, and washed with Et_2O (800 mL). The product was dried *in vacuo* to yield sodium (*E*/*Z*)-2-cyano-1-(pyridin-3-yl)ethen-1-olate (9.84 g) which was used without further purification.

A part of the crude product (8.00 g, 47.6 mmol) was dissolved in hydrochloric acid (1 M, 120 mL) and stirred at room temperature for 10 min. The pH value was adjusted to 5 with aqueous sodium hydroxide solution (2 M) and the aqueous phase was extracted with ethyl acetate (6 × 100 mL). The combined organic layers were dried over Na_2SO_4 and quickly reduced *in vacuo* as decomposition of the β-ketonitrile was observed. The residue was dissolved in ethanol (48 mL) and hydrazine hydrate (6.94 mL, 143 mmol) was added. The reaction mixture was stirred at 80 °C for 16 h. Following this time, the reaction mixture was cooled to room temperature reduced *in vacuo* and the residue was crystallised using ethanol and cyclohexane. The precipitate was collected by filtration and washed with cold ethanol (2 × 50 mL) giving 4.68 g of the title compound. The filtrate was concentrated *in vacuo*. The residue was diluted in methanol (40 mL) and adsorbed to Celite®. Purification by flash column chromatography (silica gel), eluting with methanol and dichloromethane (0:10 to 1:10) gave another 1.00 g of the title compound. Isolated as a colorless solid (5.68 g, 35.3 mmol, 53%): **R_f** 0.25 (CH_2Cl_2/CH_3OH 20:1); **FT-IR** ν_{max} 3389, 3049, 3876, 1630, 1614, 1576, 1516, 1468, 1194; **^{1}H NMR** (600 MHz, DMSO-d_6) δ 11.82 (1H, br), 8.88 (1H, dd, *J* 2.4, 0.9), 8.45 (1H, dd, *J* 4.8, 1.7), 8.01 (1H, dt, *J* 7.9, 1.9), 7.38 (1H, dd, *J* 7.9, 4.8), 5.75 – 5.90 (1H, br), 4.85 – 5.12 (2H, br); **^{13}C NMR** (151 MHz, DMSO-d_6) δ (ppm) 148.1, 146.0, 131.8, 123.7; Signals for

C-3$_{pyridyl}$, *C*-3$_{pyrazole}$, *C*-4$_{pyrazole}$ and *C*-5$_{pyrazole}$ are not observed. **HRMS** *m/z* (APCI$^+$) calcd. for $C_8H_9N_4$ [M+H]$^+$ requires 161.0822, found 161.0841; **HPLC** 3.30 min, 100%. (Method C)

_N_-[(1,1'-Biphenyl)-4-ylmethyl]-3-(pyridin-3-yl)pyrazol-5-amine, 13

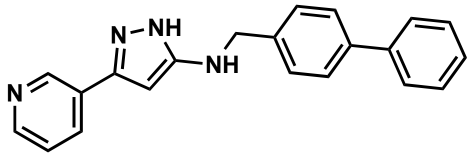

To a solution of biphenyl-4-carbaldehyde (170 mg, 927 µmol), acetic acid (100 µL) and 3 Å molecular sieves (100 mg, 100% *w*/*w*) in ethanol (anhydrous, 3 mL) was added 3-(pyridin-3-yl)-1*H*-pyrazol-5-amine (100 mg, 624 µmol). After 22h, sodium borohydride (95 mg, 2.50 mmol) was added. The reaction mixture was stirred for 16 h at room temperature. Following this time, aqueous sodium hydrogen carbonate solution (saturated, 5 mL) was added and the reaction mixture was extracted with ethyl acetate (3 × 30 mL). The combined organic layers were washed with brine (10 mL), dried over Na_2SO_4 and the solvent was removed *in vacuo*. The residue was diluted in methanol (5 mL) and adsorbed onto Celite®. Purification by flash column chromatography (silica gel), eluting with methanol and dichloromethane (0:10 to 1:20) gave the *title compound* a colourless solid (146 mg, 0.45 mmol, 72%); **mp.** 163 – 166 °C; **R$_f$** 0.42 (CH_2Cl_2/CH_3OH 20:1); **^{1}H NMR** (400 MHz, DMSO-*d*$_6$) δ 11.56 - 12.28 (1H, br), 8.92 (1H, dd, *J* 2.3, 0.9), 8.47 (1H, dd, *J* 4.8, 1.7), 8.03 (1H, ddd, *J* 7.8, 2.2, 1.8), 7.60 – 7.65 (2H, m), 7.59 – 7.64 (2H, m), 7.47 – 7.51 (2H, m), 7.41 – 7.46 (2H, m), 7.39 (1H, ddd, *J* 7.9, 4.7, 0.9), 7.33 (1H, tt, *J* 7.3, 1.2), 6.06 – 6.14 (1H, br), 5.95 – 6.02 (1H, br), 4.34 (2H, d, *J* 6.2); **^{13}C NMR** (101 MHz, DMSO-*d*$_6$) δ 148.2, 146.1,

140.1, 140.0, 138.5, 131.8, 128.9, 128.0, 127.2, 126.5, 126.5, 123.7, 47.4. Signals for *C*-3$_{pyridine}$, *C*-3$_{pyrazole}$, *C*-4$_{pyrazole}$, *C*-5$_{pyrazole}$ are not observed; **HRMS** *m/z* (APCI$^+$) calcd. for $C_{21}H_{19}N_4$ [M+H]$^+$ requires 327.1604, found 327.1624; **HPLC** 17.30 min, 99%. (Method C)

3-(2-Fluorophenyl)pyrazol-5-amine, 12

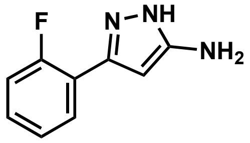

Under N_2 at -78 °C, acetonitrile (1.48 mL, 28.3 mmol) was added dropwise to a solution of *n*-butyllithium (1.6 M, 16.2 mL, 25.9 mmol) in THF (anhydrous, 15 mL) and the solution was stirred for 1 h at -78 °C. Methyl 2-fluorobenzoate (3.00 mL, 23.6 mmol) was added dropwise to the reaction mixture and the solution was stirred for 16 h while slowly warming up to room temperature. H_2O (20 mL) was added, followed by addition of HCl solution (1 m, 15 mL) until the reaction mixture was acidic. The aqueous layer was extracted with EtOAc (3 × 70 mL), the combined organic layers were washed with brine (10 mL) and dried over Na_2SO_4. The solvent was removed *in vacuo*. The residue was diluted in ethyl acetate (5 mL) and adsorbed to Celite®. Purification by flash column chromatography (silica gel), eluting with ethyl acetate and cyclohexane (0:10 to 6:4) gave the 3-(2-fluorophenyl)-3-oxopropanenitrile as a colorless solid (3.49 g, 21.4 mmol, 91%); **mp.** 37 – 39 °C; **R$_f$** 0.45 (EtOAc:*c*Hex 1:2); **FT-IR** ν_{max} 2959, 2928, 1686, 1605, 1474, 1450, 1331, 1196; **^{1}H NMR** (600 MHz, $CDCl_3$) δ 7.96 (1H, td, *J* 7.8, 2.0) 7.64 (1H, m), 7.31 (1H, m), 7.20 (1H, ddd, *J* 11.5, 8.5, 1.0), 4.10 (2H, d, *J* 2.5); **^{13}C NMR** (151 MHz, $CDCl_3$): δ 185.2 (d, *J* 4.1), 162.3 (d, *J* 257.0), 136.7 (d, *J* 9.4), 131.3 (d, *J* 2.3), 125.3 (d,

J = 3.1), 122.8 (d, *J* 13.2), 117.0 (d, *J* 24.4), 113.6, 33.8 (d, *J* 11.6); **HRMS** *m/z* (APCI$^+$) calcd. for $C_8H_7FO_2$ $[M+H]^+$ requires 164.0506, found 164.0527; **HPLC** 14.70 min, 94% (Method C).

To a solution of 3-(2-fluorophenyl)-3-oxopropanenitrile (3.41 g, 20.9 mmol) in ethanol (35 mL) was added hydrazine hydrate (1.57 mL, 31.4 mmol) dropwise. The reaction mixture was stirred for 16 h at 90 °C. H_2O (20 mL) was added and the pH value adjusted to 7 by slowly adding hydrochloric acid (1 M). The mixture was extracted with ethyl acetate (6 × 50 mL) The combined organic layers were dried over Na_2SO_4 and the solvent was removed *in vacuo*. The residue was diluted in methanol (5 mL) and adsorbed onto Celite®. Purification by flash column chromatography (silica gel), eluting with methanol and dichloromethane (0:10 to 1:20) gave the title compound as an orange solid (3.01 g, 17.0 mmol, 81 %); **mp.** 89 - 91 °C; **R$_f$** 0.42 (CH_2Cl_2/CH_3OH 20:1); **FT-IR** ν_{max} (ATR)/cm^{-1} 3360, 3264, 2874, 1620, 1589, 1504, 1477, 1447, 1254, 1219; **^{1}H NMR** (600 MHz, $CDCl_3$) δ 7.59 (1H, td, *J* 7.8, 1.7), 7.28 (1H, dddd, *J* 8.2, 7.2, 5.3, 1.7), 7.17 (1H, td, *J* 7.6, 1.2), 7.14 (1H, ddd, *J* 11.8, 8.3, 1.2), 6.04 (1H, d, *J* 1.1), 5.61 – 5.84 (2H, br); **^{13}C NMR** (151 MHz, $CDCl_3$) δ 159.5 (d, *J* 247.9), 154.6 (d, *J* 1.5), 139.6 (d, *J* 2.2), 129.7 (d, *J* 8.7), 127.9 (d, *J* 4.0), 124.9 (d, *J* 3.0), 117.6 (d, *J* 12.2), 116.5 (d, *J* 21.8), 92.0 (d, *J* 3.3); **HRMS** *m/z* (APCI$^+$) calcd. for $C_9H_9FN_3$ $[M+H]^+$ requires 178.0775, found 178.0782; **HPLC** 11.40 min, 99% (Method C).

_N_-[(1,1'-Biphenyl)-4-ylmethyl]-3-(2-fluorophenyl)pyrazol-5-amine, 14

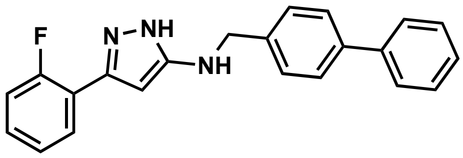

To a solution of biphenyl-4-carbaldehyde (154 mg, 847 μmol), acetic acid (100 μL) and 3 Å molecular sieves (100 mg, 100% *w/w*) in ethanol (anhydrous, 3 mL) was added 3-(2-fluorophenyl)-1-pyrazol-5-amine (100 mg, 574 μmol). After 22h, sodium borohydride (85.4 mg, 2.26 mmol) was added. The reaction mixture was stirred overnight at room temperature. Following this time, sodium hydrogen carbonate solution (saturated, 5 mL) was added and the reaction mixture was extracted with ethyl acetate (3 x 30 mL). The combined organic layers were washed with brine (10 mL), dried over Na_2SO_4 and the solvent was removed *in vacuo*. The residue was diluted in methanol (5 mL) and adsorbed onto Celite®. Purification by flash column chromatography (silica gel), eluting with methanol and dichloromethane (0:10 to 1:20) gave the *title compound* as a colorless solid (137 mg, 0.39 mmol, 69%); **mp.** 163 - 166 °C; **R_f** 0.37 (20:1 CH_2Cl_2/CH_3OH); **FT-IR** ν_{max} (ATR)/cm^{-1} 3426, 3094, 2882, 1539, 1512, 1485, 1211; **^{1}H NMR** (400 MHz, DMSO-d_6) δ (ppm) 11.56 - 12.28 (1H, br), 8.92 (1H, dd, *J* 2.3, 0.9), 8.47 (1H, dd, *J* 4.8, 1.7), 8.03 (1H, ddd, *J* = 7.8, 2.2, 1.8), 7.59 - 7.64 (2H, m), 7.60 - 7.65 (2H, m), 7.47 - 7.51 (2H, m), 7.41 - 7.46 (2H, m, 2H), 7.39 (1H, ddd, *J* 7.9, 4.7, 0.9), 7.33 (1H, tt *J* 7.3, 1.2), 6.06 - 6.14 (1H, br), 5.95 - 6.02 (1H, br), 4.34 (2H, d, *J* 6.2); **^{13}C NMR** (101 MHz, DMSO-d_6) δ (ppm) 148.2, 146.1, 140.1, 140.0, 138.5, 131.8, 128.9, 128.0, 127.2, 126.5, 126.5, 123.7, 47.4 Signals for *C*-1_{PhF}, *C*-$3_{pyrazole}$, *C*-$4_{pyrazole}$ and *C*-$5_{pyrazole}$ are not observed; **HRMS** *m/z* (APCI$^+$) calcd. for $C_{22}H_{19}FN_3$ $[M+H]^+$ requires 344.1558, found 344.1580; **HPLC** 20.30 min, 99%.
