# Supplementary figures and images for: Collaborative Synthesis for Neglected Diseases through the Open Synthesis Network: Structure–Activity Relationships of Arylaminopyrazoles as Chagas Disease Treatments
Source: ACS Infect Dis. 2025 Aug 11;11(9):2593–606. doi: 10.1021/acsinfecdis.5c00481 (PMC12442103; doi:10.1021/acsinfecdis.5c00481)

For Table of Contents Use Only

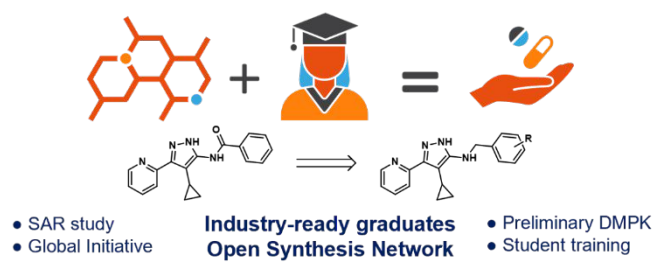

Supplement: Supplementary file 3 [file id5c00481_si_003.pdf]
